# *NAT2* gene polymorphisms and endometriosis risk: A PRISMA-compliant meta-analysis

**DOI:** 10.1371/journal.pone.0227043

**Published:** 2019-12-27

**Authors:** Zhangming Wei, Mengmeng Zhang, Xinyue Zhang, Mingyu Yi, Xiaomeng Xia, Xiaoling Fang

**Affiliations:** Department of Obstetrics and Gynecology, The Second Xiangya Hospital, Central South University, Changsha, Hunan, P.R. China; IRCCS San Raffaele Scientific Institute, ITALY

## Abstract

**Objective:**

Endometriosis is a common chronic, gynecological disease. Despite many studies on the role of *N*-acetyltransferase 2 (NAT2) in endometriosis, its clinical significance is unclear. In this study, associations between NAT2 phenotypes as well as single nucleotide polymorphisms (SNPs) within *NAT2* (i.e. rs1799929, rs1799930, rs1208, and rs1799931) and endometriosis risk were evaluated using a meta-analysis approach.

**Methods:**

Embase, PubMed, ClinicalTrials.gov, CNKI (China National Knowledge Infrastructure), Wanfang databases, Cochrane Library for clinical trials, and Web of Science were searched to identify relevant articles. ORs (odds ratios) and 95% CIs (95% confidence intervals) were used to estimate the associations between *NAT2* polymorphisms and endometriosis risk. Heterogeneity among included studies was also assessed. In addition, a subgroup analysis of NAT2 phenotypes and endometriosis risk based on ethnicity was performed.

**Results:**

Nine case-control studies met the inclusion criteria. The odds ratio was 2.30 (95% CI: 1.61–3.28) for the NAT2 slow acetylation phenotype versus the intermediate + fast acetylation phenotype in the Asian population. These results suggest that Asian individuals with the NAT2 slow acetylation phenotype have a 130% increased risk of endometriosis. A significant association was also found for rs1799930 (OR = 0.74; 95% CI, 0.59–0.92), suggesting that individuals with this mutant genotype have a 26% decreased risk of endometriosis.

**Conclusions:**

The rs1799930 mutant genotypes are associated with a decreased risk of endometriosis. No statistically significant associations were found between rs1799931, rs1208, or rs1799929 and endometriosis. Based on a subgroup analysis based on ethnicity, the NAT2 slow acetylation phenotype was found to increase the risk of endometriosis in Asians. No statistically significant associations were found between the NAT2 slow acetylation phenotype and endometriosis risk in Caucasians. Accordingly, NAT2 phenotypes and SNPs are potential biomarkers for the diagnosis and treatment of endometriosis.

## Introduction

Endometriosis is a common estrogen-dependent chronic gynecological disease affecting 5–10% of women of reproductive age[1]. It occurs when the endometrium grows outside of the uterine corpus, which causes inflammation, pelvic pain, dysmenorrhea, menstrual disorders, ectopic bleeding, bladder symptoms, infertility, and further malignant transformation [2]. It is strongly associated with significant social and physical debility. The etiology of this disease is unclear. However, the main hypotheses include classical theories of blood reflux, blood and lymphatic dissemination, coelomic metaplasia, immunology, endocrinology, and genetics, but none of these can satisfactorily explain the occurrence of endometriosis [3].

With increasing evidence for a role of genetic factors, many scholars are trying to find genes related to the pathogenesis of endometriosis [4]. Recently, toxic metabolic enzyme genes have been a focus of research. Genes encoding many metabolic enzymes such as cytochrome P4501A1 (CYP1A1), catechol-*O*-methyltransferase (COMT), *N*-acetyltransferase 2 (NAT2), and glutathione *S*-transferase P1 (GSTP1) are associated with polymorphisms that can distinguish between populations. Mutations might be related to the decreased function or changes in the function of these enzymes including enzymes involved in detoxification, resulting in differences in the risk of endometriosis and other diseases [5]. Recent meta-analysis-based studies have investigated the relationships between polymorphisms in *GSTP1* [6], *COMT* [7], and *CYP1A1* [8] and the risk of endometriosis. Here, we further analyzed the relationship among NAT2 phenotypes, genotypes, and endometriosis.

NAT2 is the product of a single, intron-less gene comprising an 870-bp open reading frame that encodes 290 amino acids. The gene is located on chromosome 8p21.323.1 [9]. *NAT2* nucleotide substitutions can change the protein structure and cause reductions in substrate affinity, protein stability, and/or catalytic activity for the recombinant N-acetyltransferase allozyme. Recombinant human NAT2 clusters catalyze N-, O-, and N, O-acetyltransferase activities at slow speeds compare to that with wild-type phenotype [10, 11].

Extensive research has concretely established the correlation between *NAT2* polymorphisms and susceptibility to a variety of complex diseases, particularly in lung cancer, bladder cancer, alimentary canal tumor, asthma, and other allergic disorders [12]. *NAT2* has over 27 variants or combinations of single nucleotide polymorphisms (SNPs) [13]. The studied SNPs in *NAT2* that affect its phenotype include rs1799930, also known as G590A, rs1799931, also known as G857A, rs1799929, also known as C481T, and rs1208, also known as A803G [14–17]. Like that in leukemia, different combinations of SNPs result in different alleles, producing the NAT2 slow, intermediate, or fast acetylation phenotypes, as summarized by the authoritative NAT2 organization websites “http://nat2pred.rit.albany.edu/” and http://nat.mbg.duth.gr/Human%20NAT2%20alleles_2013.htm#_Footnotes [14].

Endometriosis is associated with a range of environmental factors (hormonal/reproductive, lifestyle) and genetic factors. It is well established that high levels of natural and man-made chemicals are present in the environment and play a role in the pathogenesis of endometriosis. For example, dioxin-like PCBs (polychlorinated biphenyls) promote the development of endometriosis through the stimulation of endocrine–inflammation interactions. Toxicant dioxins (dioxin and dioxin-like chemicals) have an adverse effect on growth factors, cytokines, hormones, and the immune system. Exposure to dioxins is a potential factor for the development of endometriosis [18, 19]. Because endometriosis is a hormone-dependent disease, polymorphisms in genes encoding detoxification enzymes might contribute to its development [20–23]. NAT2 plays a key role in xenobiotic metabolism. Other xenobiotic metabolic enzymes like aromatase P450 family members CYP1A1 and CYP19A1 [24] also have a fundamental role in the pathogenesis of endometriosis [23, 25], and have been widely studied and applied to endometriosis treatment. However, the role of *NAT2* polymorphisms in endometriosis remains to be discovered. Specifically, previous studies have evaluated the relationship between *NAT2* polymorphisms and the risk of endometriosis; however, contradictory results have been obtained [14, 26, 27]. Accordingly, in this study, a meta-analysis was performed to clarify whether different NAT2 phenotypes or the SNPs rs1799929, rs1799930, rs1208, and rs1799931 are associated with susceptibility to endometriosis worldwide.

## Materials and methods

The detailed protocol, which followed the template of the Cochrane review is available in the PROSPERO registry (No. CRD42018111924). This meta-analysis was prepared according to the Preferred Reporting Items for Systematic Reviews and Meta-Analysis (PRISMA) guidelines.

Declaration of human rights: no formal consent was required for this type of study.

### Search strategy

PubMed, Embase, Web of science, Cochrane Library for clinical trials, ClinicalTrials.gov, CNKI (China National Knowledge Infrastructure), and Wanfang databases were searched extensively (the last search was updated on June 20, 2019). The search words and strategy included the following: (NAT2 OR "N-acetyltransferase 2" OR "Arylamine-N-acetyltransferase" OR "NAT" OR "dioxin detoxification enzymes") AND (mutation OR variant OR polymorphism) AND (endometriosis OR endometriosis OR Mulleriosis OR Mullerianosis OR EM OR EMT OR EMS OR Mullerianosis).

### Inclusion and exclusion criteria

Inclusion criteria were as follows. (1) The relationship between *NAT2* polymorphisms and endometriosis risk was evaluated. (2) Only case-control studies that included both endometriosis cases and endometriosis-free controls were included. (3) Sufficient and procurable data for both cases and controls were required to estimate the odds ratio (OR) and 95% confidence interval (95% CI). (4) Endometriosis diagnosis in accordance with the Revised American Society for Reproductive Medicine classification was required. The exclusion criteria were as follows: (1) abstracts, case reports, letters, reviews, or single-arm studies; (2) phenotype/genotype frequency and endometriosis risk were not reported; (3) incomplete data for the calculation of ORs and 95% CIs.

### Data extraction

All potentially relevant studies were checked by two investigators (M-M.Z. and Z-M.W.) independently, and a third investigator (X-L.F.) resolved discrepancies. The following items were extracted: year of publication, first author, diagnostic standard, NAT2 phenotypes, target genotypes, diagnostic methods, genotyping methods, case age, ethnicity, features of the controls, endometriosis stage, genotype distributions in cases and controls, and the total number of cases and controls. The corresponding authors of the original studies were contacted when further data were needed.

### Statistical analysis

Subgroup analysis by ethnicity, χ^2^ tests, Begg’s funnel plots, and Egger’s tests were performed. In addition, the Newcastle-Ottawa scale (NOS), OR, 95% CI, and I^2^ statistic were estimated. The NOS criteria were used to assess the methodological quality of all studies. Studies with NOS scores ≥ 7 were regarded as good quality (range, 0–8) [28]. To determine whether genotypes were in accordance with the Hardy–Weinberg equilibrium, an internet-based program was used (http://ihg.gsf.de/cgi-bin/hw/hwa1.pl) [29]. The risk for the primary model (slow + intermediate phenotype versus fast phenotype) was first evaluated. Then, the recessive model (slow phenotype versus intermediate + fast phenotype) was evaluated. In addition, the risks of the intermediate phenotype versus the fast phenotype and the slow phenotype versus the fast phenotype were estimated. Moreover, a subgroup analysis by ethnicity (Asian or Caucasian) was performed. For each SNP, all combinations of genotypes were evaluated. The *I*^2^ statistic was used to calculate heterogeneity. A value of P < 0.05 was regarded as statistically significant. The Bonferroni method was used to adjust P values for multiple testing. If heterogeneity was low (*I*^2<^30%), a fixed effect model was used to calculate the combined OR for each study. Otherwise (*I*^2^ ≥ 30%), the random effect model was used. The publication bias was assessed by Begg’s and Egger’s tests. A sensitivity analysis was performed by sequentially excluding each study to assess the stability of the meta-analysis results. Environmental effects adjustments, like those for dioxin exposure, pollution exposure, life style, diabetes, smoking, coffee intake, breast feeding time, or other genetic factors were not conducted due to the insufficiency of available data. All analyses were implemented with Stata 12.0 (Stata, College Station, TX, USA) and RevMan 5.3 software (Cochrane Collaboration, Oxford, England) [30].

## Results

### Summary of study characteristics

**Fig 1:**
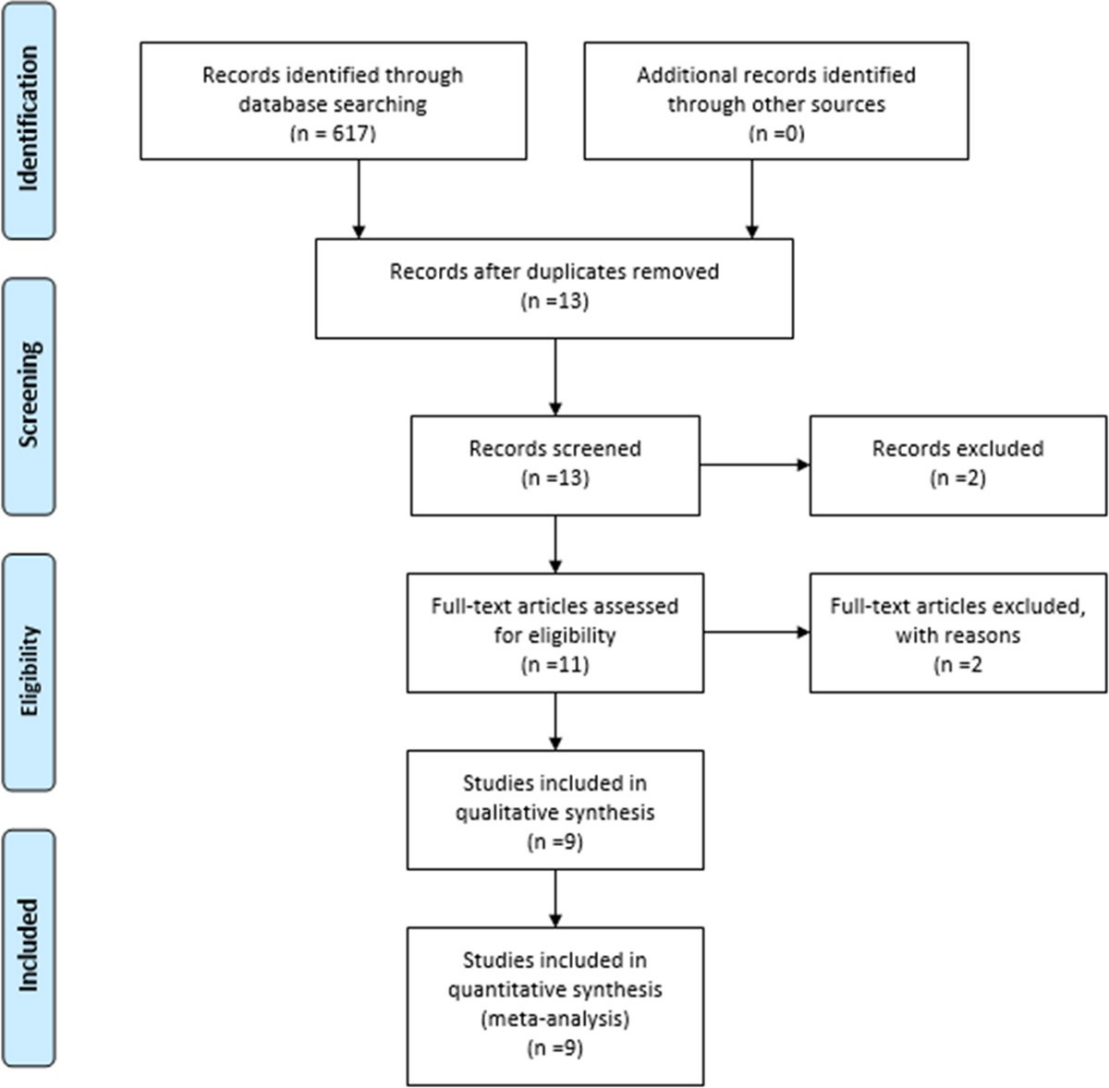
Flow chart of literature search and study selection. Nine case-control studies were included in this meta-analysis.

A total of 617 articles were retrieved from the electronic search. Among these, 604 were excluded based on titles and abstracts. The full texts of the remaining 13 articles were screened. One review article was excluded [25]. Two articles were excluded for not reporting exact genotypes [20, 31]. One article was excluded for being a single-arm study [32]. Finally, nine case-control studies [14, 17, 26, 27, 33–37] met the inclusion criteria. The details of the study selection process are presented in Fig 1. All article titles excluded—with reason—are shown in S1 File. The characteristics and quality of the included studies are summarized in Tables 1 and 2.

**Table 1:** Characteristics and distribution of NAT2 phenotype and SNPs polymorphisms of the 9 case–control studies included in the meta-analysis.

				Case					Control					Diagnosis	Staging	Control	NOS
polymorphism	Reference	Country	Race	mm	mw	ww	m	w	mm	mw	ww	m	w	Method	Info	Selection	score
NAT2 phenotype	Babu 2004[26]	India	Caucasian	164	79	9	-	-	176	80	8	-	-	Surgical	Y	Hospital	8
	baranova1999[27]	France	Caucasian	39	23	3	-	-	28	35	9	-	-	Surgical	Y	Hospital	8
	Cao 2007[35]	China	Asian	41	37	16	-	-	21	35	46	-	-	Surgical	Y	Hospital	8
	Chen 2003[36]	China	Asian	10	37	28	-	-	6	43	31	-	-	Surgical	Y	Hospital	8
	Chen 2009[37]	China	Asian	36	30	14	-	-	17	27	36	-	-	Surgical	Y	Hospital	8
	Fayez 2018[14]	Iran	Caucasian	37	55	49	-	-	62	52	44	-	-	Surgical	Y	Hospital	8
	ivaschenko2003[33]	Russia	Caucasian	39	23	11	-	-	18	15	7	-	-	Surgical	Y	Hospital	8
	Deguchi 2005[17]	Japan	Asian	29	78	113	-	-	16	76	80	-	-	Surgical	Y	Community	7
	Nakago 2001[34]	UK	Caucasian	23	28	3	-	-	83	34	6	-	-	Surgical	Y	Community	7
G590A	Babu 2004[26]	India	Caucasian	33	113	106	179	325	36	132	96	204	324	Surgical	Y	Hospital	8
	Chen 2003[36]	China	Asian	4	26	45	34	116	1	34	40	36	114	Surgical	Y	Hospital	8
	Fayez 2018[14]	Iran	Caucasian	17	54	70	88	194	19	87	52	125	191	Surgical	Y	Hospital	8
	Deguchi 2005[17]	Japan	Asian	7	70	143	84	356	6	56	110	68	276	Surgical	Y	Community	7
	Nakago 2001[34]	UK	Caucasian	0	2	52	2	106	0	5	118	5	241	Surgical	Y	Community	7
G857A	Chen 2003[36]	China	Asian	1	18	56	20	130	1	16	63	18	142	Surgical	Y	Hospital	8
	Fayez 2018[14]	Iran	Caucasian	5	63	73	73	209	12	72	74	96	220	Surgical	Y	Hospital	8
	Deguchi 2005[17]	Japan	Asian	4	35	181	43	397	3	27	142	33	311	Surgical	Y	Community	7
	Nakago 2001[34]	UK	Caucasian	1	32	21	34	74	17	47	59	81	165	Surgical	Y	Community	7
C481T	Babu 2004[26]	India	Caucasian	34	94	124	162	342	31	116	117	178	350	Surgical	Y	Hospital	8
	Chen 2003[36]	China	Asian	0	3	72	3	147	0	2	80	2	162	Surgical	Y	Hospital	8
	Fayez 2018[14]	Iran	Caucasian	27	77	37	131	151	32	88	38	152	164	Surgical	Y	Hospital	8
A803G	Babu 2004[26]	India	Caucasian	35	103	114	173	331	36	123	105	195	333	Surgical	Y	Hospital	8
	Fayez 2018[14]	Iran	Caucasian	58	58	25	174	108	65	73	20	203	113	Surgical	Y	Hospital	8

Note: For NAT2 mm = slow acetylation phenotype, wm = intermediate acetylation phenotype, ww = fast acetylation phenotype. For each SNPs, m = mutation alleles, w = wild alleles, mm = mutation homozygote, mw = mutation heterozygote, ww = wild homozygote. for example: for G590A, m = A, w = G, mm = AA, mw = AG, ww = GG.

**Table 2:** The Newcastle-Ottawa Scale (NOS) checklist of included studies.

Study	Score	Cohort selection				Comparability	Outcome ascertainment		
		Represen-tativeness of the Exposed Cohort	Selection of the Non-Exposed Cohort	Ascertain-ment of Exposure	Demonstration that Outcome of Interest Was Not Present at Start of Study	Comparability of Cases and Controls on the Basis of the Design or Analysis	Assess-ment of Outcome	Was Follow-Up Long Enough for Outcomes to Occur	Adequacy of Follow Up of Cohorts
Fayez 2018[14]	8	1	1	1	1	1	1	1	1
Chen 2009[37]	8	1	1	1	1	1	1	1	1
Cao 2007[35]	8	1	1	1	1	1	1	1	1
Deguchi 2005[17]	7	1	0	1	1	1	1	1	1
Babu 2004[26]	8	1	1	1	1	1	1	1	1
Chen 2003[36]	8	1	1	1	1	1	1	1	1
ivaschenko2003[33]	8	1	1	1	1	1	1	1	1
Nakago 2001[34]	7	1	0	1	1	1	1	1	1
baranova1999[27]	8	1	1	1	1	1	1	1	1

### Quantitative data analysis

#### NAT2 phenotype and endometriosis risk

**Fig 2:**
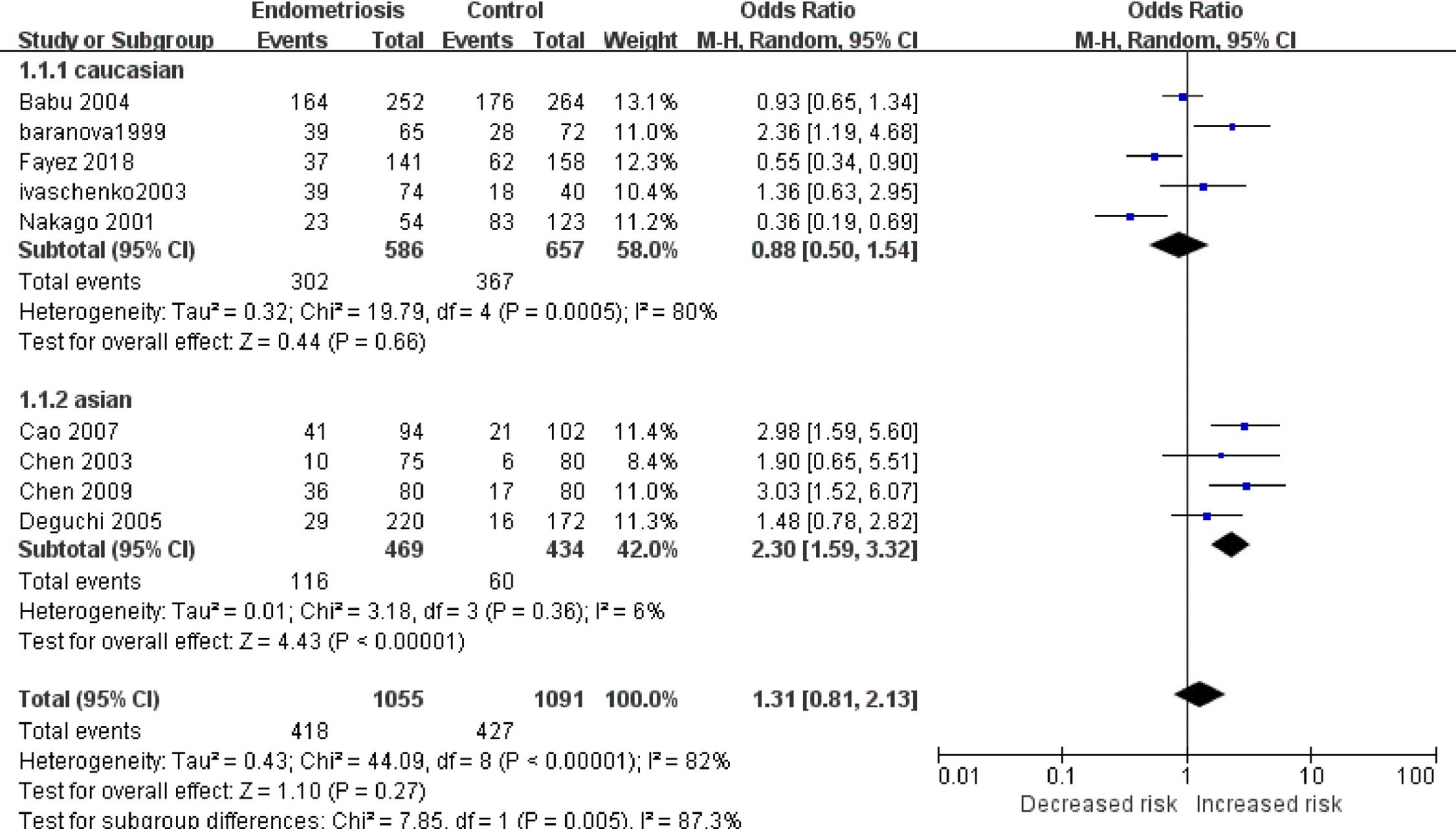
Meta-analysis for the association between NAT2 phenotypes and endometriosis risk (slow + intermediate versus fast). The result indicates Asian individuals who present NAT2 slow acetylation phenotype might have a 130% increased endometriosis risk.

In the primary model (slow vs. intermediate + fast), heterogeneity was high (χ^2^ = 44.09, *I*^2^ = 82%; Fig 2). Accordingly, we performed a subgroup analysis focusing on different ethnicities, mainly a Caucasian group and an Asian group. In the Asian group, the heterogeneity was low (χ^2^ = 3.18, *I*^2^ = 6%) and the OR was 2.30 (95% CI, 1.59–3.32; P < 0.001). This result suggests that individuals with the NAT2 slow acetylation phenotype have a 130% increased risk of endometriosis in the Asian population. Results for other ethnicity groups are summarized in Table 3.

**Table 3:** Summary of results in different NAT2 genotype comparative models.

Comparison model	Overall or subgroup	Study number (n)	Total (n)	OR(95%CI)	Z	P	*I*^2^ (%)	P_het_	Effect model
Slow vs Intermediate+Fast	Total	9	2146	1.31(0.81,2.13)	1.1	0.27	82	0.001	R
	Caucasian	5	1243	0.88(0.50,1.54)	0.44	0.66	80	0.001	R
	Asian	4	903	2.30(1.59,3.32)	4.58	**0.001**	6	0.36	R
Slow+Intermediate vs Fast	Total	9	2146	1.41(0.86,2.31)	1.36	0.17	75	0.001	R
	Caucasian	5	1243	0.91(0.63,1.31)	0.51	0.61	1	0.4	F
	Asian	4	903	1.86(0.80,4.36)	1.44	0.15	88	0.001	R
Slow vs Fast	Total	9	2146	1.68(0.87,3.26)	1.54	0.12	79	0.001	R
	Caucasian	5	1243	0.96(0.49,1.89)	0.11	0.92	53	0.07	R
	Asian	4	903	2.80(1.88,4.19)	5.05	**0.001**	73	0.01	R
Intermediate vs Fast	Total	9	2146	1.14(0.90,1.45)	1.10	0.27	56	0.02	R
	Caucasian	5	1243	1.05(0.70,1.58)	0.24	0.81	0	0.85	F
	Asian	4	903	1.19(0.89,1.60)	1.18	0.24	82	0.001	R

Note

OR = Odds ratio.

CI = Confidence interval.

Z = Z-value for Q statistic.

P = P-value for Q statistic.

I^2^ = I statistic for heterogeneity.

Phet = P-value for heterogeneity.

F = Fixed model.

R = Random model.

#### Associations between SNPs and the risk of endometriosis

**Fig 3:**
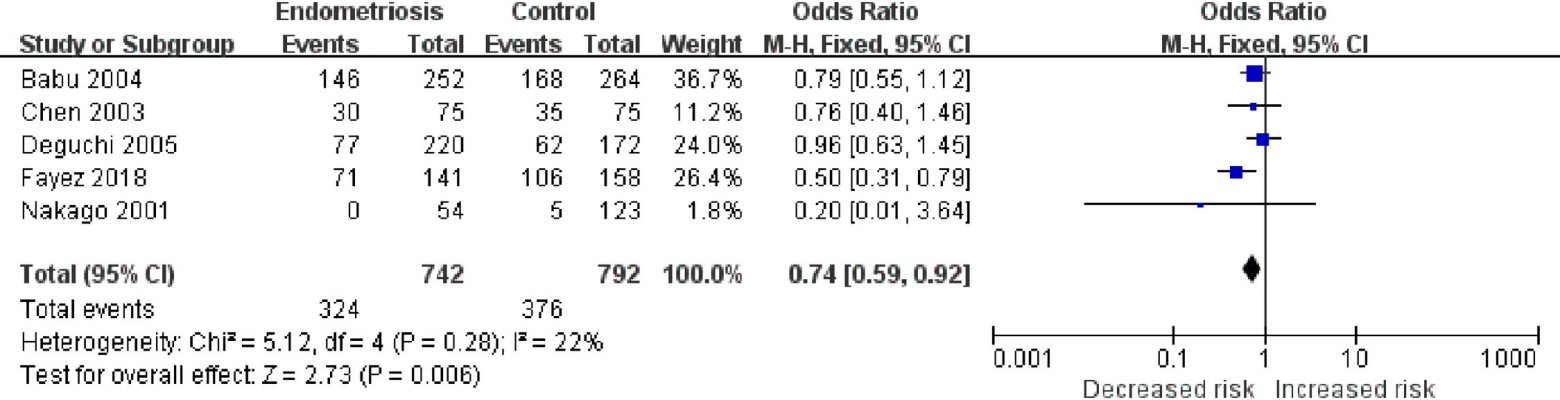
Meta-analysis for the association between G590A polymorphism and endometriosis risk (AA + AG versus GG). People who carry G590A mutation may have 26% decreased endometriosis risk compared with wild homozygotes.

**Fig 4:**
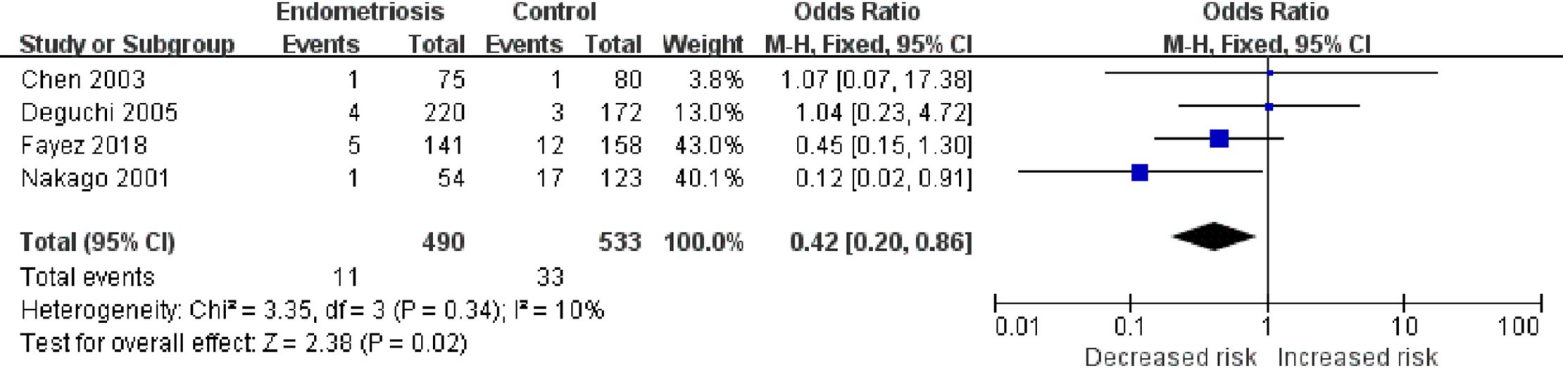
Meta-analysis for the association between G857A polymorphism and endometriosis risk (AA versus AG+GG).

Next, we investigated the relationships between rs1799930 (G590A), rs1799931 (G857A), rs1799929 (C481T), or rs1208 (A803G) and the risk of endometriosis. As shown in Fig 3, in the G590A AA+GA vs. GG model, heterogeneity was low (χ^2^ = 5.12, *I*^2^ = 22%) and the OR was 0.74 (95% CI, 0.59–0.92; P < 0.001; P_Adjust_ = 0.03), indicating that individuals who carry the G590A mutation have a 26% decreased risk of endometriosis compared to that of wild-type homozygotes. In the G857A AA vs. AG+GG model (Fig 4), heterogeneity was low (χ^2^ = 3.35, *I*^2^ = 10%) and the OR was 0.42 (95% CI, 0.20–0.86; P = 0.02; P_adjust_ = 0.10). For C481T and A803G, no statistically significant differences in risk were detected (Table 4). All model comparisons and results are summarized in Table 4.

**Table 4:** Summary of results in different SNP phenotype comparative models.

SNP	Comparison model	Study number(n)	Total (n)	OR(95%CI)	Z	P	P_Adjust_	I^2^ (%)	P_het_	Effect model
G590A	AA+AGvsGG	5	1534	**0.74(0.59,0.92)**	2.73	**0.006**	**0.03**	22	0.28	F
	AAvsAG+GG	5	1534	1.02(0.70,1.49)	0.10	0.92	4.6	0	0.65	F
	AAvsGG	5	955	0.84(0.56,1.25)	0.87	0.38	1.9	0	0.65	F
	AGvsGG	5	1411	0.73(0.58,0.91)	2.75	0.006	0.03	22	0.28	F
G857A	AA+AGvsGG	4	1023	1.03(0.78,1.37)	0.23	0.82	4.1	0	0.51	F
	AAvsAG+GG	4	1023	**0.42(0.20,0.86)**	2.38	**0.02**	**0.1**	10	0.34	F
	AAvsGG	4	713	0.46(0.22,0.97)	2.05	0.04	0.2	0	0.48	F
	AGvsGG	4	979	1.12(0.84,1.50)	0.79	0.43	2.15	17	0.31	F
C481T	TT+TCvsCC	3	972	1.06(0.72,1.55)	0.28	0.78	3.9	0	0.56	F
	TTvsTC+CC	3	972	0.86(0.64,1.14)	1.06	0.29	1.45	0	0.74	F
	TTvsCC	3	592	0.97(0.63,1.48)	0.16	0.87	4.35	0	0.69	F
	TCvsCC	3	848	0.82(0.61,1.11)	1.27	0.2	1	0	0.66	F
A803G	GG+GAvsAA	2	815	0.77(0.57,1.04)	1.69	0.09	0.45	0	0.64	F
	GGvsGA+AA	2	815	1.01(0.72,1.42)	0.06	0.96	4.8	0	0.95	F
	GGvsAA	2	458	0.82(0.54,1.25)	0.91	0.36	1.8	0	0.61	F
	GAvsAA	2	621	0.74(0.53,1.02)	1.82	0.07	0.35	0	0.63	F

Note

OR = Odds ratio.

CI = Confidence interval.

Z = Z-value for Q statistic.

P = P-value for Q statistic.

I^2^ = I statistic for heterogeneity.

Phet = P-value for heterogeneity.

F = Fixed model.

R = Random model.

#### Publication bias

**Fig 5:**
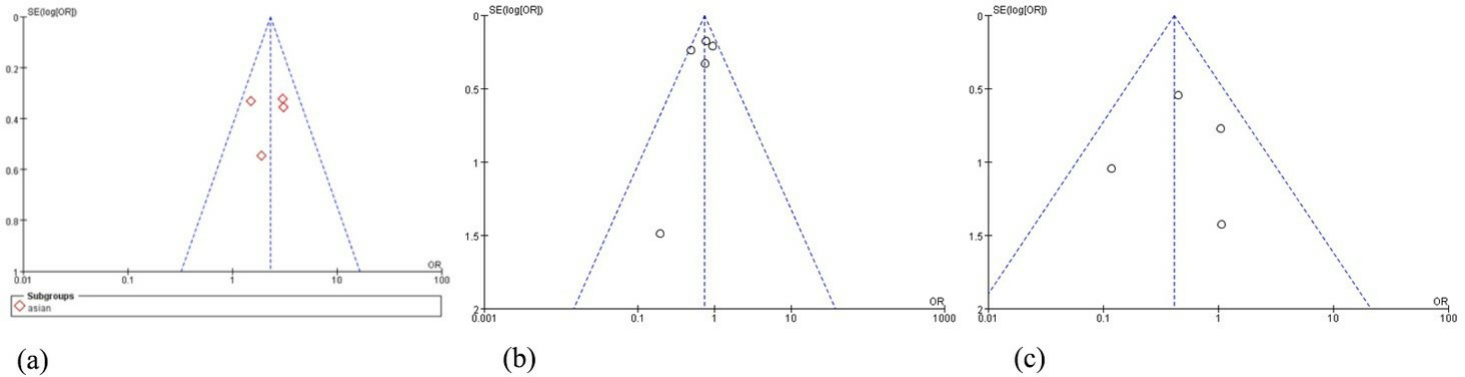
Begg’s funnel plot for publication bias in the selection of studies. (a) on NAT2 phenotype polymorphism in Asian group, (b) on G590A polymorphism, and (c) on G857A polymorphism. Begg's funnel plot indicates low publication bias of this study.

Begg’s funnel plot and Egger’s test were used to evaluate publication bias. The symmetry detected in Begg’s funnel plot indicated low publication bias in these statistically significant models (Fig 5).

#### Sensitivity analysis

By sequentially excluding individual studies, the outcomes were consistent with the overall study results, indicating that our results showed good stability and reliability. After excluding each study, the changes in OR with 95% CIs are presented in Table 5.

**Table 5:** Summary of sensitivity analysis results when excluding each study.

Comparison model	Excluded Study	OR[95%CI]	Z	*I*^2^ (%)	Effect model
NAT2 phenotype in Asian group	Cao2007[35]	2.03 [1.32, 3.13]	3.04	10	F
	Chen2003[36]	2.35 [1.61, 3.43]	3.04	34	R
	Chen2009[37]	2.08 [1.37, 3.15]	3.13	15	F
	Deguchi2005[17]	2.79 [1.82, 4.27].	4.71	0	F
G590A AA + AG versus GG model	Babu2004[26]	0.71 [0.54, 0.94]	2.43	39	R
	Chen2003[36]	0.73 [0.58, 0.93]	2.61	41	R
	Deguchi2005[17]	0.67 [0.52, 0.86]	3.07	5	F
	Fayez2018[14]	0.82 [0.64, 1.05]	1.54	0	F
	Nakago2001[34]	0.75 [0.60, 0.93]	2.60	31	R
G857A AA versus AG+GG model	Chen2003[36]	0.39 [0.18, 0.83]	2.44	34	R
	Nakago2001[34]	0.62 [0.27, 1.39]	1.17	0	F
	Fayez2018[14]	0.39 [0.15, 1.05]	1.87	42	R
	Deguchi2005[17]	0.32 [0.14, 0.76]	2.58	0	F

Note

OR = Odds ratio.

CI = Confidence interval.

Z = Z-value for Q statistic.

I^2^ = I statistic for heterogeneity.

F = Fixed model.

R = Random model.

## Discussion

Endometriosis is one of the most common diseases in women [38]. However, its etiology and pathogenesis have not been fully elucidated [39]. It has been proven that endometriosis exhibits polygenic inheritance. Evidence suggests that its occurrence and development involve genetic variation, abnormal regulation, or mutation accumulation, but the exact genetic basis remains to be discovered. Accordingly, studies of genetic susceptibility to endometriosis and screening for susceptibility genes are increasing [40].

Recently, some scholars have proposed a theory for the inheritance and induction of endometriosis, suggesting that it is a multi-factorial genetic disease caused by the cumulative effects of mutations at multiple loci and environmental factors [41]. In 1993, Rier et al. first reported that environmental toxins such as dioxins are potential risk factors for endometriosis [22]; subsequently, Koninckx et al., Mayani et al., and others have obtained similar results [19, 21]. As one of the links between environmental factors and genetic factors, toxin-metabolizing enzymes have crucial roles in the pathogenesis of endometriosis. With increasing research focused on polymorphisms in genes encoding toxin-metabolizing enzymes, more and more attention has been paid to the relationships between these enzymes and genetic susceptibility to endometriosis.

NAT2 is an important two-phase detoxifying enzyme for the in vivo transformation of exogenous chemicals and is involved in the metabolism of various environmental toxicants [21]. Twenty-seven alleles have been reported, and the C481T, G590A, A803G, and G857A SNPs cause decreased enzyme activity and impaired acetylation activity, resulting in differences in susceptibility to cancer, diabetes, and allergic diseases [12, 42]. Some researchers have speculated that changes in NAT2 activity increase the risk of endometriosis or that there is a link between an imbalance in *NAT2* gene polymorphisms and susceptibility to endometriosis. In 1999, Baranvo et al. reported that stage I–II endometriosis in the French population is associated with slow acetylation [27]. However, in 2001, Nakago et al. reported that *NAT2* polymorphisms have no relationship with the risk of endometriosis in the UK population [34]. In 2018, Fayez et al. found that patients with endometriosis in Iran are more likely to show the fast acetylation phenotype [14]. Thus, there is no consensus regarding whether the *NAT2* gene is associated with endometriosis.

Previously, Guo et al. reviewed the literature on this topic. This study comprised a meta-analysis of the NAT2 slow acetylation phenotype and endometriosis. No significant associations were found at that time [25]. Here, we included four additional new studies on this topic, including one study of Caucasians and three studies of Asians. We also performed subgroup analysis and added a meta-analysis between different SNPs and endometriosis risk. To our knowledge, this meta-analysis includes the largest sample size of studies on this topic and provides new insights on the clinical significance of NAT2.

We comprehensively evaluated NAT2 phenotypes with respect to the risk of endometriosis, including a subgroup analysis according to ethnic groups. We concluded that the NAT2 slow acetylation phenotype is a risk factor for endometriosis in the Asian population. We also found that there is no association between the slow, intermediate, or fast acetylation NAT2 phenotypes and endometriosis in Caucasian individuals. Furthermore, we provide additional evidence that the G590A SNP might act as a protective factor. Studies on this SNP exhibit low heterogeneity. No statistically significant results were found between rs1799931, rs1208, or rs1799929 and endometriosis. Sensitivity analyses were also consistent with the overall study results, establishing the stability and reliability of our results. From this analysis, we found that most of the heterogeneity is derived from Deguchi et al.’s article. After excluding this study, the heterogeneity was significantly reduced. Perhaps the choice of newborn children in Japan as controls could account for this heterogeneity. More elaborate studies are thus needed for the Japanese population.

It is worth mentioning that an increasing number of genome-wide association studies have identified polymorphisms associated with endometriosis including rs10859871, rs10965235, rs1270667, rs13394619, rs1537377, rs758316, rs7739264, rs7521902, and rs16826658. However, these studies have not conclusively supported an association between rs1799930 (G590A) or rs1799931 (G857A) and endometriosis [43–46]. Therefore, more elaborate hierarchical genome-wide association studies are needed to examine the relationships between these SNPs and endometriosis with respect to a variety of ethnicities and stages and to eliminate confounding factors like dioxin exposure, pollution exposure, lifestyle, diabetes, smoking, coffee intake, breast feeding time, or other genetic factors [47].

There was substantial heterogeneity among results with respect to the association between the NAT2 phenotype and endometriosis risk, and these differences might be related to race and geographical differences. *NAT2* genotype frequencies were significantly different among races and regions. More than 53% of Western Caucasians harbored the slow acetylation type, whereas the Eastern Asians predominantly had the fast acetylation type [36]. Because the pathogenesis of endometriosis is highly complex, the underlying genetic mechanism might differ among populations. These differences among studies suggest differences in the underlying genetic factors, further validating the genetic diversity of endometriosis. Based on a subgroup analysis, heterogeneity was effectively eliminated. Furthermore, endometriosis is a polygenic disease, involving many pathogenic genes and environmental factors. Various environmental conditions like smoking, drinking, age, economic status, medical history, dioxin exposure, pollution exposure, lifestyle, diabetes, coffee intake, breast feeding time, or other genetic factors should be considered and controlled for in both case and control groups. As one of the studies included in this meta-analysis, Matsuzaka et al. reported the smoking history and BMI distribution and Baranova et al. reported the distribution of clinical symptoms relative to NAT2 genotypes. Further, a recent study revealed that breast feeding time could significantly account for endometriosis risk. However, no studies have been performed adjusting for these factors, and thus, more elaborate studies are needed on this topic.

Some limitations of this study should be mentioned. First, it only considered certain databases and unpublished negative results or ongoing research might be missing, thereby affecting our results. Second, only data for Asian and Caucasian populations are reported in the databases, and gene frequency distributions for other ethnicities like African or Latin American populations are unavailable. Third, heterogeneity in the NAT2 phenotype between Asian and Caucasian individuals should not be ignored. When we performed subgroup analysis of different ethnicities, heterogeneity was effectively eliminated, but more precise studies of different regions or ethnicities should also be performed. Fourth, the heterogeneity among existing studies might be explained by sampling errors and the small number of samples in some studies. More samples and data are urgently needed, and analyses should be subdivided according to the stages of endometriosis. However, despite these limitations, based on an elaborately designed protocol and using comprehensive methods to evaluate previous studies, we conclude that there is an association between NAT2 polymorphisms and endometriosis. Our research suggests that there is still room for improvement with respect to studies on the genetic basis of endometriosis.

## Conclusion

In summary, the rs1799930 mutant genotypes are associated with a decreased risk of endometriosis. No statistically significant results were found between rs1799931, rs1208, or rs1799929 and endometriosis. In a subgroup analysis based on ethnicity, the NAT2 slow acetylation phenotype was found to increase the risk of endometriosis in Asians. However, no statistically significant results were found between the NAT2 slow acetylation phenotype and endometriosis risk in Caucasians. These SNPs and NAT2 phenotype are potential biomarkers for the diagnosis and treatment of endometriosis. Further large-scale case-control studies are needed, with specific designs to account for the disease stage, for a more in-depth and thorough exploration of the relationship between NAT2 polymorphisms and endometriosis.

## Supporting information

S1 File Excluded studies list. (DOCX)

S2 File Meta-analysis on genetic association studies checklist. (DOCX)

S3 File PRISMA checklist. (DOC)

S4 File PRISMA flow diagram. (DOC)

## Abbreviations

95% CI: 95% confidence interval
NAT2: N-acetyltransferase 2
CNKI: China National Knowledge Infrastructure
OR: odds ratio
CYP1A1: cytochrome P4501A1
CYP19A1: cytochrome P45019A1
COMT: catechol-O-methyl transferase
GSTP1: glutathione S-transferase P1
SNP: single-nucleotide polymorphism
NOS: Newcastle–Ottawa scale
BMI: body mass index
GWAS: genome-wide association studies
PCBs: polychlorinated biphenyls
