# Supplementary material for: NAT2 gene polymorphisms and endometriosis risk: A PRISMA-compliant meta-analysis
Source: PLoS One. 2019 Dec 27;14(12):e0227043. doi: 10.1371/journal.pone.0227043 (PMC6934289; doi:10.1371/journal.pone.0227043)
Supplement: S1 File — (DOCX) [file pone.0227043.s001.docx]

Excluded studies list

The electronic search retrieved 617articles. A total number of 604 articles were excluded based on their titles and abstracts. The remaining 13 articles were screened in full texts. One article was excluded for being review. Two articles were excluded for not reporting exact genotye gene. One is excluded for being single-arm study. Thus, a total number of 9 articles was retained for data extraction.

excluded based on their titles and abstracts:

1. Abe, G., Lee, S. H., Chang, M., Liu, S. C., Tsai, H. Y., & Ota, K. G. (2014). The origin of the bifurcated axial skeletal system in the twin-tail goldfish. Nat Commun, 5, 3360. doi:10.1038/ncomms4360
2. Abitbol, M., Menini, C., Delezoide, A. L., Rhyner, T., Vekemans, M., & Mallet, J. (1993). Nucleus basalis magnocellularis and hippocampus are the major sites of FMR-1 expression in the human fetal brain. Nat Genet, 4(2), 147-153. doi:10.1038/ng0693-147
3. Abrams, A. J., Hufnagel, R. B., Rebelo, A., Zanna, C., Patel, N., Gonzalez, M. A., . . . Dallman, J. E. (2015). Mutations in SLC25A46, encoding a UGO1-like protein, cause an optic atrophy spectrum disorder. Nat Genet, 47(8), 926-932. doi:10.1038/ng.3354
4. Adhikari, K., Reales, G., Smith, A. J., Konka, E., Palmen, J., Quinto-Sanchez, M., . . . Ruiz-Linares, A. (2015). A genome-wide association study identifies multiple loci for variation in human ear morphology. Nat Commun, 6, 7500. doi:10.1038/ncomms8500
5. Afzal, A. R., Rajab, A., Fenske, C. D., Oldridge, M., Elanko, N., Ternes-Pereira, E., . . . Jeffery, S. (2000). Recessive Robinow syndrome, allelic to dominant brachydactyly type B, is caused by mutation of ROR2. Nat Genet, 25(4), 419-422. doi:10.1038/78107
6. Akimaru, H., Hou, D. X., & Ishii, S. (1997). Drosophila CBP is required for dorsal-dependent twist gene expression. Nat Genet, 17(2), 211-214. doi:10.1038/ng1097-211
7. Akum, B. F., Chen, M., Gunderson, S. I., Riefler, G. M., Scerri-Hansen, M. M., & Firestein, B. L. (2004). Cypin regulates dendrite patterning in hippocampal neurons by promoting microtubule assembly. Nat Neurosci, 7(2), 145-152. doi:10.1038/nn1179
8. Albers, C. A., Paul, D. S., Schulze, H., Freson, K., Stephens, J. C., Smethurst, P. A., . . . Ghevaert, C. (2012). Compound inheritance of a low-frequency regulatory SNP and a rare null mutation in exon-junction complex subunit RBM8A causes TAR syndrome. Nat Genet, 44(4), 435-439, s431-432. doi:10.1038/ng.1083
9. Alfano, G., Vitiello, C., Caccioppoli, C., Caramico, T., Carola, A., Szego, M. J., . . . Banfi, S. (2005). Natural antisense transcripts associated with genes involved in eye development. Hum Mol Genet, 14(7), 913-923. doi:10.1093/hmg/ddi084
10. Alfred, J. (2000). Development biology. Nodal signalling gets foxy. Nat Rev Genet, 1(2), 87. doi:10.1038/35038519
11. Alfred, J. (2001). Gene expression. Escaping silence. Nat Rev Genet, 2(1), 9. doi:10.1038/35047509
12. Ali, S., & Nawaz, W. (2017). Optimisation of nutritional requirements for dopamine synthesis by calcium alginate-entrapped mutant strain of Aspergillus oryzae EMS-6. Nat Prod Res, 31(3), 281-288. doi:10.1080/14786419.2016.1233408
13. Aligianis, I. A., Johnson, C. A., Gissen, P., Chen, D., Hampshire, D., Hoffmann, K., . . . Maher, E. R. (2005). Mutations of the catalytic subunit of RAB3GAP cause Warburg Micro syndrome. Nat Genet, 37(3), 221-223. doi:10.1038/ng1517
14. Allen, H. L., Flanagan, S. E., Shaw-Smith, C., De Franco, E., Akerman, I., Caswell, R., . . . Ellard, S. (2011). GATA6 haploinsufficiency causes pancreatic agenesis in humans. Nat Genet, 44(1), 20-22. doi:10.1038/ng.1035
15. Amanuma, K., Takeda, H., Amanuma, H., & Aoki, Y. (2000). Transgenic zebrafish for detecting mutations caused by compounds in aquatic environments. Nat Biotechnol, 18(1), 62-65. doi:10.1038/71938
16. Amemiya, C. T. (1998). The zebrafish and haematopoietic justice. Nat Genet, 20(3), 222-223. doi:10.1038/3016
17. Anderson, D. J., Kaplan, D. I., Bell, K. M., Koutsis, K., Haynes, J. M., Mills, R. J., . . . Elliott, D. A. (2018). NKX2-5 regulates human cardiomyogenesis via a HEY2 dependent transcriptional network. Nat Commun, 9(1), 1373. doi:10.1038/s41467-018-03714-x
18. Andrews, S. V., Ellis, S. E., Bakulski, K. M., Sheppard, B., Croen, L. A., Hertz-Picciotto, I., . . . Fallin, M. D. (2017). Cross-tissue integration of genetic and epigenetic data offers insight into autism spectrum disorder. Nat Commun, 8(1), 1011. doi:10.1038/s41467-017-00868-y
19. Anson-Cartwright, L., Dawson, K., Holmyard, D., Fisher, S. J., Lazzarini, R. A., & Cross, J. C. (2000). The glial cells missing-1 protein is essential for branching morphogenesis in the chorioallantoic placenta. Nat Genet, 25(3), 311-314. doi:10.1038/77076
20. Aoki, Y., Niihori, T., Kawame, H., Kurosawa, K., Ohashi, H., Tanaka, Y., . . . Matsubara, Y. (2005). Germline mutations in HRAS proto-oncogene cause Costello syndrome. Nat Genet, 37(10), 1038-1040. doi:10.1038/ng1641
21. Aravamudan, B., Fergestad, T., Davis, W. S., Rodesch, C. K., & Broadie, K. (1999). Drosophila UNC-13 is essential for synaptic transmission. Nat Neurosci, 2(11), 965-971. doi:10.1038/14764
22. Aref-Eshghi, E., Bend, E. G., Hood, R. L., Schenkel, L. C., Carere, D. A., Chakrabarti, R., . . . Sadikovic, B. (2018). BAFopathies' DNA methylation epi-signatures demonstrate diagnostic utility and functional continuum of Coffin-Siris and Nicolaides-Baraitser syndromes. Nat Commun, 9(1), 4885. doi:10.1038/s41467-018-07193-y
23. Arikawa-Hirasawa, E., Watanabe, H., Takami, H., Hassell, J. R., & Yamada, Y. (1999). Perlecan is essential for cartilage and cephalic development. Nat Genet, 23(3), 354-358. doi:10.1038/15537
24. Asada, Y., Varnum, D. S., Frankel, W. N., & Nadeau, J. H. (1994). A mutation in the Ter gene causing increased susceptibility to testicular teratomas maps to mouse chromosome 18. Nat Genet, 6(4), 363-368. doi:10.1038/ng0494-363
25. Asaoka-Taguchi, M., Yamada, M., Nakamura, A., Hanyu, K., & Kobayashi, S. (1999). Maternal Pumilio acts together with Nanos in germline development in Drosophila embryos. Nat Cell Biol, 1(7), 431-437. doi:10.1038/15666
26. Asturias, F. J., Chadick, J. Z., Cheung, I. K., Stark, H., Witkowski, A., Joshi, A. K., & Smith, S. (2005). Structure and molecular organization of mammalian fatty acid synthase. Nat Struct Mol Biol, 12(3), 225-232. doi:10.1038/nsmb899
27. Aulehla, A., Wiegraebe, W., Baubet, V., Wahl, M. B., Deng, C., Taketo, M., . . . Pourquie, O. (2008). A beta-catenin gradient links the clock and wavefront systems in mouse embryo segmentation. Nat Cell Biol, 10(2), 186-193. doi:10.1038/ncb1679
28. Avery, A. W., Fealey, M. E., Wang, F., Orlova, A., Thompson, A. R., Thomas, D. D., . . . Egelman, E. H. (2017). Structural basis for high-affinity actin binding revealed by a beta-III-spectrin SCA5 missense mutation. Nat Commun, 8(1), 1350. doi:10.1038/s41467-017-01367-w
29. Azuma, N., Nishina, S., Yanagisawa, H., Okuyama, T., & Yamada, M. (1996). PAX6 missense mutation in isolated foveal hypoplasia. Nat Genet, 13(2), 141-142. doi:10.1038/ng0696-141
30. Bachner, D., Steinbach, P., Wohrle, D., Just, W., Vogel, W., Hameister, H., . . . Poustka, A. (1993). Enhanced Fmr-1 expression in testis. Nat Genet, 4(2), 115-116. doi:10.1038/ng0693-115
31. Badone, F. C., Cassani, E., Landoni, M., Doria, E., Panzeri, D., Lago, C., . . . Pilu, R. (2010). The low phytic acid1-241 (lpa1-241) maize mutation alters the accumulation of anthocyanin pigment in the kernel. Planta, 231(5), 1189-1199. doi:10.1007/s00425-010-1123-z
32. Bai, J., Ramos, R. L., Ackman, J. B., Thomas, A. M., Lee, R. V., & LoTurco, J. J. (2003). RNAi reveals doublecortin is required for radial migration in rat neocortex. Nat Neurosci, 6(12), 1277-1283. doi:10.1038/nn1153
33. Bai, R. Y., Koester, C., Ouyang, T., Hahn, S. A., Hammerschmidt, M., Peschel, C., & Duyster, J. (2002). SMIF, a Smad4-interacting protein that functions as a co-activator in TGFbeta signalling. Nat Cell Biol, 4(3), 181-190. doi:10.1038/ncb753
34. Bajjalieh, S. (2004). Trafficking in cell fate. Nat Genet, 36(3), 216-217. doi:10.1038/ng0304-216
35. Bamford, R. N., Roessler, E., Burdine, R. D., Saplakoglu, U., dela Cruz, J., Splitt, M., . . . Casey, B. (2000). Loss-of-function mutations in the EGF-CFC gene CFC1 are associated with human left-right laterality defects. Nat Genet, 26(3), 365-369. doi:10.1038/81695
36. Bamshad, M., Lin, R. C., Law, D. J., Watkins, W. C., Krakowiak, P. A., Moore, M. E., . . . Jorde, L. B. (1997). Mutations in human TBX3 alter limb, apocrine and genital development in ulnar-mammary syndrome. Nat Genet, 16(3), 311-315. doi:10.1038/ng0797-311
37. Barak, T., Kwan, K. Y., Louvi, A., Demirbilek, V., Saygi, S., Tuysuz, B., . . . Gunel, M. (2011). Recessive LAMC3 mutations cause malformations of occipital cortical development. Nat Genet, 43(6), 590-594. doi:10.1038/ng.836
38. Barban, N., Jansen, R., de Vlaming, R., Vaez, A., Mandemakers, J. J., Tropf, F. C., . . . Mills, M. C. (2016). Genome-wide analysis identifies 12 loci influencing human reproductive behavior. Nat Genet, 48(12), 1462-1472. doi:10.1038/ng.3698
39. Barbuta, R., Scripcaru, G., Harmanschi, A., Holban, L., & Grumazescu, C. (1971). [Partial deletion of the short arm of chromosome 5 and partial deletion of the short arm of a chromosome of the group C (6-12)]. Rev Med Chir Soc Med Nat Iasi, 75(1), 205-210.
40. Bardoni, B., Zanaria, E., Guioli, S., Floridia, G., Worley, K. C., Tonini, G., . . . et al. (1994). A dosage sensitive locus at chromosome Xp21 is involved in male to female sex reversal. Nat Genet, 7(4), 497-501. doi:10.1038/ng0894-497
41. Barila, D., & Superti-Furga, G. (1998). An intramolecular SH3-domain interaction regulates c-Abl activity. Nat Genet, 18(3), 280-282. doi:10.1038/ng0398-280
42. Barker, V., Middleton, G., Davey, F., & Davies, A. M. (2001). TNFalpha contributes to the death of NGF-dependent neurons during development. Nat Neurosci, 4(12), 1194-1198. doi:10.1038/nn755
43. Barna, M., Hawe, N., Niswander, L., & Pandolfi, P. P. (2000). Plzf regulates limb and axial skeletal patterning. Nat Genet, 25(2), 166-172. doi:10.1038/76014
44. Basson, C. T., Bachinsky, D. R., Lin, R. C., Levi, T., Elkins, J. A., Soults, J., . . . Seidman, C. E. (1997). Mutations in human TBX5 [corrected] cause limb and cardiac malformation in Holt-Oram syndrome. Nat Genet, 15(1), 30-35. doi:10.1038/ng0197-30
45. Bates, B., Rios, M., Trumpp, A., Chen, C., Fan, G., Bishop, J. M., & Jaenisch, R. (1999). Neurotrophin-3 is required for proper cerebellar development. Nat Neurosci, 2(2), 115-117. doi:10.1038/5669
46. Batourina, E., Gim, S., Bello, N., Shy, M., Clagett-Dame, M., Srinivas, S., . . . Mendelsohn, C. (2001). Vitamin A controls epithelial/mesenchymal interactions through Ret expression. Nat Genet, 27(1), 74-78. doi:10.1038/83792
47. Baxendale, S., Davison, C., Muxworthy, C., Wolff, C., Ingham, P. W., & Roy, S. (2004). The B-cell maturation factor Blimp-1 specifies vertebrate slow-twitch muscle fiber identity in response to Hedgehog signaling. Nat Genet, 36(1), 88-93. doi:10.1038/ng1280
48. Baxter, L. L., & Pavan, W. J. (2002). The oculocutaneous albinism type IV gene Matp is a new marker of pigment cell precursors during mouse embryonic development. Mech Dev, 116(1-2), 209-212.
49. Becam, I. E., Tanentzapf, G., Lepesant, J. A., Brown, N. H., & Huynh, J. R. (2005). Integrin-independent repression of cadherin transcription by talin during axis formation in Drosophila. Nat Cell Biol, 7(5), 510-516. doi:10.1038/ncb1253
50. Becker-Heck, A., Zohn, I. E., Okabe, N., Pollock, A., Lenhart, K. B., Sullivan-Brown, J., . . . Burdine, R. D. (2011). The coiled-coil domain containing protein CCDC40 is essential for motile cilia function and left-right axis formation. Nat Genet, 43(1), 79-84. doi:10.1038/ng.727
51. Behra, M., Cousin, X., Bertrand, C., Vonesch, J. L., Biellmann, D., Chatonnet, A., & Strahle, U. (2002). Acetylcholinesterase is required for neuronal and muscular development in the zebrafish embryo. Nat Neurosci, 5(2), 111-118. doi:10.1038/nn788
52. Bell, D. M., Leung, K. K., Wheatley, S. C., Ng, L. J., Zhou, S., Ling, K. W., . . . Cheah, K. S. (1997). SOX9 directly regulates the type-II collagen gene. Nat Genet, 16(2), 174-178. doi:10.1038/ng0697-174
53. Belousoff, M. J., Eyal, Z., Radjainia, M., Ahmed, T., Bamert, R. S., Matzov, D., . . . Yonath, A. (2017). Structural Basis for Linezolid Binding Site Rearrangement in the Staphylococcus aureus Ribosome. MBio, 8(3). doi:10.1128/mBio.00395-17
54. Benlekbir, S., Bueler, S. A., & Rubinstein, J. L. (2012). Structure of the vacuolar-type ATPase from Saccharomyces cerevisiae at 11-A resolution. Nat Struct Mol Biol, 19(12), 1356-1362. doi:10.1038/nsmb.2422
55. Bergstralh, D. T., Lovegrove, H. E., & St Johnston, D. (2015). Lateral adhesion drives reintegration of misplaced cells into epithelial monolayers. Nat Cell Biol, 17(11), 1497-1503. doi:10.1038/ncb3248
56. Bi, W., Deng, J. M., Zhang, Z., Behringer, R. R., & de Crombrugghe, B. (1999). Sox9 is required for cartilage formation. Nat Genet, 22(1), 85-89. doi:10.1038/8792
57. Bi, W., Sapir, T., Shchelochkov, O. A., Zhang, F., Withers, M. A., Hunter, J. V., . . . Reiner, O. (2009). Increased LIS1 expression affects human and mouse brain development. Nat Genet, 41(2), 168-177. doi:10.1038/ng.302
58. Bicknell, L. S., Bongers, E. M., Leitch, A., Brown, S., Schoots, J., Harley, M. E., . . . Jackson, A. P. (2011). Mutations in the pre-replication complex cause Meier-Gorlin syndrome. Nat Genet, 43(4), 356-359. doi:10.1038/ng.775
59. Bicknell, L. S., Walker, S., Klingseisen, A., Stiff, T., Leitch, A., Kerzendorfer, C., . . . Jeggo, P. A. (2011). Mutations in ORC1, encoding the largest subunit of the origin recognition complex, cause microcephalic primordial dwarfism resembling Meier-Gorlin syndrome. Nat Genet, 43(4), 350-355. doi:10.1038/ng.776
60. Bitner-Glindzicz, M., Lindley, K. J., Rutland, P., Blaydon, D., Smith, V. V., Milla, P. J., . . . Glaser, B. (2000). A recessive contiguous gene deletion causing infantile hyperinsulinism, enteropathy and deafness identifies the Usher type 1C gene. Nat Genet, 26(1), 56-60. doi:10.1038/79178
61. Bocking, T., Aguet, F., Harrison, S. C., & Kirchhausen, T. (2011). Single-molecule analysis of a molecular disassemblase reveals the mechanism of Hsc70-driven clathrin uncoating. Nat Struct Mol Biol, 18(3), 295-301. doi:10.1038/nsmb.1985
62. Bond, A. M., Vangompel, M. J., Sametsky, E. A., Clark, M. F., Savage, J. C., Disterhoft, J. F., & Kohtz, J. D. (2009). Balanced gene regulation by an embryonic brain ncRNA is critical for adult hippocampal GABA circuitry. Nat Neurosci, 12(8), 1020-1027. doi:10.1038/nn.2371
63. Boute, N., Gribouval, O., Roselli, S., Benessy, F., Lee, H., Fuchshuber, A., . . . Antignac, C. (2000). NPHS2, encoding the glomerular protein podocin, is mutated in autosomal recessive steroid-resistant nephrotic syndrome. Nat Genet, 24(4), 349-354. doi:10.1038/74166
64. Boyadjiev, S. A., Fromme, J. C., Ben, J., Chong, S. S., Nauta, C., Hur, D. J., . . . Eyaid, W. (2006). Cranio-lenticulo-sutural dysplasia is caused by a SEC23A mutation leading to abnormal endoplasmic-reticulum-to-Golgi trafficking. Nat Genet, 38(10), 1192-1197. doi:10.1038/ng1876
65. Brennan, J., & Capel, B. (2004). One tissue, two fates: molecular genetic events that underlie testis versus ovary development. Nat Rev Genet, 5(7), 509-521. doi:10.1038/nrg1381
66. Brent, A. E., & Tabin, C. J. (2004). White meat or dark? Nat Genet, 36(1), 8-10. doi:10.1038/ng0104-8
67. Brignole, E. J., Smith, S., & Asturias, F. J. (2009). Conformational flexibility of metazoan fatty acid synthase enables catalysis. Nat Struct Mol Biol, 16(2), 190-197. doi:10.1038/nsmb.1532
68. Brito, T. C., Possuelo, L. G., Valim, A. R. M., Todendi, P. F., Ribeiro, A. W., Gregianini, T. S., . . . Zaha, A. (2014). Polymorphisms in CYP2E1, GSTM1 and GSTT1 and anti-tuberculosis drug-induced hepatotoxicity. An Acad Bras Cienc, 86(2), 855-865.
69. Brockerhoff, S. E. (2006). Measuring the optokinetic response of zebrafish larvae. Nat Protoc, 1(5), 2448-2451. doi:10.1038/nprot.2006.255
70. Brodbeck, D., Amherd, R., Callaerts, P., Hintermann, E., Meyer, U. A., & Affolter, M. (1998). Molecular and biochemical characterization of the aaNAT1 (Dat) locus in Drosophila melanogaster: differential expression of two gene products. DNA Cell Biol, 17(7), 621-633. doi:10.1089/dna.1998.17.621
71. Brunelli, S., Faiella, A., Capra, V., Nigro, V., Simeone, A., Cama, A., & Boncinelli, E. (1996). Germline mutations in the homeobox gene EMX2 in patients with severe schizencephaly. Nat Genet, 12(1), 94-96. doi:10.1038/ng0196-94
72. Budde, B. S., Namavar, Y., Barth, P. G., Poll-The, B. T., Nurnberg, G., Becker, C., . . . Baas, F. (2008). tRNA splicing endonuclease mutations cause pontocerebellar hypoplasia. Nat Genet, 40(9), 1113-1118. doi:10.1038/ng.204
73. Buffin, E., Emre, D., & Karess, R. E. (2007). Flies without a spindle checkpoint. Nat Cell Biol, 9(5), 565-572. doi:10.1038/ncb1570
74. Bulman, M. P., Kusumi, K., Frayling, T. M., McKeown, C., Garrett, C., Lander, E. S., . . . Turnpenny, P. D. (2000). Mutations in the human delta homologue, DLL3, cause axial skeletal defects in spondylocostal dysostosis. Nat Genet, 24(4), 438-441. doi:10.1038/74307
75. Burcoveanu, E., Rugina, V., & Covic, M. (1981). [Somatic manifestations in gonosomal syndromes. I. Study of Turner's syndrome]. Rev Med Chir Soc Med Nat Iasi, 85(1), 117-121.
76. Burgess, D. J. (2013). Disease genetics: Double danger from mitochondrial mutations. Nat Rev Genet, 14(10), 678-679. doi:10.1038/nrg3581
77. Burke, B., & Stewart, C. L. (2013). The nuclear lamins: flexibility in function. Nat Rev Mol Cell Biol, 14(1), 13-24. doi:10.1038/nrm3488
78. Burkhart, J. G. (2000). Fishing for mutations. Nat Biotechnol, 18(1), 21-22. doi:10.1038/71869
79. Burmeister, M., Novak, J., Liang, M. Y., Basu, S., Ploder, L., Hawes, N. L., . . . McInnes, R. R. (1996). Ocular retardation mouse caused by Chx10 homeobox null allele: impaired retinal progenitor proliferation and bipolar cell differentiation. Nat Genet, 12(4), 376-384. doi:10.1038/ng0496-376
80. Burtey, S. (2008). 22q11.2 microdeletion syndrome is a common cause of renal tract malformations. Nat Clin Pract Nephrol, 4(8), E1. doi:10.1038/ncpneph0906
81. Butnariu, L., Rusu, C., Caba, L., Panzaru, M., Braha, E., Gramescu, M., . . . Gorduza, E. V. (2013). Genotype- phenotype correlation in trisomy X: a retrospective study of a selected group of 36 patients and review of literature. Rev Med Chir Soc Med Nat Iasi, 117(3), 714-721.
82. Caba, L., Rusu, C., Butnariu, L., Panzaru, M., Braha, E., Volosciuc, M., . . . Gorduza, E. V. (2013). Phenotypic variability in Patau syndrome. Rev Med Chir Soc Med Nat Iasi, 117(2), 321-327.
83. Caba, L., Rusu, C., Volosciuc, M., Butnariu, L., Braha, E., Gramescu, M., . . . Covic, M. (2009). [Idiopathic mental retardation--importance of clinical diagnostic scores for case selection]. Rev Med Chir Soc Med Nat Iasi, 113(2), 523-526.
84. Camper, S. A. (2004). Sox3 and sexual dysfunction: it's in the head. Nat Genet, 36(3), 217-219. doi:10.1038/ng0304-217
85. Cappello, S., Attardo, A., Wu, X., Iwasato, T., Itohara, S., Wilsch-Brauninger, M., . . . Gotz, M. (2006). The Rho-GTPase cdc42 regulates neural progenitor fate at the apical surface. Nat Neurosci, 9(9), 1099-1107. doi:10.1038/nn1744
86. Cartier, M., Breitman, M. L., & Tsui, L. C. (1992). A frameshift mutation in the gamma E-crystallin gene of the Elo mouse. Nat Genet, 2(1), 42-45. doi:10.1038/ng0992-42
87. Carvalho, R. F., Beutler, M., Marler, K. J., Knoll, B., Becker-Barroso, E., Heintzmann, R., . . . Drescher, U. (2006). Silencing of EphA3 through a cis interaction with ephrinA5. Nat Neurosci, 9(3), 322-330. doi:10.1038/nn1655
88. Casci, T. (2000). Evo-devo. A head with no torso. Nat Rev Genet, 1(1), 9. doi:10.1038/35049527
89. Caubit, X., Gubellini, P., Andrieux, J., Roubertoux, P. L., Metwaly, M., Jacq, B., . . . Fasano, L. (2016). TSHZ3 deletion causes an autism syndrome and defects in cortical projection neurons. Nat Genet, 48(11), 1359-1369. doi:10.1038/ng.3681
90. Cenci, G., Siriaco, G., Raffa, G. D., Kellum, R., & Gatti, M. (2003). The Drosophila HOAP protein is required for telomere capping. Nat Cell Biol, 5(1), 82-84. doi:10.1038/ncb902
91. Chan, J. A., Balasubramanian, S., Witt, R. M., Nazemi, K. J., Choi, Y., Pazyra-Murphy, M. F., . . . Segal, R. A. (2009). Proteoglycan interactions with Sonic Hedgehog specify mitogenic responses. Nat Neurosci, 12(4), 409-417. doi:10.1038/nn.2287
92. Chang, C. J., Chao, C. H., Xia, W., Yang, J. Y., Xiong, Y., Li, C. W., . . . Hung, M. C. (2011). p53 regulates epithelial-mesenchymal transition and stem cell properties through modulating miRNAs. Nat Cell Biol, 13(3), 317-323. doi:10.1038/ncb2173
93. Chapman, D. B., Shashi, V., & Kirse, D. J. (2009). Case report: aplasia of the lacrimal and major salivary glands (ALSG). Int J Pediatr Otorhinolaryngol, 73(6), 899-901. doi:10.1016/j.ijporl.2009.03.004
94. Chaverra-Rodriguez, D., Macias, V. M., Hughes, G. L., Pujhari, S., Suzuki, Y., Peterson, D. R., . . . Rasgon, J. L. (2018). Targeted delivery of CRISPR-Cas9 ribonucleoprotein into arthropod ovaries for heritable germline gene editing. Nat Commun, 9(1), 3008. doi:10.1038/s41467-018-05425-9
95. Chavez, S. L., Loewke, K. E., Han, J., Moussavi, F., Colls, P., Munne, S., . . . Reijo Pera, R. A. (2012). Dynamic blastomere behaviour reflects human embryo ploidy by the four-cell stage. Nat Commun, 3, 1251. doi:10.1038/ncomms2249
96. Chen, B., Bronson, R. T., Klaman, L. D., Hampton, T. G., Wang, J. F., Green, P. J., . . . Neel, B. G. (2000). Mice mutant for Egfr and Shp2 have defective cardiac semilunar valvulogenesis. Nat Genet, 24(3), 296-299. doi:10.1038/73528
97. Chen, D., Ahlford, A., Schnorrer, F., Kalchhauser, I., Fellner, M., Viragh, E., . . . Dickson, B. J. (2008). High-resolution, high-throughput SNP mapping in Drosophila melanogaster. Nat Methods, 5(4), 323-329. doi:10.1038/nmeth.1191
98. Chen, D., Li, Y. P., Yu, Y. X., Zhou, T., Liu, C., Fei, E. K., . . . Wang, G. H. (2018). Dendritic cell nuclear protein-1 regulates melatonin biosynthesis by binding to BMAL1 and inhibiting the transcription of N-acetyltransferase in C6 cells. Acta Pharmacol Sin, 39(4), 597-606. doi:10.1038/aps.2017.163
99. Chen, J. F., Zhang, Y., Wilde, J., Hansen, K. C., Lai, F., & Niswander, L. (2014). Microcephaly disease gene Wdr62 regulates mitotic progression of embryonic neural stem cells and brain size. Nat Commun, 5, 3885. doi:10.1038/ncomms4885
100. Chen, M., Kato, K., Kubo, Y., Tanaka, Y., Liu, Y., Long, F., . . . Yao, M. (2017). Structural basis for tRNA-dependent cysteine biosynthesis. Nat Commun, 8(1), 1521. doi:10.1038/s41467-017-01543-y
101. Chen, Z. F., Paquette, A. J., & Anderson, D. J. (1998). NRSF/REST is required in vivo for repression of multiple neuronal target genes during embryogenesis. Nat Genet, 20(2), 136-142. doi:10.1038/2431
102. Chen, Z. Y., Battinelli, E. M., Fielder, A., Bundey, S., Sims, K., Breakefield, X. O., & Craig, I. W. (1993). A mutation in the Norrie disease gene (NDP) associated with X-linked familial exudative vitreoretinopathy. Nat Genet, 5(2), 180-183. doi:10.1038/ng1093-180
103. Cheng, J., Yang, H., Fang, J., Ma, L., Gong, R., Wang, P., . . . Xu, Y. (2015). Molecular mechanism for USP7-mediated DNMT1 stabilization by acetylation. Nat Commun, 6, 7023. doi:10.1038/ncomms8023
104. Cheung, I., Schertzer, M., Rose, A., & Lansdorp, P. M. (2002). Disruption of dog-1 in Caenorhabditis elegans triggers deletions upstream of guanine-rich DNA. Nat Genet, 31(4), 405-409. doi:10.1038/ng928
105. Cheutin, T., & Cavalli, G. (2018). Loss of PRC1 induces higher-order opening of Hox loci independently of transcription during Drosophila embryogenesis. Nat Commun, 9(1), 3898. doi:10.1038/s41467-018-05945-4
106. Chiang, J. C., Jiang, J., Newburger, P. E., & Lawrence, J. B. (2018). Trisomy silencing by XIST normalizes Down syndrome cell pathogenesis demonstrated for hematopoietic defects in vitro. Nat Commun, 9(1), 5180. doi:10.1038/s41467-018-07630-y
107. Ching, Y. H., Ghosh, T. K., Cross, S. J., Packham, E. A., Honeyman, L., Loughna, S., . . . Brook, J. D. (2005). Mutation in myosin heavy chain 6 causes atrial septal defect. Nat Genet, 37(4), 423-428. doi:10.1038/ng1526
108. Chipail, A., Constantinescu, V., Haimovici, M., Scripcaru, G., & Harmanschi, A. (1969). [Morphological and cytogenetic correlations in some congenital malformations]. Rev Med Chir Soc Med Nat Iasi, 73(1), 27-34.
109. Clifton-Bligh, R. J., Wentworth, J. M., Heinz, P., Crisp, M. S., John, R., Lazarus, J. H., . . . Chatterjee, V. K. (1998). Mutation of the gene encoding human TTF-2 associated with thyroid agenesis, cleft palate and choanal atresia. Nat Genet, 19(4), 399-401. doi:10.1038/1294
110. Colmenares, C., Heilstedt, H. A., Shaffer, L. G., Schwartz, S., Berk, M., Murray, J. C., & Stavnezer, E. (2002). Loss of the SKI proto-oncogene in individuals affected with 1p36 deletion syndrome is predicted by strain-dependent defects in Ski-/- mice. Nat Genet, 30(1), 106-109. doi:10.1038/ng770
111. Colvin, J. S., Bohne, B. A., Harding, G. W., McEwen, D. G., & Ornitz, D. M. (1996). Skeletal overgrowth and deafness in mice lacking fibroblast growth factor receptor 3. Nat Genet, 12(4), 390-397. doi:10.1038/ng0496-390
112. Connor, F., Bertwistle, D., Mee, P. J., Ross, G. M., Swift, S., Grigorieva, E., . . . Ashworth, A. (1997). Tumorigenesis and a DNA repair defect in mice with a truncating Brca2 mutation. Nat Genet, 17(4), 423-430. doi:10.1038/ng1297-423
113. Cooks, T., Pateras, I. S., Jenkins, L. M., Patel, K. M., Robles, A. I., Morris, J., . . . Harris, C. C. (2018). Mutant p53 cancers reprogram macrophages to tumor supporting macrophages via exosomal miR-1246. Nat Commun, 9(1), 771. doi:10.1038/s41467-018-03224-w
114. Copp, A. J., Greene, N. D., & Murdoch, J. N. (2003). The genetic basis of mammalian neurulation. Nat Rev Genet, 4(10), 784-793. doi:10.1038/nrg1181
115. Crisponi, L., Deiana, M., Loi, A., Chiappe, F., Uda, M., Amati, P., . . . Pilia, G. (2001). The putative forkhead transcription factor FOXL2 is mutated in blepharophimosis/ptosis/epicanthus inversus syndrome. Nat Genet, 27(2), 159-166. doi:10.1038/84781
116. D'Adamo, P., Menegon, A., Lo Nigro, C., Grasso, M., Gulisano, M., Tamanini, F., . . . Toniolo, D. (1998). Mutations in GDI1 are responsible for X-linked non-specific mental retardation. Nat Genet, 19(2), 134-139. doi:10.1038/487
117. Dattani, M. T., Martinez-Barbera, J. P., Thomas, P. Q., Brickman, J. M., Gupta, R., Martensson, I. L., . . . Robinson, I. C. (1998). Mutations in the homeobox gene HESX1/Hesx1 associated with septo-optic dysplasia in human and mouse. Nat Genet, 19(2), 125-133. doi:10.1038/477
118. De Felice, M., Ovitt, C., Biffali, E., Rodriguez-Mallon, A., Arra, C., Anastassiadis, K., . . . Di Lauro, R. (1998). A mouse model for hereditary thyroid dysgenesis and cleft palate. Nat Genet, 19(4), 395-398. doi:10.1038/1289
119. de Nijs, L., Leon, C., Nguyen, L., Loturco, J. J., Delgado-Escueta, A. V., Grisar, T., & Lakaye, B. (2009). EFHC1 interacts with microtubules to regulate cell division and cortical development. Nat Neurosci, 12(10), 1266-1274. doi:10.1038/nn.2390
120. de Pontual, L., Yao, E., Callier, P., Faivre, L., Drouin, V., Cariou, S., . . . Amiel, J. (2011). Germline deletion of the miR-17 approximately 92 cluster causes skeletal and growth defects in humans. Nat Genet, 43(10), 1026-1030. doi:10.1038/ng.915
121. Dean, M. (1996). Polarity, proliferation and the hedgehog pathway. Nat Genet, 14(3), 245-247. doi:10.1038/ng1196-245
122. Degenhardt, K., Singh, M. K., Aghajanian, H., Massera, D., Wang, Q., Li, J., . . . Epstein, J. A. (2013). Semaphorin 3d signaling defects are associated with anomalous pulmonary venous connections. Nat Med, 19(6), 760-765. doi:10.1038/nm.3185
123. Degiacomi, M. T., Iacovache, I., Pernot, L., Chami, M., Kudryashev, M., Stahlberg, H., . . . Dal Peraro, M. (2013). Molecular assembly of the aerolysin pore reveals a swirling membrane-insertion mechanism. Nat Chem Biol, 9(10), 623-629. doi:10.1038/nchembio.1312
124. Delepine, M., Nicolino, M., Barrett, T., Golamaully, M., Lathrop, G. M., & Julier, C. (2000). EIF2AK3, encoding translation initiation factor 2-alpha kinase 3, is mutated in patients with Wolcott-Rallison syndrome. Nat Genet, 25(4), 406-409. doi:10.1038/78085
125. Dentice, M., Bandyopadhyay, A., Gereben, B., Callebaut, I., Christoffolete, M. A., Kim, B. W., . . . Bianco, A. C. (2005). The Hedgehog-inducible ubiquitin ligase subunit WSB-1 modulates thyroid hormone activation and PTHrP secretion in the developing growth plate. Nat Cell Biol, 7(7), 698-705. doi:10.1038/ncb1272
126. Deol, M. S., & Whitten, W. K. (1972). X-chromosome inactivation: does it occur at the same time in all cells of the embryo? Nat New Biol, 240(104), 277-279.
127. des Georges, A., Katsuki, M., Drummond, D. R., Osei, M., Cross, R. A., & Amos, L. A. (2008). Mal3, the Schizosaccharomyces pombe homolog of EB1, changes the microtubule lattice. Nat Struct Mol Biol, 15(10), 1102-1108. doi:10.1038/nsmb.1482
128. Devenport, D., & Fuchs, E. (2008). Planar polarization in embryonic epidermis orchestrates global asymmetric morphogenesis of hair follicles. Nat Cell Biol, 10(11), 1257-1268. doi:10.1038/ncb1784
129. Diaconescu, S., Paduraru, G., Vascu, A. M., & Burlea, M. (2011). [Pierre Marie-Sainton cleidocranial dysplasia]. Rev Med Chir Soc Med Nat Iasi, 115(2), 341-348.
130. Dibbens, L. M., Tarpey, P. S., Hynes, K., Bayly, M. A., Scheffer, I. E., Smith, R., . . . Gecz, J. (2008). X-linked protocadherin 19 mutations cause female-limited epilepsy and cognitive impairment. Nat Genet, 40(6), 776-781. doi:10.1038/ng.149
131. Difilippantonio, S., Celeste, A., Fernandez-Capetillo, O., Chen, H. T., Reina San Martin, B., Van Laethem, F., . . . Nussenzweig, A. (2005). Role of Nbs1 in the activation of the Atm kinase revealed in humanized mouse models. Nat Cell Biol, 7(7), 675-685. doi:10.1038/ncb1270
132. Dominguez, M., Ferres-Marco, D., Gutierrez-Avino, F. J., Speicher, S. A., & Beneyto, M. (2004). Growth and specification of the eye are controlled independently by Eyegone and Eyeless in Drosophila melanogaster. Nat Genet, 36(1), 31-39. doi:10.1038/ng1281
133. Doobin, D. J., Kemal, S., Dantas, T. J., & Vallee, R. B. (2016). Severe NDE1-mediated microcephaly results from neural progenitor cell cycle arrests at multiple specific stages. Nat Commun, 7, 12551. doi:10.1038/ncomms12551
134. Dorner, S., Brunelle, J. L., Sharma, D., & Green, R. (2006). The hybrid state of tRNA binding is an authentic translation elongation intermediate. Nat Struct Mol Biol, 13(3), 234-241. doi:10.1038/nsmb1060
135. Dreyer, S. D., Zhou, G., Baldini, A., Winterpacht, A., Zabel, B., Cole, W., . . . Lee, B. (1998). Mutations in LMX1B cause abnormal skeletal patterning and renal dysplasia in nail patella syndrome. Nat Genet, 19(1), 47-50. doi:10.1038/ng0598-47
136. Dubail, J., Huber, C., Chantepie, S., Sonntag, S., Tuysuz, B., Mihci, E., . . . Cormier-Daire, V. (2018). SLC10A7 mutations cause a skeletal dysplasia with amelogenesis imperfecta mediated by GAG biosynthesis defects. Nat Commun, 9(1), 3087. doi:10.1038/s41467-018-05191-8
137. Dumitru, R., Scarlat, A., lonescu, M., & Dumitrascu, T. (2012). Left-sided duplication of inferior vena cava: clinical implications in a patient with sigmoid adenocarcinoma. Rev Med Chir Soc Med Nat Iasi, 116(3), 858-861.
138. Eberhart, D. E., & Curran, T. (1998). Yayoi era mutation disrupts brain and muscle. Nat Med, 4(9), 1002-1003. doi:10.1038/1988
139. Ebersole, T. A., Chen, Q., Justice, M. J., & Artzt, K. (1996). The quaking gene product necessary in embryogenesis and myelination combines features of RNA binding and signal transduction proteins. Nat Genet, 12(3), 260-265. doi:10.1038/ng0396-260
140. Edgar, B. A. (1999). From small flies come big discoveries about size control. Nat Cell Biol, 1(8), E191-193. doi:10.1038/70217
141. Edlund, H. (2002). Pancreatic organogenesis--developmental mechanisms and implications for therapy. Nat Rev Genet, 3(7), 524-532. doi:10.1038/nrg841
142. Eichelbaum, M., & Evert, B. (1996). Influence of pharmacogenetics on drug disposition and response. Clin Exp Pharmacol Physiol, 23(10-11), 983-985.
143. Entesarian, M., Matsson, H., Klar, J., Bergendal, B., Olson, L., Arakaki, R., . . . Dahl, N. (2005). Mutations in the gene encoding fibroblast growth factor 10 are associated with aplasia of lacrimal and salivary glands. Nat Genet, 37(2), 125-127. doi:10.1038/ng1507
144. Esteve, P., Sandonis, A., Cardozo, M., Malapeira, J., Ibanez, C., Crespo, I., . . . Bovolenta, P. (2011). SFRPs act as negative modulators of ADAM10 to regulate retinal neurogenesis. Nat Neurosci, 14(5), 562-569. doi:10.1038/nn.2794
145. Ewart, A. K., Morris, C. A., Atkinson, D., Jin, W., Sternes, K., Spallone, P., . . . Keating, M. T. (1993). Hemizygosity at the elastin locus in a developmental disorder, Williams syndrome. Nat Genet, 5(1), 11-16. doi:10.1038/ng0993-11
146. Fahrner, M., Stadlbauer, M., Muik, M., Rathner, P., Stathopulos, P., Ikura, M., . . . Romanin, C. (2018). A dual mechanism promotes switching of the Stormorken STIM1 R304W mutant into the activated state. Nat Commun, 9(1), 825. doi:10.1038/s41467-018-03062-w
147. Fakis, G., Boukouvala, S., Kawamura, A., & Kennedy, S. (2007). Description of a novel polymorphic gene encoding for arylamine N-acetyltransferase in the rhesus macaque (Macaca mulatta), a model animal for endometriosis. Pharmacogenet Genomics, 17(3), 181-188. doi:10.1097/FPC.0b013e328011e3ad
148. Fallon, J. R. (2011). Calcium channels put synapses in their place. Nat Neurosci, 14(5), 536-538. doi:10.1038/nn.2822
149. Fan, Z., Yamaza, T., Lee, J. S., Yu, J., Wang, S., Fan, G., . . . Wang, C. Y. (2009). BCOR regulates mesenchymal stem cell function by epigenetic mechanisms. Nat Cell Biol, 11(8), 1002-1009. doi:10.1038/ncb1913
150. Favaro, R., Valotta, M., Ferri, A. L., Latorre, E., Mariani, J., Giachino, C., . . . Nicolis, S. K. (2009). Hippocampal development and neural stem cell maintenance require Sox2-dependent regulation of Shh. Nat Neurosci, 12(10), 1248-1256. doi:10.1038/nn.2397
151. Felsenfeld, A. L. (1996). Defining the boundaries of zebrafish developmental genetics. Nat Genet, 14(3), 258-263. doi:10.1038/ng1196-258
152. Feng, H., Zhong, W., Punkosdy, G., Gu, S., Zhou, L., Seabolt, E. K., & Kipreos, E. T. (1999). CUL-2 is required for the G1-to-S-phase transition and mitotic chromosome condensation in Caenorhabditis elegans. Nat Cell Biol, 1(8), 486-492. doi:10.1038/70272
153. Feng, Y., & Walsh, C. A. (2004). The many faces of filamin: a versatile molecular scaffold for cell motility and signalling. Nat Cell Biol, 6(11), 1034-1038. doi:10.1038/ncb1104-1034
154. Ferguson, M. W. (2000). A hole in the head. Nat Genet, 24(4), 330-331. doi:10.1038/74132
155. Ferland, R. J., Eyaid, W., Collura, R. V., Tully, L. D., Hill, R. S., Al-Nouri, D., . . . Walsh, C. A. (2004). Abnormal cerebellar development and axonal decussation due to mutations in AHI1 in Joubert syndrome. Nat Genet, 36(9), 1008-1013. doi:10.1038/ng1419
156. Fernandez, L. C., Torres, M., & Real, F. X. (2016). Somatic mosaicism: on the road to cancer. Nat Rev Cancer, 16(1), 43-55. doi:10.1038/nrc.2015.1
157. Firulli, A. B., McFadden, D. G., Lin, Q., Srivastava, D., & Olson, E. N. (1998). Heart and extra-embryonic mesodermal defects in mouse embryos lacking the bHLH transcription factor Hand1. Nat Genet, 18(3), 266-270. doi:10.1038/ng0398-266
158. Firulli, B. A., Krawchuk, D., Centonze, V. E., Vargesson, N., Virshup, D. M., Conway, S. J., . . . Firulli, A. B. (2005). Altered Twist1 and Hand2 dimerization is associated with Saethre-Chotzen syndrome and limb abnormalities. Nat Genet, 37(4), 373-381. doi:10.1038/ng1525
159. Fisher, S., & Halpern, M. E. (1999). Patterning the zebrafish axial skeleton requires early chordin function. Nat Genet, 23(4), 442-446. doi:10.1038/70557
160. Flockhart, D. A., Clauw, D. J., Sale, E. B., Hewett, J., & Woosley, R. L. (1994). Pharmacogenetic characteristics of the eosinophilia-myalgia syndrome. Clin Pharmacol Ther, 56(4), 398-405.
161. Foley, J. E., Maeder, M. L., Pearlberg, J., Joung, J. K., Peterson, R. T., & Yeh, J. R. (2009). Targeted mutagenesis in zebrafish using customized zinc-finger nucleases. Nat Protoc, 4(12), 1855-1867. doi:10.1038/nprot.2009.209
162. Forrest, D., Erway, L. C., Ng, L., Altschuler, R., & Curran, T. (1996). Thyroid hormone receptor beta is essential for development of auditory function. Nat Genet, 13(3), 354-357. doi:10.1038/ng0796-354
163. Frank, R. A., Komiyama, N. H., Ryan, T. J., Zhu, F., O'Dell, T. J., & Grant, S. G. (2016). NMDA receptors are selectively partitioned into complexes and supercomplexes during synapse maturation. Nat Commun, 7, 11264. doi:10.1038/ncomms11264
164. Fukami, M., Wada, Y., Miyabayashi, K., Nishino, I., Hasegawa, T., Nordenskjold, A., . . . Ogata, T. (2006). CXorf6 is a causative gene for hypospadias. Nat Genet, 38(12), 1369-1371. doi:10.1038/ng1900
165. Furrer, M. P., Kim, S., Wolf, B., & Chiba, A. (2003). Robo and Frazzled/DCC mediate dendritic guidance at the CNS midline. Nat Neurosci, 6(3), 223-230. doi:10.1038/nn1017
166. Galanos, P., Vougas, K., Walter, D., Polyzos, A., Maya-Mendoza, A., Haagensen, E. J., . . . Gorgoulis, V. G. (2016). Chronic p53-independent p21 expression causes genomic instability by deregulating replication licensing. Nat Cell Biol, 18(7), 777-789. doi:10.1038/ncb3378
167. Galli, M., Munoz, J., Portegijs, V., Boxem, M., Grill, S. W., Heck, A. J., & van den Heuvel, S. (2011). aPKC phosphorylates NuMA-related LIN-5 to position the mitotic spindle during asymmetric division. Nat Cell Biol, 13(9), 1132-1138. doi:10.1038/ncb2315
168. Galvin, K. M., Donovan, M. J., Lynch, C. A., Meyer, R. I., Paul, R. J., Lorenz, J. N., . . . Huszar, D. (2000). A role for smad6 in development and homeostasis of the cardiovascular system. Nat Genet, 24(2), 171-174. doi:10.1038/72835
169. Gao, M. J., Li, X., Huang, J., Gropp, G. M., Gjetvaj, B., Lindsay, D. L., . . . Hegedus, D. D. (2015). SCARECROW-LIKE15 interacts with HISTONE DEACETYLASE19 and is essential for repressing the seed maturation programme. Nat Commun, 6, 7243. doi:10.1038/ncomms8243
170. Garcia, S. M., Tabach, Y., Lourenco, G. F., Armakola, M., & Ruvkun, G. (2014). Identification of genes in toxicity pathways of trinucleotide-repeat RNA in C. elegans. Nat Struct Mol Biol, 21(8), 712-720. doi:10.1038/nsmb.2858
171. Garcia, V., Bres, C., Just, D., Fernandez, L., Tai, F. W., Mauxion, J. P., . . . Rothan, C. (2016). Rapid identification of causal mutations in tomato EMS populations via mapping-by-sequencing. Nat Protoc, 11(12), 2401-2418. doi:10.1038/nprot.2016.143
172. Garcia-Gonzalo, F. R., Corbit, K. C., Sirerol-Piquer, M. S., Ramaswami, G., Otto, E. A., Noriega, T. R., . . . Reiter, J. F. (2011). A transition zone complex regulates mammalian ciliogenesis and ciliary membrane composition. Nat Genet, 43(8), 776-784. doi:10.1038/ng.891
173. Garcia-Higuera, I., Manchado, E., Dubus, P., Canamero, M., Mendez, J., Moreno, S., & Malumbres, M. (2008). Genomic stability and tumour suppression by the APC/C cofactor Cdh1. Nat Cell Biol, 10(7), 802-811. doi:10.1038/ncb1742
174. Gareus, R., Huth, M., Breiden, B., Nenci, A., Rosch, N., Haase, I., . . . Pasparakis, M. (2007). Normal epidermal differentiation but impaired skin-barrier formation upon keratinocyte-restricted IKK1 ablation. Nat Cell Biol, 9(4), 461-469. doi:10.1038/ncb1560
175. Ge, S., Xia, X., Ding, C., Zhen, B., Zhou, Q., Feng, J., . . . Qin, J. (2018). A proteomic landscape of diffuse-type gastric cancer. Nat Commun, 9(1), 1012. doi:10.1038/s41467-018-03121-2
176. Geissler, W. M., Davis, D. L., Wu, L., Bradshaw, K. D., Patel, S., Mendonca, B. B., . . . Andersson, S. (1994). Male pseudohermaphroditism caused by mutations of testicular 17 beta-hydroxysteroid dehydrogenase 3. Nat Genet, 7(1), 34-39. doi:10.1038/ng0594-34
177. Geng, X., Shi, Y., Nakagawa, A., Yoshina, S., Mitani, S., Shi, Y., & Xue, D. (2008). Inhibition of CED-3 zymogen activation and apoptosis in Caenorhabditis elegans by caspase homolog CSP-3. Nat Struct Mol Biol, 15(10), 1094-1101. doi:10.1038/nsmb.1488
178. Gerri, C., Marin-Juez, R., Marass, M., Marks, A., Maischein, H. M., & Stainier, D. Y. R. (2017). Hif-1alpha regulates macrophage-endothelial interactions during blood vessel development in zebrafish. Nat Commun, 8, 15492. doi:10.1038/ncomms15492
179. Ghiasvand, N. M., Rudolph, D. D., Mashayekhi, M., Brzezinski, J. A. t., Goldman, D., & Glaser, T. (2011). Deletion of a remote enhancer near ATOH7 disrupts retinal neurogenesis, causing NCRNA disease. Nat Neurosci, 14(5), 578-586. doi:10.1038/nn.2798
180. Ghosal, D., Trambaiolo, D., Amos, L. A., & Lowe, J. (2014). MinCD cell division proteins form alternating copolymeric cytomotive filaments. Nat Commun, 5, 5341. doi:10.1038/ncomms6341
181. Giera, S., Deng, Y., Luo, R., Ackerman, S. D., Mogha, A., Monk, K. R., . . . Piao, X. (2015). The adhesion G protein-coupled receptor GPR56 is a cell-autonomous regulator of oligodendrocyte development. Nat Commun, 6, 6121. doi:10.1038/ncomms7121
182. Gitler, A. D., Zhu, Y., Ismat, F. A., Lu, M. M., Yamauchi, Y., Parada, L. F., & Epstein, J. A. (2003). Nf1 has an essential role in endothelial cells. Nat Genet, 33(1), 75-79. doi:10.1038/ng1059
183. Glaser, T., Jepeal, L., Edwards, J. G., Young, S. R., Favor, J., & Maas, R. L. (1994). PAX6 gene dosage effect in a family with congenital cataracts, aniridia, anophthalmia and central nervous system defects. Nat Genet, 7(4), 463-471. doi:10.1038/ng0894-463
184. Glover, T. W. (1995). CATCHing a break on 22. Nat Genet, 10(3), 257-258. doi:10.1038/ng0795-257
185. Godenschwege, T. A., Hu, H., Shan-Crofts, X., Goodman, C. S., & Murphey, R. K. (2002). Bi-directional signaling by Semaphorin 1a during central synapse formation in Drosophila. Nat Neurosci, 5(12), 1294-1301. doi:10.1038/nn976
186. Gogendeau, D., Siudeja, K., Gambarotto, D., Pennetier, C., Bardin, A. J., & Basto, R. (2015). Aneuploidy causes premature differentiation of neural and intestinal stem cells. Nat Commun, 6, 8894. doi:10.1038/ncomms9894
187. Golding, J. P., Trainor, P., Krumlauf, R., & Gassmann, M. (2000). Defects in pathfinding by cranial neural crest cells in mice lacking the neuregulin receptor ErbB4. Nat Cell Biol, 2(2), 103-109. doi:10.1038/35000058
188. Golling, G., Amsterdam, A., Sun, Z., Antonelli, M., Maldonado, E., Chen, W., . . . Hopkins, N. (2002). Insertional mutagenesis in zebrafish rapidly identifies genes essential for early vertebrate development. Nat Genet, 31(2), 135-140. doi:10.1038/ng896
189. Gong, Y., Krakow, D., Marcelino, J., Wilkin, D., Chitayat, D., Babul-Hirji, R., . . . Warman, M. L. (1999). Heterozygous mutations in the gene encoding noggin affect human joint morphogenesis. Nat Genet, 21(3), 302-304. doi:10.1038/6821
190. Gordon, C. T., & Lyonnet, S. (2014). Enhancer mutations and phenotype modularity. Nat Genet, 46(1), 3-4. doi:10.1038/ng.2861
191. Gordon, C. T., Xue, S., Yigit, G., Filali, H., Chen, K., Rosin, N., . . . Reversade, B. (2017). De novo mutations in SMCHD1 cause Bosma arhinia microphthalmia syndrome and abrogate nasal development. Nat Genet, 49(2), 249-255. doi:10.1038/ng.3765
192. Gothilf, Y., Coon, S. L., Toyama, R., Chitnis, A., Namboodiri, M. A., & Klein, D. C. (1999). Zebrafish serotonin N-acetyltransferase-2: marker for development of pineal photoreceptors and circadian clock function. Endocrinology, 140(10), 4895-4903. doi:10.1210/endo.140.10.6975
193. Gothilf, Y., Toyama, R., Coon, S. L., Du, S. J., Dawid, I. B., & Klein, D. C. (2002). Pineal-specific expression of green fluorescent protein under the control of the serotonin-N-acetyltransferase gene regulatory regions in transgenic zebrafish. Dev Dyn, 225(3), 241-249. doi:10.1002/dvdy.10152
194. Gowen, L. C., Johnson, B. L., Latour, A. M., Sulik, K. K., & Koller, B. H. (1996). Brca1 deficiency results in early embryonic lethality characterized by neuroepithelial abnormalities. Nat Genet, 12(2), 191-194. doi:10.1038/ng0296-191
195. Graw, J. (2003). The genetic and molecular basis of congenital eye defects. Nat Rev Genet, 4(11), 876-888. doi:10.1038/nrg1202
196. Greaves, M. F., & Wiemels, J. (2003). Origins of chromosome translocations in childhood leukaemia. Nat Rev Cancer, 3(9), 639-649. doi:10.1038/nrc1164
197. Greaves, S. (2002). Polar exploration. Nat Cell Biol, 4(11), E256. doi:10.1038/ncb1102-e256
198. Gribouval, O., Gonzales, M., Neuhaus, T., Aziza, J., Bieth, E., Laurent, N., . . . Gubler, M. C. (2005). Mutations in genes in the renin-angiotensin system are associated with autosomal recessive renal tubular dysgenesis. Nat Genet, 37(9), 964-968. doi:10.1038/ng1623
199. Griffin, K. J., & Kimelman, D. (2002). One-Eyed Pinhead and Spadetail are essential for heart and somite formation. Nat Cell Biol, 4(10), 821-825. doi:10.1038/ncb862
200. Grinberg, I., Northrup, H., Ardinger, H., Prasad, C., Dobyns, W. B., & Millen, K. J. (2004). Heterozygous deletion of the linked genes ZIC1 and ZIC4 is involved in Dandy-Walker malformation. Nat Genet, 36(10), 1053-1055. doi:10.1038/ng1420
201. Gripp, K. W., Wotton, D., Edwards, M. C., Roessler, E., Ades, L., Meinecke, P., . . . Elledge, S. J. (2000). Mutations in TGIF cause holoprosencephaly and link NODAL signalling to human neural axis determination. Nat Genet, 25(2), 205-208. doi:10.1038/76074
202. Guenther, E. L., Ge, P., Trinh, H., Sawaya, M. R., Cascio, D., Boyer, D. R., . . . Eisenberg, D. S. (2018). Atomic-level evidence for packing and positional amyloid polymorphism by segment from TDP-43 RRM2. Nat Struct Mol Biol, 25(4), 311-319. doi:10.1038/s41594-018-0045-5
203. Guernsey, D. L., Matsuoka, M., Jiang, H., Evans, S., Macgillivray, C., Nightingale, M., . . . Samuels, M. E. (2011). Mutations in origin recognition complex gene ORC4 cause Meier-Gorlin syndrome. Nat Genet, 43(4), 360-364. doi:10.1038/ng.777
204. Guillemin, K., Williams, T., & Krasnow, M. A. (2001). A nuclear lamin is required for cytoplasmic organization and egg polarity in Drosophila. Nat Cell Biol, 3(9), 848-851. doi:10.1038/ncb0901-848
205. Guimier, A., Gabriel, G. C., Bajolle, F., Tsang, M., Liu, H., Noll, A., . . . Gordon, C. T. (2015). MMP21 is mutated in human heterotaxy and is required for normal left-right asymmetry in vertebrates. Nat Genet, 47(11), 1260-1263. doi:10.1038/ng.3376
206. Guirao, B., Meunier, A., Mortaud, S., Aguilar, A., Corsi, J. M., Strehl, L., . . . Spassky, N. (2010). Coupling between hydrodynamic forces and planar cell polarity orients mammalian motile cilia. Nat Cell Biol, 12(4), 341-350. doi:10.1038/ncb2040
207. Guris, D. L., Fantes, J., Tara, D., Druker, B. J., & Imamoto, A. (2001). Mice lacking the homologue of the human 22q11.2 gene CRKL phenocopy neurocristopathies of DiGeorge syndrome. Nat Genet, 27(3), 293-298. doi:10.1038/85855
208. Halford, M. M., Armes, J., Buchert, M., Meskenaite, V., Grail, D., Hibbs, M. L., . . . Stacker, S. A. (2000). Ryk-deficient mice exhibit craniofacial defects associated with perturbed Eph receptor crosstalk. Nat Genet, 25(4), 414-418. doi:10.1038/78099
209. Hallam, S. J., Goncharov, A., McEwen, J., Baran, R., & Jin, Y. (2002). SYD-1, a presynaptic protein with PDZ, C2 and rhoGAP-like domains, specifies axon identity in C. elegans. Nat Neurosci, 5(11), 1137-1146. doi:10.1038/nn959
210. Hamada, F., & Bienz, M. (2002). A Drosophila APC tumour suppressor homologue functions in cellular adhesion. Nat Cell Biol, 4(3), 208-213. doi:10.1038/ncb755
211. Hamada, H., Meno, C., Watanabe, D., & Saijoh, Y. (2002). Establishment of vertebrate left-right asymmetry. Nat Rev Genet, 3(2), 103-113. doi:10.1038/nrg732
212. Han, Y. C., Vidigal, J. A., Mu, P., Yao, E., Singh, I., Gonzalez, A. J., . . . Ventura, A. (2015). An allelic series of miR-17 approximately 92-mutant mice uncovers functional specialization and cooperation among members of a microRNA polycistron. Nat Genet, 47(7), 766-775. doi:10.1038/ng.3321
213. Hannon, E., Spiers, H., Viana, J., Pidsley, R., Burrage, J., Murphy, T. M., . . . Mill, J. (2016). Methylation QTLs in the developing brain and their enrichment in schizophrenia risk loci. Nat Neurosci, 19(1), 48-54. doi:10.1038/nn.4182
214. Hanson, I. M., Fletcher, J. M., Jordan, T., Brown, A., Taylor, D., Adams, R. J., . . . van Heyningen, V. (1994). Mutations at the PAX6 locus are found in heterogeneous anterior segment malformations including Peters' anomaly. Nat Genet, 6(2), 168-173. doi:10.1038/ng0294-168
215. Harrison, K. A., Thaler, J., Pfaff, S. L., Gu, H., & Kehrl, J. H. (1999). Pancreas dorsal lobe agenesis and abnormal islets of Langerhans in Hlxb9-deficient mice. Nat Genet, 23(1), 71-75. doi:10.1038/12674
216. Harry, J. L., Koopman, P., Brennan, F. E., Graves, J. A., & Renfree, M. B. (1995). Widespread expression of the testis-determining gene SRY in a marsupial. Nat Genet, 11(3), 347-349. doi:10.1038/ng1195-347
217. Harvey, N. L., Srinivasan, R. S., Dillard, M. E., Johnson, N. C., Witte, M. H., Boyd, K., . . . Oliver, G. (2005). Lymphatic vascular defects promoted by Prox1 haploinsufficiency cause adult-onset obesity. Nat Genet, 37(10), 1072-1081. doi:10.1038/ng1642
218. Hayes, S. (2006). Mesodermal Wnt expression promotes liver specification. Nat Cell Biol, 8(8), 789. doi:10.1038/ncb0806-789
219. Helwig, U., Imai, K., Schmahl, W., Thomas, B. E., Varnum, D. S., Nadeau, J. H., & Balling, R. (1995). Interaction between undulated and Patch leads to an extreme form of spina bifida in double-mutant mice. Nat Genet, 11(1), 60-63. doi:10.1038/ng0995-60
220. Hilton, M. J., Tu, X., Wu, X., Bai, S., Zhao, H., Kobayashi, T., . . . Long, F. (2008). Notch signaling maintains bone marrow mesenchymal progenitors by suppressing osteoblast differentiation. Nat Med, 14(3), 306-314. doi:10.1038/nm1716
221. Hing, A. V., Helms, C., Slaugh, R., Burgess, A., Wang, J. C., Herman, T., . . . Donis-Keller, H. (1995). Linkage of preaxial polydactyly type 2 to 7q36. Am J Med Genet, 58(2), 128-135. doi:10.1002/ajmg.1320580208
222. Hirotsune, S., Fleck, M. W., Gambello, M. J., Bix, G. J., Chen, A., Clark, G. D., . . . Wynshaw-Boris, A. (1998). Graded reduction of Pafah1b1 (Lis1) activity results in neuronal migration defects and early embryonic lethality. Nat Genet, 19(4), 333-339. doi:10.1038/1221
223. Hoff, S., Halbritter, J., Epting, D., Frank, V., Nguyen, T. M., van Reeuwijk, J., . . . Lienkamp, S. S. (2013). ANKS6 is a central component of a nephronophthisis module linking NEK8 to INVS and NPHP3. Nat Genet, 45(8), 951-956. doi:10.1038/ng.2681
224. Hogan, B. M., Bos, F. L., Bussmann, J., Witte, M., Chi, N. C., Duckers, H. J., & Schulte-Merker, S. (2009). Ccbe1 is required for embryonic lymphangiogenesis and venous sprouting. Nat Genet, 41(4), 396-398. doi:10.1038/ng.321
225. Hoischen, A., van Bon, B. W., Gilissen, C., Arts, P., van Lier, B., Steehouwer, M., . . . Veltman, J. A. (2010). De novo mutations of SETBP1 cause Schinzel-Giedion syndrome. Nat Genet, 42(6), 483-485. doi:10.1038/ng.581
226. Hoischen, A., van Bon, B. W., Rodriguez-Santiago, B., Gilissen, C., Vissers, L. E., de Vries, P., . . . de Vries, B. B. (2011). De novo nonsense mutations in ASXL1 cause Bohring-Opitz syndrome. Nat Genet, 43(8), 729-731. doi:10.1038/ng.868
227. Hong, S. E., Shugart, Y. Y., Huang, D. T., Shahwan, S. A., Grant, P. E., Hourihane, J. O., . . . Walsh, C. A. (2000). Autosomal recessive lissencephaly with cerebellar hypoplasia is associated with human RELN mutations. Nat Genet, 26(1), 93-96. doi:10.1038/79246
228. Hoogenraad, C. C., Koekkoek, B., Akhmanova, A., Krugers, H., Dortland, B., Miedema, M., . . . Galjart, N. (2002). Targeted mutation of Cyln2 in the Williams syndrome critical region links CLIP-115 haploinsufficiency to neurodevelopmental abnormalities in mice. Nat Genet, 32(1), 116-127. doi:10.1038/ng954
229. Horstick, E. J., Linsley, J. W., Dowling, J. J., Hauser, M. A., McDonald, K. K., Ashley-Koch, A., . . . Kuwada, J. Y. (2013). Stac3 is a component of the excitation-contraction coupling machinery and mutated in Native American myopathy. Nat Commun, 4, 1952. doi:10.1038/ncomms2952
230. Horwich, A., & Brueckner, M. (1993). Left, right and without a cue. Nat Genet, 5(4), 321-322. doi:10.1038/ng1293-321
231. Howard, H. C., Mount, D. B., Rochefort, D., Byun, N., Dupre, N., Lu, J., . . . Rouleau, G. A. (2002). The K-Cl cotransporter KCC3 is mutant in a severe peripheral neuropathy associated with agenesis of the corpus callosum. Nat Genet, 32(3), 384-392. doi:10.1038/ng1002
232. Hrabe de Angelis, M. H., Flaswinkel, H., Fuchs, H., Rathkolb, B., Soewarto, D., Marschall, S., . . . Balling, R. (2000). Genome-wide, large-scale production of mutant mice by ENU mutagenesis. Nat Genet, 25(4), 444-447. doi:10.1038/78146
233. Hu, Z., Li, Z., Yu, J., Tong, C., Lin, Y., Guo, X., . . . Sha, J. (2014). Association analysis identifies new risk loci for non-obstructive azoospermia in Chinese men. Nat Commun, 5, 3857. doi:10.1038/ncomms4857
234. Huai, C., Li, G., Yao, R., Zhang, Y., Cao, M., Kong, L., . . . Huang, Q. (2017). Structural insights into DNA cleavage activation of CRISPR-Cas9 system. Nat Commun, 8(1), 1375. doi:10.1038/s41467-017-01496-2
235. Hui, C. C., & Joyner, A. L. (1993). A mouse model of greig cephalopolysyndactyly syndrome: the extra-toesJ mutation contains an intragenic deletion of the Gli3 gene. Nat Genet, 3(3), 241-246. doi:10.1038/ng0393-241
236. Iadanza, M. G., Silvers, R., Boardman, J., Smith, H. I., Karamanos, T. K., Debelouchina, G. T., . . . Radford, S. E. (2018). The structure of a beta2-microglobulin fibril suggests a molecular basis for its amyloid polymorphism. Nat Commun, 9(1), 4517. doi:10.1038/s41467-018-06761-6
237. Iglesias, A. I., Mishra, A., Vitart, V., Bykhovskaya, Y., Hohn, R., Springelkamp, H., . . . MacGregor, S. (2018). Cross-ancestry genome-wide association analysis of corneal thickness strengthens link between complex and Mendelian eye diseases. Nat Commun, 9(1), 1864. doi:10.1038/s41467-018-03646-6
238. Imbeaud, S., Faure, E., Lamarre, I., Mattei, M. G., di Clemente, N., Tizard, R., . . . Picard, J. Y. (1995). Insensitivity to anti-mullerian hormone due to a mutation in the human anti-mullerian hormone receptor. Nat Genet, 11(4), 382-388. doi:10.1038/ng1295-382
239. Inagaki, N., Chihara, K., Arimura, N., Menager, C., Kawano, Y., Matsuo, N., . . . Kaibuchi, K. (2001). CRMP-2 induces axons in cultured hippocampal neurons. Nat Neurosci, 4(8), 781-782. doi:10.1038/90476
240. Iovine, M. K. (2007). Conserved mechanisms regulate outgrowth in zebrafish fins. Nat Chem Biol, 3(10), 613-618. doi:10.1038/nchembio.2007.36
241. Isidor, B., Cormier-Daire, V., Le Merrer, M., Lefrancois, T., Hamel, A., Le Caignec, C., . . . Jacquemont, S. (2008). Autosomal dominant spondylocarpotarsal synostosis syndrome: phenotypic homogeneity and genetic heterogeneity. Am J Med Genet A, 146a(12), 1593-1597. doi:10.1002/ajmg.a.32217
242. Iwasaki, M., Le, A. X., & Helms, J. A. (1997). Expression of indian hedgehog, bone morphogenetic protein 6 and gli during skeletal morphogenesis. Mech Dev, 69(1-2), 197-202.
243. Jacks, T., Shih, T. S., Schmitt, E. M., Bronson, R. T., Bernards, A., & Weinberg, R. A. (1994). Tumour predisposition in mice heterozygous for a targeted mutation in Nf1. Nat Genet, 7(3), 353-361. doi:10.1038/ng0794-353
244. Jackson, I. J. (2004). The G-netics of dark skin. Nat Genet, 36(9), 935-936. doi:10.1038/ng0904-935
245. Jacoby, M., Cox, J. J., Gayral, S., Hampshire, D. J., Ayub, M., Blockmans, M., . . . Schurmans, S. (2009). INPP5E mutations cause primary cilium signaling defects, ciliary instability and ciliopathies in human and mouse. Nat Genet, 41(9), 1027-1031. doi:10.1038/ng.427
246. Jadeja, S., Smyth, I., Pitera, J. E., Taylor, M. S., van Haelst, M., Bentley, E., . . . Scambler, P. J. (2005). Identification of a new gene mutated in Fraser syndrome and mouse myelencephalic blebs. Nat Genet, 37(5), 520-525. doi:10.1038/ng1549
247. Jagadeesan, A., Gunnarsdottir, E. D., Ebenesersdottir, S. S., Guethmundsdottir, V. B., Thordardottir, E. L., Einarsdottir, M. S., . . . Helgason, A. (2018). Reconstructing an African haploid genome from the 18th century. Nat Genet, 50(2), 199-205. doi:10.1038/s41588-017-0031-6
248. Jaglin, X. H., Poirier, K., Saillour, Y., Buhler, E., Tian, G., Bahi-Buisson, N., . . . Chelly, J. (2009). Mutations in the beta-tubulin gene TUBB2B result in asymmetrical polymicrogyria. Nat Genet, 41(6), 746-752. doi:10.1038/ng.380
249. Jamin, S. P., Arango, N. A., Mishina, Y., Hanks, M. C., & Behringer, R. R. (2002). Requirement of Bmpr1a for Mullerian duct regression during male sexual development. Nat Genet, 32(3), 408-410. doi:10.1038/ng1003
250. Jamuar, S. S., Schmitz-Abe, K., D'Gama, A. M., Drottar, M., Chan, W. M., Peeva, M., . . . Yu, T. W. (2017). Biallelic mutations in human DCC cause developmental split-brain syndrome. Nat Genet, 49(4), 606-612. doi:10.1038/ng.3804
251. Jenny, A., Reynolds-Kenneally, J., Das, G., Burnett, M., & Mlodzik, M. (2005). Diego and Prickle regulate Frizzled planar cell polarity signalling by competing for Dishevelled binding. Nat Cell Biol, 7(7), 691-697. doi:10.1038/ncb1271
252. Jerome, L. A., & Papaioannou, V. E. (2001). DiGeorge syndrome phenotype in mice mutant for the T-box gene, Tbx1. Nat Genet, 27(3), 286-291. doi:10.1038/85845
253. Jessen, J. R., Topczewski, J., Bingham, S., Sepich, D. S., Marlow, F., Chandrasekhar, A., & Solnica-Krezel, L. (2002). Zebrafish trilobite identifies new roles for Strabismus in gastrulation and neuronal movements. Nat Cell Biol, 4(8), 610-615. doi:10.1038/ncb828
254. Jiang, H., Ren, Y., Yuen, E. Y., Zhong, P., Ghaedi, M., Hu, Z., . . . Feng, J. (2012). Parkin controls dopamine utilization in human midbrain dopaminergic neurons derived from induced pluripotent stem cells. Nat Commun, 3, 668. doi:10.1038/ncomms1669
255. Jin, Z. B., Huang, X. F., Lv, J. N., Xiang, L., Li, D. Q., Chen, J., . . . Qu, J. (2014). SLC7A14 linked to autosomal recessive retinitis pigmentosa. Nat Commun, 5, 3517. doi:10.1038/ncomms4517
256. Jouet, M., Rosenthal, A., Armstrong, G., MacFarlane, J., Stevenson, R., Paterson, J., . . . Kenwrick, S. (1994). X-linked spastic paraplegia (SPG1), MASA syndrome and X-linked hydrocephalus result from mutations in the L1 gene. Nat Genet, 7(3), 402-407. doi:10.1038/ng0794-402
257. Juan, T., Geminard, C., Coutelis, J. B., Cerezo, D., Poles, S., Noselli, S., & Furthauer, M. (2018). Myosin1D is an evolutionarily conserved regulator of animal left-right asymmetry. Nat Commun, 9(1), 1942. doi:10.1038/s41467-018-04284-8
258. Kakkoura, M. G., Loizidou, M. A., Demetriou, C. A., Loucaides, G., Daniel, M., Kyriacou, K., & Hadjisavvas, A. (2017). The synergistic effect between the Mediterranean diet and GSTP1 or NAT2 SNPs decreases breast cancer risk in Greek-Cypriot women. Eur J Nutr, 56(2), 545-555. doi:10.1007/s00394-015-1099-3
259. Kaler, S. G., Gallo, L. K., Proud, V. K., Percy, A. K., Mark, Y., Segal, N. A., . . . Gahl, W. A. (1994). Occipital horn syndrome and a mild Menkes phenotype associated with splice site mutations at the MNK locus. Nat Genet, 8(2), 195-202. doi:10.1038/ng1094-195
260. Kaltschmidt, J. A., Lawrence, N., Morel, V., Balayo, T., Fernandez, B. G., Pelissier, A., . . . Martinez Arias, A. (2002). Planar polarity and actin dynamics in the epidermis of Drosophila. Nat Cell Biol, 4(12), 937-944. doi:10.1038/ncb882
261. Kantarci, S., Al-Gazali, L., Hill, R. S., Donnai, D., Black, G. C., Bieth, E., . . . Pober, B. R. (2007). Mutations in LRP2, which encodes the multiligand receptor megalin, cause Donnai-Barrow and facio-oculo-acoustico-renal syndromes. Nat Genet, 39(8), 957-959. doi:10.1038/ng2063
262. Katsanis, N., Beales, P. L., Woods, M. O., Lewis, R. A., Green, J. S., Parfrey, P. S., . . . Lupski, J. R. (2000). Mutations in MKKS cause obesity, retinal dystrophy and renal malformations associated with Bardet-Biedl syndrome. Nat Genet, 26(1), 67-70. doi:10.1038/79201
263. Kere, J., Srivastava, A. K., Montonen, O., Zonana, J., Thomas, N., Ferguson, B., . . . Schlessinger, D. (1996). X-linked anhidrotic (hypohidrotic) ectodermal dysplasia is caused by mutation in a novel transmembrane protein. Nat Genet, 13(4), 409-416. doi:10.1038/ng0895-409
264. Khan, L. A., Zhang, H., Abraham, N., Sun, L., Fleming, J. T., Buechner, M., . . . Gobel, V. (2013). Intracellular lumen extension requires ERM-1-dependent apical membrane expansion and AQP-8-mediated flux. Nat Cell Biol, 15(2), 143-156. doi:10.1038/ncb2656
265. Khokha, M. K., Hsu, D., Brunet, L. J., Dionne, M. S., & Harland, R. M. (2003). Gremlin is the BMP antagonist required for maintenance of Shh and Fgf signals during limb patterning. Nat Genet, 34(3), 303-307. doi:10.1038/ng1178
266. Khoueiry, R., Sohni, A., Thienpont, B., Luo, X., Velde, J. V., Bartoccetti, M., . . . Koh, K. P. (2017). Lineage-specific functions of TET1 in the postimplantation mouse embryo. Nat Genet, 49(7), 1061-1072. doi:10.1038/ng.3868
267. Kielar, M., Tuy, F. P., Bizzotto, S., Lebrand, C., de Juan Romero, C., Poirier, K., . . . Francis, F. (2014). Mutations in Eml1 lead to ectopic progenitors and neuronal heterotopia in mouse and human. Nat Neurosci, 17(7), 923-933. doi:10.1038/nn.3729
268. Kim, K., Ryu, S. M., Kim, S. T., Baek, G., Kim, D., Lim, K., . . . Kim, J. S. (2017). Highly efficient RNA-guided base editing in mouse embryos. Nat Biotechnol, 35(5), 435-437. doi:10.1038/nbt.3816
269. Kim, M. H., Cierpicki, T., Derewenda, U., Krowarsch, D., Feng, Y., Devedjiev, Y., . . . Derewenda, Z. S. (2003). The DCX-domain tandems of doublecortin and doublecortin-like kinase. Nat Struct Biol, 10(5), 324-333. doi:10.1038/nsb918
270. Kim, W. Y., Wang, X., Wu, Y., Doble, B. W., Patel, S., Woodgett, J. R., & Snider, W. D. (2009). GSK-3 is a master regulator of neural progenitor homeostasis. Nat Neurosci, 12(11), 1390-1397. doi:10.1038/nn.2408
271. Kimura-Yoshida, C., Mochida, K., Nakaya, M. A., Mizutani, T., & Matsuo, I. (2018). Cytoplasmic localization of GRHL3 upon epidermal differentiation triggers cell shape change for epithelial morphogenesis. Nat Commun, 9(1), 4059. doi:10.1038/s41467-018-06171-8
272. Kishino, T., Lalande, M., & Wagstaff, J. (1997). UBE3A/E6-AP mutations cause Angelman syndrome. Nat Genet, 15(1), 70-73. doi:10.1038/ng0197-70
273. Kitagawa, R., & Rose, A. M. (1999). Components of the spindle-assembly checkpoint are essential in Caenorhabditis elegans. Nat Cell Biol, 1(8), 514-521. doi:10.1038/70309
274. Kitamura, K., Yanazawa, M., Sugiyama, N., Miura, H., Iizuka-Kogo, A., Kusaka, M., . . . Morohashi, K. (2002). Mutation of ARX causes abnormal development of forebrain and testes in mice and X-linked lissencephaly with abnormal genitalia in humans. Nat Genet, 32(3), 359-369. doi:10.1038/ng1009
275. Kmita, M., Kondo, T., & Duboule, D. (2000). Targeted inversion of a polar silencer within the HoxD complex re-allocates domains of enhancer sharing. Nat Genet, 26(4), 451-454. doi:10.1038/82593
276. Kohlhase, J., Wischermann, A., Reichenbach, H., Froster, U., & Engel, W. (1998). Mutations in the SALL1 putative transcription factor gene cause Townes-Brocks syndrome. Nat Genet, 18(1), 81-83. doi:10.1038/ng0198-81
277. Kondo, S., Schutte, B. C., Richardson, R. J., Bjork, B. C., Knight, A. S., Watanabe, Y., . . . Murray, J. C. (2002). Mutations in IRF6 cause Van der Woude and popliteal pterygium syndromes. Nat Genet, 32(2), 285-289. doi:10.1038/ng985
278. Koolen, D. A., Vissers, L. E., Pfundt, R., de Leeuw, N., Knight, S. J., Regan, R., . . . de Vries, B. B. (2006). A new chromosome 17q21.31 microdeletion syndrome associated with a common inversion polymorphism. Nat Genet, 38(9), 999-1001. doi:10.1038/ng1853
279. Kosar, I., Ataseven, H., Yonem, O., Cakmak, E., Ozer, O., Ozer, H., & Topcu, O. (2010). A new variant of bile duct duplication with coexistence of distal cholangiocarcinoma. Nat Rev Gastroenterol Hepatol, 7(9), 527-530. doi:10.1038/nrgastro.2010.118
280. Koshiba-Takeuchi, K., Takeuchi, J. K., Arruda, E. P., Kathiriya, I. S., Mo, R., Hui, C. C., . . . Bruneau, B. G. (2006). Cooperative and antagonistic interactions between Sall4 and Tbx5 pattern the mouse limb and heart. Nat Genet, 38(2), 175-183. doi:10.1038/ng1707
281. Krantz, I. D., McCallum, J., DeScipio, C., Kaur, M., Gillis, L. A., Yaeger, D., . . . Jackson, L. G. (2004). Cornelia de Lange syndrome is caused by mutations in NIPBL, the human homolog of Drosophila melanogaster Nipped-B. Nat Genet, 36(6), 631-635. doi:10.1038/ng1364
282. Krebs, A. M., Mitschke, J., Lasierra Losada, M., Schmalhofer, O., Boerries, M., Busch, H., . . . Brabletz, T. (2017). The EMT-activator Zeb1 is a key factor for cell plasticity and promotes metastasis in pancreatic cancer. Nat Cell Biol, 19(5), 518-529. doi:10.1038/ncb3513
283. Krmpotic, A., Busch, D. H., Bubic, I., Gebhardt, F., Hengel, H., Hasan, M., . . . Jonjic, S. (2002). MCMV glycoprotein gp40 confers virus resistance to CD8+ T cells and NK cells in vivo. Nat Immunol, 3(6), 529-535. doi:10.1038/ni799
284. Kunath, T., Gish, G., Lickert, H., Jones, N., Pawson, T., & Rossant, J. (2003). Transgenic RNA interference in ES cell-derived embryos recapitulates a genetic null phenotype. Nat Biotechnol, 21(5), 559-561. doi:10.1038/nbt813
285. Kunst, C. B., Mezey, E., Brownstein, M. J., & Patterson, D. (1997). Mutations in SOD1 associated with amyotrophic lateral sclerosis cause novel protein interactions. Nat Genet, 15(1), 91-94. doi:10.1038/ng0197-91
286. Kurotaki, N., Imaizumi, K., Harada, N., Masuno, M., Kondoh, T., Nagai, T., . . . Matsumoto, N. (2002). Haploinsufficiency of NSD1 causes Sotos syndrome. Nat Genet, 30(4), 365-366. doi:10.1038/ng863
287. Kusumi, K., Mimoto, M. S., Covello, K. L., Beddington, R. S., Krumlauf, R., & Dunwoodie, S. L. (2004). Dll3 pudgy mutation differentially disrupts dynamic expression of somite genes. Genesis, 39(2), 115-121. doi:10.1002/gene.20034
288. Ladero, J. M. (2008). Influence of polymorphic N-acetyltransferases on non-malignant spontaneous disorders and on response to drugs. Curr Drug Metab, 9(6), 532-537.
289. Lagendijk, A. K., Gomez, G. A., Baek, S., Hesselson, D., Hughes, W. E., Paterson, S., . . . Hogan, B. M. (2017). Live imaging molecular changes in junctional tension upon VE-cadherin in zebrafish. Nat Commun, 8(1), 1402. doi:10.1038/s41467-017-01325-6
290. Lander, R., Nasr, T., Ochoa, S. D., Nordin, K., Prasad, M. S., & Labonne, C. (2013). Interactions between Twist and other core epithelial-mesenchymal transition factors are controlled by GSK3-mediated phosphorylation. Nat Commun, 4, 1542. doi:10.1038/ncomms2543
291. Lanford, P. J., Lan, Y., Jiang, R., Lindsell, C., Weinmaster, G., Gridley, T., & Kelley, M. W. (1999). Notch signalling pathway mediates hair cell development in mammalian cochlea. Nat Genet, 21(3), 289-292. doi:10.1038/6804
292. Lang, M. R., Lapierre, L. A., Frotscher, M., Goldenring, J. R., & Knapik, E. W. (2006). Secretory COPII coat component Sec23a is essential for craniofacial chondrocyte maturation. Nat Genet, 38(10), 1198-1203. doi:10.1038/ng1880
293. Langefeld, C. D., Ainsworth, H. C., Cunninghame Graham, D. S., Kelly, J. A., Comeau, M. E., Marion, M. C., . . . Vyse, T. J. (2017). Transancestral mapping and genetic load in systemic lupus erythematosus. Nat Commun, 8, 16021. doi:10.1038/ncomms16021
294. Langenau, D. M., & Zon, L. I. (2005). The zebrafish: a new model of T-cell and thymic development. Nat Rev Immunol, 5(4), 307-317. doi:10.1038/nri1590
295. Langer, D., Martianov, I., Alpern, D., Rhinn, M., Keime, C., Dolle, P., . . . Davidson, I. (2016). Essential role of the TFIID subunit TAF4 in murine embryogenesis and embryonic stem cell differentiation. Nat Commun, 7, 11063. doi:10.1038/ncomms11063
296. Larocque, D., Galarneau, A., Liu, H. N., Scott, M., Almazan, G., & Richard, S. (2005). Protection of p27(Kip1) mRNA by quaking RNA binding proteins promotes oligodendrocyte differentiation. Nat Neurosci, 8(1), 27-33. doi:10.1038/nn1359
297. Larsson, N. G., Wang, J., Wilhelmsson, H., Oldfors, A., Rustin, P., Lewandoski, M., . . . Clayton, D. A. (1998). Mitochondrial transcription factor A is necessary for mtDNA maintenance and embryogenesis in mice. Nat Genet, 18(3), 231-236. doi:10.1038/ng0398-231
298. Le Goff, C., Morice-Picard, F., Dagoneau, N., Wang, L. W., Perrot, C., Crow, Y. J., . . . Cormier-Daire, V. (2008). ADAMTSL2 mutations in geleophysic dysplasia demonstrate a role for ADAMTS-like proteins in TGF-beta bioavailability regulation. Nat Genet, 40(9), 1119-1123. doi:10.1038/ng.199
299. Ledbetter, D. H. (2009). Chaos in the embryo. Nat Med, 15(5), 490-491. doi:10.1038/nm0509-490
300. Lee, J. H., Jung, S. M., Yang, K. M., Bae, E., Ahn, S. G., Park, J. S., . . . Park, S. H. (2017). A20 promotes metastasis of aggressive basal-like breast cancers through multi-monoubiquitylation of Snail1. Nat Cell Biol, 19(10), 1260-1273. doi:10.1038/ncb3609
301. Lee, O. K., Frese, K. K., James, J. S., Chadda, D., Chen, Z. H., Javier, R. T., & Cho, K. O. (2003). Discs-Large and Strabismus are functionally linked to plasma membrane formation. Nat Cell Biol, 5(11), 987-993. doi:10.1038/ncb1055
302. Lefebvre, S., Burlet, P., Liu, Q., Bertrandy, S., Clermont, O., Munnich, A., . . . Melki, J. (1997). Correlation between severity and SMN protein level in spinal muscular atrophy. Nat Genet, 16(3), 265-269. doi:10.1038/ng0797-265
303. Legouis, R., Gansmuller, A., Sookhareea, S., Bosher, J. M., Baillie, D. L., & Labouesse, M. (2000). LET-413 is a basolateral protein required for the assembly of adherens junctions in Caenorhabditis elegans. Nat Cell Biol, 2(7), 415-422. doi:10.1038/35017046
304. Levanat, S., Gorlin, R. J., Fallet, S., Johnson, D. R., Fantasia, J. E., & Bale, A. E. (1996). A two-hit model for developmental defects in Gorlin syndrome. Nat Genet, 12(1), 85-87. doi:10.1038/ng0196-85
305. Li, J., & Li, W. X. (2006). A novel function of Drosophila eIF4A as a negative regulator of Dpp/BMP signalling that mediates SMAD degradation. Nat Cell Biol, 8(12), 1407-1414. doi:10.1038/ncb1506
306. Li, M., Zhang, W. K., Benvin, N. M., Zhou, X., Su, D., Li, H., . . . Yang, J. (2017). Structural basis of dual Ca(2+)/pH regulation of the endolysosomal TRPML1 channel. Nat Struct Mol Biol, 24(3), 205-213. doi:10.1038/nsmb.3362
307. Li, Q. Y., Newbury-Ecob, R. A., Terrett, J. A., Wilson, D. I., Curtis, A. R., Yi, C. H., . . . Brook, J. D. (1997). Holt-Oram syndrome is caused by mutations in TBX5, a member of the Brachyury (T) gene family. Nat Genet, 15(1), 21-29. doi:10.1038/ng0197-21
308. Li, X. C., Everett, L. A., Lalwani, A. K., Desmukh, D., Friedman, T. B., Green, E. D., & Wilcox, E. R. (1998). A mutation in PDS causes non-syndromic recessive deafness. Nat Genet, 18(3), 215-217. doi:10.1038/ng0398-215
309. Li, Z., Godinho, F. J., Klusmann, J. H., Garriga-Canut, M., Yu, C., & Orkin, S. H. (2005). Developmental stage-selective effect of somatically mutated leukemogenic transcription factor GATA1. Nat Genet, 37(6), 613-619. doi:10.1038/ng1566
310. Lim, K. C., Lakshmanan, G., Crawford, S. E., Gu, Y., Grosveld, F., & Engel, J. D. (2000). Gata3 loss leads to embryonic lethality due to noradrenaline deficiency of the sympathetic nervous system. Nat Genet, 25(2), 209-212. doi:10.1038/76080
311. Lin, Y., Guo, X., Zhao, B., Liu, J., Da, M., Wen, Y., . . . Hu, Z. (2015). Association analysis identifies new risk loci for congenital heart disease in Chinese populations. Nat Commun, 6, 8082. doi:10.1038/ncomms9082
312. Lindsay, E. A. (2001). Chromosomal microdeletions: dissecting del22q11 syndrome. Nat Rev Genet, 2(11), 858-868. doi:10.1038/35098574
313. Lindstrom, S., Thompson, D. J., Paterson, A. D., Li, J., Gierach, G. L., Scott, C., . . . Tamimi, R. M. (2014). Genome-wide association study identifies multiple loci associated with both mammographic density and breast cancer risk. Nat Commun, 5, 5303. doi:10.1038/ncomms6303
314. Lipkin, S. M., Moens, P. B., Wang, V., Lenzi, M., Shanmugarajah, D., Gilgeous, A., . . . Cohen, P. E. (2002). Meiotic arrest and aneuploidy in MLH3-deficient mice. Nat Genet, 31(4), 385-390. doi:10.1038/ng931
315. Littlewood Evans, A., & Muller, U. (2000). Stereocilia defects in the sensory hair cells of the inner ear in mice deficient in integrin alpha8beta1. Nat Genet, 24(4), 424-428. doi:10.1038/74286
316. Liu, F., Thirumangalathu, S., Gallant, N. M., Yang, S. H., Stoick-Cooper, C. L., Reddy, S. T., . . . Millar, S. E. (2007). Wnt-beta-catenin signaling initiates taste papilla development. Nat Genet, 39(1), 106-112. doi:10.1038/ng1932
317. Liu, J., & Ma, J. (2013). Dampened regulates the activating potency of Bicoid and the embryonic patterning outcome in Drosophila. Nat Commun, 4, 2968. doi:10.1038/ncomms3968
318. Liu, Q., Acharya, P., Dolan, M. A., Zhang, P., Guzzo, C., Lu, J., . . . Lusso, P. (2017). Quaternary contact in the initial interaction of CD4 with the HIV-1 envelope trimer. Nat Struct Mol Biol, 24(4), 370-378. doi:10.1038/nsmb.3382
319. Liu, S. C., Hsu, T., Chang, Y. S., Chung, A. K., Jiang, S. S., OuYang, C. N., . . . Tsang, N. M. (2018). Cytoplasmic LIF reprograms invasive mode to enhance NPC dissemination through modulating YAP1-FAK/PXN signaling. Nat Commun, 9(1), 5105. doi:10.1038/s41467-018-07660-6
320. Liu, X., Yagi, H., Saeed, S., Bais, A. S., Gabriel, G. C., Chen, Z., . . . Lo, C. W. (2017). The complex genetics of hypoplastic left heart syndrome. Nat Genet, 49(7), 1152-1159. doi:10.1038/ng.3870
321. Liu, Z., Steward, R., & Luo, L. (2000). Drosophila Lis1 is required for neuroblast proliferation, dendritic elaboration and axonal transport. Nat Cell Biol, 2(11), 776-783. doi:10.1038/35041011
322. Lo Sardo, V., Zuccato, C., Gaudenzi, G., Vitali, B., Ramos, C., Tartari, M., . . . Cattaneo, E. (2012). An evolutionary recent neuroepithelial cell adhesion function of huntingtin implicates ADAM10-Ncadherin. Nat Neurosci, 15(5), 713-721. doi:10.1038/nn.3080
323. Lopez, J. C. (2000). Down the ataxin-1 track. Nat Rev Neurosci, 1(3), 154. doi:10.1038/35044505
324. Lorenzo, A., Yuan, M., Zhang, Z., Paganetti, P. A., Sturchler-Pierrat, C., Staufenbiel, M., . . . Yankner, B. A. (2000). Amyloid beta interacts with the amyloid precursor protein: a potential toxic mechanism in Alzheimer's disease. Nat Neurosci, 3(5), 460-464. doi:10.1038/74833
325. Lowry, R. B., Jabs, E. W., Graham, G. E., Gerritsen, J., & Fleming, J. (2001). Syndrome of coronal craniosynostosis, Klippel-Feil anomaly, and sprengel shoulder with and without Pro250Arg mutation in the FGFR3 gene. Am J Med Genet, 104(2), 112-119.
326. Lu, I. L., Chen, C., Tung, C. Y., Chen, H. H., Pan, J. P., Chang, C. H., . . . Tsai, J. W. (2018). Identification of genes associated with cortical malformation using a transposon-mediated somatic mutagenesis screen in mice. Nat Commun, 9(1), 2498. doi:10.1038/s41467-018-04880-8
327. Lu, W., Peissel, B., Babakhanlou, H., Pavlova, A., Geng, L., Fan, X., . . . Zhou, J. (1997). Perinatal lethality with kidney and pancreas defects in mice with a targetted Pkd1 mutation. Nat Genet, 17(2), 179-181. doi:10.1038/ng1097-179
328. Luo, L., & Zong, H. (2001). Single neuron labeling and genetic manipulation. Nat Neurosci, 4 Suppl, 1158-1159. doi:10.1038/nn1101-1158
329. Luxan, G., Casanova, J. C., Martinez-Poveda, B., Prados, B., D'Amato, G., MacGrogan, D., . . . de la Pompa, J. L. (2013). Mutations in the NOTCH pathway regulator MIB1 cause left ventricular noncompaction cardiomyopathy. Nat Med, 19(2), 193-201. doi:10.1038/nm.3046
330. Lynch, D. C., Revil, T., Schwartzentruber, J., Bhoj, E. J., Innes, A. M., Lamont, R. E., . . . Bernier, F. P. (2014). Disrupted auto-regulation of the spliceosomal gene SNRPB causes cerebro-costo-mandibular syndrome. Nat Commun, 5, 4483. doi:10.1038/ncomms5483
331. Macchia, P. E., Lapi, P., Krude, H., Pirro, M. T., Missero, C., Chiovato, L., . . . Di Lauro, R. (1998). PAX8 mutations associated with congenital hypothyroidism caused by thyroid dysgenesis. Nat Genet, 19(1), 83-86. doi:10.1038/ng0598-83
332. Macedo, J. C., Vaz, S., Bakker, B., Ribeiro, R., Bakker, P. L., Escandell, J. M., . . . Logarinho, E. (2018). FoxM1 repression during human aging leads to mitotic decline and aneuploidy-driven full senescence. Nat Commun, 9(1), 2834. doi:10.1038/s41467-018-05258-6
333. Maden, M. (1999). Heads or tails? Retinoic acid will decide. Bioessays, 21(10), 809-812. doi:10.1002/(sici)1521-1878(199910)21:10<809::Aid-bies2>3.0.Co;2-0
334. Mae, S. I., Shono, A., Shiota, F., Yasuno, T., Kajiwara, M., Gotoda-Nishimura, N., . . . Osafune, K. (2013). Monitoring and robust induction of nephrogenic intermediate mesoderm from human pluripotent stem cells. Nat Commun, 4, 1367. doi:10.1038/ncomms2378
335. Mahid, S. S., Colliver, D. W., Crawford, N. P., Martini, B. D., Doll, M. A., Hein, D. W., . . . Galandiuk, S. (2007). Characterization of N-acetyltransferase 1 and 2 polymorphisms and haplotype analysis for inflammatory bowel disease and sporadic colorectal carcinoma. BMC Med Genet, 8, 28. doi:10.1186/1471-2350-8-28
336. Malter, H. E., Iber, J. C., Willemsen, R., de Graaff, E., Tarleton, J. C., Leisti, J., . . . Oostra, B. A. (1997). Characterization of the full fragile X syndrome mutation in fetal gametes. Nat Genet, 15(2), 165-169. doi:10.1038/ng0297-165
337. Manak, J. R., Dike, S., Sementchenko, V., Kapranov, P., Biemar, F., Long, J., . . . Gingeras, T. R. (2006). Biological function of unannotated transcription during the early development of Drosophila melanogaster. Nat Genet, 38(10), 1151-1158. doi:10.1038/ng1875
338. Manak, J. R., Wen, H., Van, T., Andrejka, L., & Lipsick, J. S. (2007). Loss of Drosophila Myb interrupts the progression of chromosome condensation. Nat Cell Biol, 9(5), 581-587. doi:10.1038/ncb1580
339. Manikumar, G., Gaetano, K., Wani, M. C., Taylor, H., Hughes, T. J., Warner, J., . . . Wall, M. E. (1989). Plant antimutagenic agents, 5. Isolation and structure of two new isoflavones, fremontin and fremontone from Psorothamnus fremontii. J Nat Prod, 52(4), 769-773.
340. Mara, A., Schroeder, J., Chalouni, C., & Holley, S. A. (2007). Priming, initiation and synchronization of the segmentation clock by deltaD and deltaC. Nat Cell Biol, 9(5), 523-530. doi:10.1038/ncb1578
341. Marsh, A. P., Heron, D., Edwards, T. J., Quartier, A., Galea, C., Nava, C., . . . Depienne, C. (2017). Mutations in DCC cause isolated agenesis of the corpus callosum with incomplete penetrance. Nat Genet, 49(4), 511-514. doi:10.1038/ng.3794
342. Marshall, W. F. (2010). Cilia self-organize in response to planar cell polarity and flow. Nat Cell Biol, 12(4), 314-315. doi:10.1038/ncb0410-314
343. Marthiens, V., Rujano, M. A., Pennetier, C., Tessier, S., Paul-Gilloteaux, P., & Basto, R. (2013). Centrosome amplification causes microcephaly. Nat Cell Biol, 15(7), 731-740. doi:10.1038/ncb2746
344. Martin, F., Menetret, J. F., Simonetti, A., Myasnikov, A. G., Vicens, Q., Prongidi-Fix, L., . . . Eriani, G. (2016). Ribosomal 18S rRNA base pairs with mRNA during eukaryotic translation initiation. Nat Commun, 7, 12622. doi:10.1038/ncomms12622
345. Matsuo, T., Osumi-Yamashita, N., Noji, S., Ohuchi, H., Koyama, E., Myokai, F., . . . et al. (1993). A mutation in the Pax-6 gene in rat small eye is associated with impaired migration of midbrain crest cells. Nat Genet, 3(4), 299-304. doi:10.1038/ng0493-299
346. Mavrakis, M., Azou-Gros, Y., Tsai, F. C., Alvarado, J., Bertin, A., Iv, F., . . . Lecuit, T. (2014). Septins promote F-actin ring formation by crosslinking actin filaments into curved bundles. Nat Cell Biol, 16(4), 322-334. doi:10.1038/ncb2921
347. Mavrogiannis, L. A., Antonopoulou, I., Baxova, A., Kutilek, S., Kim, C. A., Sugayama, S. M., . . . Wilkie, A. O. (2001). Haploinsufficiency of the human homeobox gene ALX4 causes skull ossification defects. Nat Genet, 27(1), 17-18. doi:10.1038/83703
348. McCallum, C. M., Comai, L., Greene, E. A., & Henikoff, S. (2000). Targeted screening for induced mutations. Nat Biotechnol, 18(4), 455-457. doi:10.1038/74542
349. McCarthy, N. (2013). Glioblastoma: histone mutations take the MYCN. Nat Rev Cancer, 13(6), 382-383. doi:10.1038/nrc3527
350. McDonald, D. (2004). A SNAP decision in neural cell fate. Nat Cell Biol, 6(3), 187. doi:10.1038/ncb0304-187
351. McPherron, A. C., Lawler, A. M., & Lee, S. J. (1999). Regulation of anterior/posterior patterning of the axial skeleton by growth/differentiation factor 11. Nat Genet, 22(3), 260-264. doi:10.1038/10320
352. Mei, L., & Xiong, W. C. (2008). Neuregulin 1 in neural development, synaptic plasticity and schizophrenia. Nat Rev Neurosci, 9(6), 437-452. doi:10.1038/nrn2392
353. Melero, R., Buchwald, G., Castano, R., Raabe, M., Gil, D., Lazaro, M., . . . Llorca, O. (2012). The cryo-EM structure of the UPF-EJC complex shows UPF1 poised toward the RNA 3' end. Nat Struct Mol Biol, 19(5), 498-505, s491-492. doi:10.1038/nsmb.2287
354. Mercer, J. F., Grimes, A., Ambrosini, L., Lockhart, P., Paynter, J. A., Dierick, H., & Glover, T. W. (1994). Mutations in the murine homologue of the Menkes gene in dappled and blotchy mice. Nat Genet, 6(4), 374-378. doi:10.1038/ng0494-374
355. Merks, A. M., Swinarski, M., Meyer, A. M., Muller, N. V., Ozcan, I., Donat, S., . . . Panakova, D. (2018). Planar cell polarity signalling coordinates heart tube remodelling through tissue-scale polarisation of actomyosin activity. Nat Commun, 9(1), 2161. doi:10.1038/s41467-018-04566-1
356. Merrell, A. J., Ellis, B. J., Fox, Z. D., Lawson, J. A., Weiss, J. A., & Kardon, G. (2015). Muscle connective tissue controls development of the diaphragm and is a source of congenital diaphragmatic hernias. Nat Genet, 47(5), 496-504. doi:10.1038/ng.3250
357. Mi, L. Z., Lu, C., Li, Z., Nishida, N., Walz, T., & Springer, T. A. (2011). Simultaneous visualization of the extracellular and cytoplasmic domains of the epidermal growth factor receptor. Nat Struct Mol Biol, 18(9), 984-989. doi:10.1038/nsmb.2092
358. Miettinen, P. J., Chin, J. R., Shum, L., Slavkin, H. C., Shuler, C. F., Derynck, R., & Werb, Z. (1999). Epidermal growth factor receptor function is necessary for normal craniofacial development and palate closure. Nat Genet, 22(1), 69-73. doi:10.1038/8773
359. Milunsky, J. M., Zhao, G., Maher, T. A., Colby, R., & Everman, D. B. (2006). LADD syndrome is caused by FGF10 mutations. Clin Genet, 69(4), 349-354. doi:10.1111/j.1399-0004.2006.00597.x
360. Moon, A. M., & Capecchi, M. R. (2000). Fgf8 is required for outgrowth and patterning of the limbs. Nat Genet, 26(4), 455-459. doi:10.1038/82601
361. Morano, I., Chai, G. X., Baltas, L. G., Lamounier-Zepter, V., Lutsch, G., Kott, M., . . . Bader, M. (2000). Smooth-muscle contraction without smooth-muscle myosin. Nat Cell Biol, 2(6), 371-375. doi:10.1038/35014065
362. Moreno-Mateos, M. A., Vejnar, C. E., Beaudoin, J. D., Fernandez, J. P., Mis, E. K., Khokha, M. K., & Giraldez, A. J. (2015). CRISPRscan: designing highly efficient sgRNAs for CRISPR-Cas9 targeting in vivo. Nat Methods, 12(10), 982-988. doi:10.1038/nmeth.3543
363. Mortlock, D. P., & Innis, J. W. (1997). Mutation of HOXA13 in hand-foot-genital syndrome. Nat Genet, 15(2), 179-180. doi:10.1038/ng0297-179
364. Mortlock, D. P., Post, L. C., & Innis, J. W. (1996). The molecular basis of hypodactyly (Hd): a deletion in Hoxa 13 leads to arrest of digital arch formation. Nat Genet, 13(3), 284-289. doi:10.1038/ng0796-284
365. Mroczek, S., Chlebowska, J., Kulinski, T. M., Gewartowska, O., Gruchota, J., Cysewski, D., . . . Dziembowski, A. (2017). The non-canonical poly(A) polymerase FAM46C acts as an onco-suppressor in multiple myeloma. Nat Commun, 8(1), 619. doi:10.1038/s41467-017-00578-5
366. Muenke, M., Schell, U., Hehr, A., Robin, N. H., Losken, H. W., Schinzel, A., . . . et al. (1994). A common mutation in the fibroblast growth factor receptor 1 gene in Pfeiffer syndrome. Nat Genet, 8(3), 269-274. doi:10.1038/ng1194-269
367. Mullins, M. C. (2002). Building-blocks of embryogenesis. Nat Genet, 31(2), 125-126. doi:10.1038/ng0602-125
368. Munroe, R. J., Bergstrom, R. A., Zheng, Q. Y., Libby, B., Smith, R., John, S. W., . . . Schimenti, J. C. (2000). Mouse mutants from chemically mutagenized embryonic stem cells. Nat Genet, 24(3), 318-321. doi:10.1038/73563
369. Murayama, E., Sarris, M., Redd, M., Le Guyader, D., Vivier, C., Horsley, W., . . . Herbomel, P. (2015). NACA deficiency reveals the crucial role of somite-derived stromal cells in haematopoietic niche formation. Nat Commun, 6, 8375. doi:10.1038/ncomms9375
370. Murphey, R. D., & Zon, L. I. (2002). Attack of the fish clones. Nat Biotechnol, 20(8), 785-786. doi:10.1038/nbt0802-785
371. Myers, J. B., Zaegel, V., Coultrap, S. J., Miller, A. P., Bayer, K. U., & Reichow, S. L. (2017). The CaMKII holoenzyme structure in activation-competent conformations. Nat Commun, 8, 15742. doi:10.1038/ncomms15742
372. Nair, K. S., Hmani-Aifa, M., Ali, Z., Kearney, A. L., Ben Salem, S., Macalinao, D. G., . . . John, S. W. (2011). Alteration of the serine protease PRSS56 causes angle-closure glaucoma in mice and posterior microphthalmia in humans and mice. Nat Genet, 43(6), 579-584. doi:10.1038/ng.813
373. Najm, J., Horn, D., Wimplinger, I., Golden, J. A., Chizhikov, V. V., Sudi, J., . . . Kutsche, K. (2008). Mutations of CASK cause an X-linked brain malformation phenotype with microcephaly and hypoplasia of the brainstem and cerebellum. Nat Genet, 40(9), 1065-1067. doi:10.1038/ng.194
374. Naka, H., Nakamura, S., Shimazaki, T., & Okano, H. (2008). Requirement for COUP-TFI and II in the temporal specification of neural stem cells in CNS development. Nat Neurosci, 11(9), 1014-1023. doi:10.1038/nn.2168
375. Nakago, S., Hadfield, R. M., Zondervan, K. T., Mardon, H., Manek, S., Weeks, D. E., . . . Kennedy, S. (2001). Association between endometriosis and N-acetyl transferase 2 polymorphisms in a UK population. Mol Hum Reprod, 7(11), 1079-1083.
376. Nakamura, Y., Yamamoto, K., He, X., Otsuki, B., Kim, Y., Murao, H., . . . Akiyama, H. (2011). Wwp2 is essential for palatogenesis mediated by the interaction between Sox9 and mediator subunit 25. Nat Commun, 2, 251. doi:10.1038/ncomms1242
377. Nammo, T., Yamagata, K., Tanaka, T., Kodama, T., Sladek, F. M., Fukui, K., . . . Shimomura, I. (2008). Expression of HNF-4alpha (MODY1), HNF-1beta (MODY5), and HNF-1alpha (MODY3) proteins in the developing mouse pancreas. Gene Expr Patterns, 8(2), 96-106. doi:10.1016/j.modgep.2007.09.006
378. Nery, S., Fishell, G., & Corbin, J. G. (2002). The caudal ganglionic eminence is a source of distinct cortical and subcortical cell populations. Nat Neurosci, 5(12), 1279-1287. doi:10.1038/nn971
379. Netchine, I., Sobrier, M. L., Krude, H., Schnabel, D., Maghnie, M., Marcos, E., . . . Amselem, S. (2000). Mutations in LHX3 result in a new syndrome revealed by combined pituitary hormone deficiency. Nat Genet, 25(2), 182-186. doi:10.1038/76041
380. Ng, D., Thakker, N., Corcoran, C. M., Donnai, D., Perveen, R., Schneider, A., . . . Biesecker, L. G. (2004). Oculofaciocardiodental and Lenz microphthalmia syndromes result from distinct classes of mutations in BCOR. Nat Genet, 36(4), 411-416. doi:10.1038/ng1321
381. Ng, L., Hurley, J. B., Dierks, B., Srinivas, M., Salto, C., Vennstrom, B., . . . Forrest, D. (2001). A thyroid hormone receptor that is required for the development of green cone photoreceptors. Nat Genet, 27(1), 94-98. doi:10.1038/83829
382. Ng, R. K., & Gurdon, J. B. (2008). Epigenetic memory of an active gene state depends on histone H3.3 incorporation into chromatin in the absence of transcription. Nat Cell Biol, 10(1), 102-109. doi:10.1038/ncb1674
383. Nickols, J. C., Valentine, W., Kanwal, S., & Carter, B. D. (2003). Activation of the transcription factor NF-kappaB in Schwann cells is required for peripheral myelin formation. Nat Neurosci, 6(2), 161-167. doi:10.1038/nn995
384. Nikaido, M., Kawakami, A., Sawada, A., Furutani-Seiki, M., Takeda, H., & Araki, K. (2002). Tbx24, encoding a T-box protein, is mutated in the zebrafish somite-segmentation mutant fused somites. Nat Genet, 31(2), 195-199. doi:10.1038/ng899
385. Nishimura, G., Nishimura, H., Tanaka, Y., Makita, Y., Ikegawa, S., Ghadami, M., . . . Niikawa, N. (2002). Camurati-Engelmann disease type II: progressive diaphyseal dysplasia with striations of the bones. Am J Med Genet, 107(1), 5-11.
386. Nishimura, T., Fukata, Y., Kato, K., Yamaguchi, T., Matsuura, Y., Kamiguchi, H., & Kaibuchi, K. (2003). CRMP-2 regulates polarized Numb-mediated endocytosis for axon growth. Nat Cell Biol, 5(9), 819-826. doi:10.1038/ncb1039
387. Nishiwaki, K., Kubota, Y., Chigira, Y., Roy, S. K., Suzuki, M., Schvarzstein, M., . . . Matsumoto, K. (2004). An NDPase links ADAM protease glycosylation with organ morphogenesis in C. elegans. Nat Cell Biol, 6(1), 31-37. doi:10.1038/ncb1079
388. Noel, E. S., Verhoeven, M., Lagendijk, A. K., Tessadori, F., Smith, K., Choorapoikayil, S., . . . Bakkers, J. (2013). A Nodal-independent and tissue-intrinsic mechanism controls heart-looping chirality. Nat Commun, 4, 2754. doi:10.1038/ncomms3754
389. Nordqvist, K., & Lovell-Badge, R. (1994). Setbacks on the road to sexual fulfillment. Nat Genet, 7(1), 7-9. doi:10.1038/ng0594-7
390. Nutt, S. L., Vambrie, S., Steinlein, P., Kozmik, Z., Rolink, A., Weith, A., & Busslinger, M. (1999). Independent regulation of the two Pax5 alleles during B-cell development. Nat Genet, 21(4), 390-395. doi:10.1038/7720
391. Nyholt, D. R., Low, S. K., Anderson, C. A., Painter, J. N., Uno, S., Morris, A. P., . . . Montgomery, G. W. (2012). Genome-wide association meta-analysis identifies new endometriosis risk loci. Nat Genet, 44(12), 1355-1359. doi:10.1038/ng.2445
392. Okada, T., Sinha, S., Esposito, I., Schiavon, G., Lopez-Lago, M. A., Su, W., . . . Giancotti, F. G. (2015). The Rho GTPase Rnd1 suppresses mammary tumorigenesis and EMT by restraining Ras-MAPK signalling. Nat Cell Biol, 17(1), 81-94. doi:10.1038/ncb3082
393. Okawa, A., Nakamura, I., Goto, S., Moriya, H., Nakamura, Y., & Ikegawa, S. (1998). Mutation in Npps in a mouse model of ossification of the posterior longitudinal ligament of the spine. Nat Genet, 19(3), 271-273. doi:10.1038/956
394. Oldridge, M., Fortuna, A. M., Maringa, M., Propping, P., Mansour, S., Pollitt, C., . . . Wilkie, A. O. (2000). Dominant mutations in ROR2, encoding an orphan receptor tyrosine kinase, cause brachydactyly type B. Nat Genet, 24(3), 275-278. doi:10.1038/73495
395. Otto, E. A., Schermer, B., Obara, T., O'Toole, J. F., Hiller, K. S., Mueller, A. M., . . . Hildebrandt, F. (2003). Mutations in INVS encoding inversin cause nephronophthisis type 2, linking renal cystic disease to the function of primary cilia and left-right axis determination. Nat Genet, 34(4), 413-420. doi:10.1038/ng1217
396. Ouimette, J. F., Jolin, M. L., L'Honore, A., Gifuni, A., & Drouin, J. (2010). Divergent transcriptional activities determine limb identity. Nat Commun, 1, 35. doi:10.1038/ncomms1036
397. Page-McCaw, A., Ewald, A. J., & Werb, Z. (2007). Matrix metalloproteinases and the regulation of tissue remodelling. Nat Rev Mol Cell Biol, 8(3), 221-233. doi:10.1038/nrm2125
398. Pandolfi, P. P., Roth, M. E., Karis, A., Leonard, M. W., Dzierzak, E., Grosveld, F. G., . . . Lindenbaum, M. H. (1995). Targeted disruption of the GATA3 gene causes severe abnormalities in the nervous system and in fetal liver haematopoiesis. Nat Genet, 11(1), 40-44. doi:10.1038/ng0995-40
399. Parma, P., Radi, O., Vidal, V., Chaboissier, M. C., Dellambra, E., Valentini, S., . . . Camerino, G. (2006). R-spondin1 is essential in sex determination, skin differentiation and malignancy. Nat Genet, 38(11), 1304-1309. doi:10.1038/ng1907
400. Parra, L. M., & Zou, Y. (2010). Sonic hedgehog induces response of commissural axons to Semaphorin repulsion during midline crossing. Nat Neurosci, 13(1), 29-35. doi:10.1038/nn.2457
401. Parvari, R., Hershkovitz, E., Grossman, N., Gorodischer, R., Loeys, B., Zecic, A., . . . Gelb, B. D. (2002). Mutation of TBCE causes hypoparathyroidism-retardation-dysmorphism and autosomal recessive Kenny-Caffey syndrome. Nat Genet, 32(3), 448-452. doi:10.1038/ng1012
402. Paszty, C., Mohandas, N., Stevens, M. E., Loring, J. F., Liebhaber, S. A., Brion, C. M., & Rubin, E. M. (1995). Lethal alpha-thalassaemia created by gene targeting in mice and its genetic rescue. Nat Genet, 11(1), 33-39. doi:10.1038/ng0995-33
403. Patterson, M. (2000). Spermatogenesis. Give me a break. Nat Rev Genet, 1(2), 89. doi:10.1038/35038527
404. Paw, B. H., Davidson, A. J., Zhou, Y., Li, R., Pratt, S. J., Lee, C., . . . Zon, L. I. (2003). Cell-specific mitotic defect and dyserythropoiesis associated with erythroid band 3 deficiency. Nat Genet, 34(1), 59-64. doi:10.1038/ng1137
405. Payton, S. (2013). Genetics: DSTYK gene linked to urinary tract defects. Nat Rev Urol, 10(9), 492. doi:10.1038/nrurol.2013.171
406. Pellegata, N. S., Dieguez-Lucena, J. L., Joensuu, T., Lau, S., Montgomery, K. T., Krahe, R., . . . de la Chapelle, A. (2000). Mutations in KERA, encoding keratocan, cause cornea plana. Nat Genet, 25(1), 91-95. doi:10.1038/75664
407. Pennisi, D., Gardner, J., Chambers, D., Hosking, B., Peters, J., Muscat, G., . . . Koopman, P. (2000). Mutations in Sox18 underlie cardiovascular and hair follicle defects in ragged mice. Nat Genet, 24(4), 434-437. doi:10.1038/74301
408. Perez-Mockus, G., Mazouni, K., Roca, V., Corradi, G., Conte, V., & Schweisguth, F. (2017). Spatial regulation of contractility by Neuralized and Bearded during furrow invagination in Drosophila. Nat Commun, 8(1), 1594. doi:10.1038/s41467-017-01482-8
409. Petters, R. M., Alexander, C. A., Wells, K. D., Collins, E. B., Sommer, J. R., Blanton, M. R., . . . Wong, F. (1997). Genetically engineered large animal model for studying cone photoreceptor survival and degeneration in retinitis pigmentosa. Nat Biotechnol, 15(10), 965-970. doi:10.1038/nbt1097-965
410. Philipp, M., Brede, M. E., Hadamek, K., Gessler, M., Lohse, M. J., & Hein, L. (2002). Placental alpha(2)-adrenoceptors control vascular development at the interface between mother and embryo. Nat Genet, 31(3), 311-315. doi:10.1038/ng919
411. Piazza, R., Magistroni, V., Redaelli, S., Mauri, M., Massimino, L., Sessa, A., . . . Gambacorti-Passerini, C. (2018). SETBP1 induces transcription of a network of development genes by acting as an epigenetic hub. Nat Commun, 9(1), 2192. doi:10.1038/s41467-018-04462-8
412. Pinto, L., Drechsel, D., Schmid, M. T., Ninkovic, J., Irmler, M., Brill, M. S., . . . Gotz, M. (2009). AP2gamma regulates basal progenitor fate in a region- and layer-specific manner in the developing cortex. Nat Neurosci, 12(10), 1229-1237. doi:10.1038/nn.2399
413. Piontek, K., Menezes, L. F., Garcia-Gonzalez, M. A., Huso, D. L., & Germino, G. G. (2007). A critical developmental switch defines the kinetics of kidney cyst formation after loss of Pkd1. Nat Med, 13(12), 1490-1495. doi:10.1038/nm1675
414. Polesello, C., Delon, I., Valenti, P., Ferrer, P., & Payre, F. (2002). Dmoesin controls actin-based cell shape and polarity during Drosophila melanogaster oogenesis. Nat Cell Biol, 4(10), 782-789. doi:10.1038/ncb856
415. Pollarolo, G., Schulz, J. G., Munck, S., & Dotti, C. G. (2011). Cytokinesis remnants define first neuronal asymmetry in vivo. Nat Neurosci, 14(12), 1525-1533. doi:10.1038/nn.2976
416. Posavec Marjanovic, M., Hurtado-Bages, S., Lassi, M., Valero, V., Malinverni, R., Delage, H., . . . Buschbeck, M. (2017). MacroH2A1.1 regulates mitochondrial respiration by limiting nuclear NAD(+) consumption. Nat Struct Mol Biol, 24(11), 902-910. doi:10.1038/nsmb.3481
417. Possuelo, L. G., Castelan, J. A., de Brito, T. C., Ribeiro, A. W., Cafrune, P. I., Picon, P. D., . . . Zaha, A. (2008). Association of slow N-acetyltransferase 2 profile and anti-TB drug-induced hepatotoxicity in patients from Southern Brazil. Eur J Clin Pharmacol, 64(7), 673-681. doi:10.1007/s00228-008-0484-8
418. Postlethwait, J. H., & Weiser, K. (1973). Vitellogenesis induced by juvenile hormone in the female sterile mutant apterous-four in Drosophila melanogaster. Nat New Biol, 244(139), 284-285.
419. Prince, K. L., Walvoord, E. C., & Rhodes, S. J. (2011). The role of homeodomain transcription factors in heritable pituitary disease. Nat Rev Endocrinol, 7(12), 727-737. doi:10.1038/nrendo.2011.119
420. Putoux, A., Thomas, S., Coene, K. L., Davis, E. E., Alanay, Y., Ogur, G., . . . Attie-Bitach, T. (2011). KIF7 mutations cause fetal hydrolethalus and acrocallosal syndromes. Nat Genet, 43(6), 601-606. doi:10.1038/ng.826
421. Raab, M., Sanhaji, M., Matthess, Y., Horlin, A., Lorenz, I., Dotsch, C., . . . Strebhardt, K. (2018). PLK1 has tumor-suppressive potential in APC-truncated colon cancer cells. Nat Commun, 9(1), 1106. doi:10.1038/s41467-018-03494-4
422. Radhakrishna, U., Wild, A., Grzeschik, K. H., & Antonarakis, S. E. (1997). Mutation in GLI3 in postaxial polydactyly type A. Nat Genet, 17(3), 269-271. doi:10.1038/ng1197-269
423. Ramkumar, N., Omelchenko, T., Silva-Gagliardi, N. F., McGlade, C. J., Wijnholds, J., & Anderson, K. V. (2016). Crumbs2 promotes cell ingression during the epithelial-to-mesenchymal transition at gastrulation. Nat Cell Biol, 18(12), 1281-1291. doi:10.1038/ncb3442
424. Rankin, C. T., Bunton, T., Lawler, A. M., & Lee, S. J. (2000). Regulation of left-right patterning in mice by growth/differentiation factor-1. Nat Genet, 24(3), 262-265. doi:10.1038/73472
425. Rasouli, S. J., & Stainier, D. Y. R. (2017). Regulation of cardiomyocyte behavior in zebrafish trabeculation by Neuregulin 2a signaling. Nat Commun, 8, 15281. doi:10.1038/ncomms15281
426. Ratcliff, R., Evans, M. J., Cuthbert, A. W., MacVinish, L. J., Foster, D., Anderson, J. R., & Colledge, W. H. (1993). Production of a severe cystic fibrosis mutation in mice by gene targeting. Nat Genet, 4(1), 35-41. doi:10.1038/ng0593-35
427. Razzaque, M. A., Nishizawa, T., Komoike, Y., Yagi, H., Furutani, M., Amo, R., . . . Matsuoka, R. (2007). Germline gain-of-function mutations in RAF1 cause Noonan syndrome. Nat Genet, 39(8), 1013-1017. doi:10.1038/ng2078
428. Regad, T., Roth, M., Bredenkamp, N., Illing, N., & Papalopulu, N. (2007). The neural progenitor-specifying activity of FoxG1 is antagonistically regulated by CKI and FGF. Nat Cell Biol, 9(5), 531-540. doi:10.1038/ncb1573
429. Reichow, S. L., Clemens, D. M., Freites, J. A., Nemeth-Cahalan, K. L., Heyden, M., Tobias, D. J., . . . Gonen, T. (2013). Allosteric mechanism of water-channel gating by Ca2+-calmodulin. Nat Struct Mol Biol, 20(9), 1085-1092. doi:10.1038/nsmb.2630
430. Reiner, O. (2002). Pathways of neuronal migration. Nat Genet, 32(3), 341-342. doi:10.1038/ng1102-341
431. Richardson, R. J., Dixon, J., Malhotra, S., Hardman, M. J., Knowles, L., Boot-Handford, R. P., . . . Dixon, M. J. (2006). Irf6 is a key determinant of the keratinocyte proliferation-differentiation switch. Nat Genet, 38(11), 1329-1334. doi:10.1038/ng1894
432. Rico, B., Beggs, H. E., Schahin-Reed, D., Kimes, N., Schmidt, A., & Reichardt, L. F. (2004). Control of axonal branching and synapse formation by focal adhesion kinase. Nat Neurosci, 7(10), 1059-1069. doi:10.1038/nn1317
433. Riviere, J. B., van Bon, B. W., Hoischen, A., Kholmanskikh, S. S., O'Roak, B. J., Gilissen, C., . . . Dobyns, W. B. (2012). De novo mutations in the actin genes ACTB and ACTG1 cause Baraitser-Winter syndrome. Nat Genet, 44(4), 440-444, s441-442. doi:10.1038/ng.1091
434. Rizzoti, K., Brunelli, S., Carmignac, D., Thomas, P. Q., Robinson, I. C., & Lovell-Badge, R. (2004). SOX3 is required during the formation of the hypothalamo-pituitary axis. Nat Genet, 36(3), 247-255. doi:10.1038/ng1309
435. Roder, K., Werdich, A. A., Li, W., Liu, M., Kim, T. Y., Organ-Darling, L. E., . . . Koren, G. (2014). RING finger protein RNF207, a novel regulator of cardiac excitation. J Biol Chem, 289(49), 33730-33740. doi:10.1074/jbc.M114.592295
436. Romano, D., Nguyen, L. K., Matallanas, D., Halasz, M., Doherty, C., Kholodenko, B. N., & Kolch, W. (2014). Protein interaction switches coordinate Raf-1 and MST2/Hippo signalling. Nat Cell Biol, 16(7), 673-684. doi:10.1038/ncb2986
437. Roots, I., Brockmoller, J., Drakoulis, N., & Loddenkemper, R. (1992). Mutant genes of cytochrome P-450IID6, glutathione S-transferase class Mu, and arylamine N-acetyltransferase in lung cancer patients. Clin Investig, 70(3-4), 307-319.
438. Roscioli, T., Kamsteeg, E. J., Buysse, K., Maystadt, I., van Reeuwijk, J., van den Elzen, C., . . . van Bokhoven, H. (2012). Mutations in ISPD cause Walker-Warburg syndrome and defective glycosylation of alpha-dystroglycan. Nat Genet, 44(5), 581-585. doi:10.1038/ng.2253
439. Rouiller, I., DeLaBarre, B., May, A. P., Weis, W. I., Brunger, A. T., Milligan, R. A., & Wilson-Kubalek, E. M. (2002). Conformational changes of the multifunction p97 AAA ATPase during its ATPase cycle. Nat Struct Biol, 9(12), 950-957. doi:10.1038/nsb872
440. Roukens, M. G., Alloul-Ramdhani, M., Baan, B., Kobayashi, K., Peterson-Maduro, J., van Dam, H., . . . Baker, D. A. (2010). Control of endothelial sprouting by a Tel-CtBP complex. Nat Cell Biol, 12(10), 933-942. doi:10.1038/ncb2096
441. Rugg, E. L., McLean, W. H., Allison, W. E., Lunny, D. P., Macleod, R. I., Felix, D. H., . . . Munro, C. S. (1995). A mutation in the mucosal keratin K4 is associated with oral white sponge nevus. Nat Genet, 11(4), 450-452. doi:10.1038/ng1295-450
442. Ruiz-Perez, V. L., Ide, S. E., Strom, T. M., Lorenz, B., Wilson, D., Woods, K., . . . Goodship, J. (2000). Mutations in a new gene in Ellis-van Creveld syndrome and Weyers acrodental dysostosis. Nat Genet, 24(3), 283-286. doi:10.1038/73508
443. Russell, I. J., Legan, P. K., Lukashkina, V. A., Lukashkin, A. N., Goodyear, R. J., & Richardson, G. P. (2007). Sharpened cochlear tuning in a mouse with a genetically modified tectorial membrane. Nat Neurosci, 10(2), 215-223. doi:10.1038/nn1828
444. Ryu, S. M., Koo, T., Kim, K., Lim, K., Baek, G., Kim, S. T., . . . Kim, J. S. (2018). Adenine base editing in mouse embryos and an adult mouse model of Duchenne muscular dystrophy. Nat Biotechnol, 36(6), 536-539. doi:10.1038/nbt.4148
445. Sagie, S., Toubiana, S., Hartono, S. R., Katzir, H., Tzur-Gilat, A., Havazelet, S., . . . Selig, S. (2017). Telomeres in ICF syndrome cells are vulnerable to DNA damage due to elevated DNA:RNA hybrids. Nat Commun, 8, 14015. doi:10.1038/ncomms14015
446. Sah, V. P., Attardi, L. D., Mulligan, G. J., Williams, B. O., Bronson, R. T., & Jacks, T. (1995). A subset of p53-deficient embryos exhibit exencephaly. Nat Genet, 10(2), 175-180. doi:10.1038/ng0695-175
447. Saito, R. M., Perreault, A., Peach, B., Satterlee, J. S., & van den Heuvel, S. (2004). The CDC-14 phosphatase controls developmental cell-cycle arrest in C. elegans. Nat Cell Biol, 6(8), 777-783. doi:10.1038/ncb1154
448. Sakai, D., & Trainor, P. A. (2009). Treacher Collins syndrome: unmasking the role of Tcof1/treacle. Int J Biochem Cell Biol, 41(6), 1229-1232. doi:10.1016/j.biocel.2008.10.026
449. Sant'Anna, R., Gallego, P., Robinson, L. Z., Pereira-Henriques, A., Ferreira, N., Pinheiro, F., . . . Ventura, S. (2016). Repositioning tolcapone as a potent inhibitor of transthyretin amyloidogenesis and associated cellular toxicity. Nat Commun, 7, 10787. doi:10.1038/ncomms10787
450. Santen, G. W., Aten, E., Sun, Y., Almomani, R., Gilissen, C., Nielsen, M., . . . Kriek, M. (2012). Mutations in SWI/SNF chromatin remodeling complex gene ARID1B cause Coffin-Siris syndrome. Nat Genet, 44(4), 379-380. doi:10.1038/ng.2217
451. Sanyanusin, P., Schimmenti, L. A., McNoe, L. A., Ward, T. A., Pierpont, M. E., Sullivan, M. J., . . . Eccles, M. R. (1995). Mutation of the PAX2 gene in a family with optic nerve colobomas, renal anomalies and vesicoureteral reflux. Nat Genet, 9(4), 358-364. doi:10.1038/ng0495-358
452. Sanyanusin, P., Schimmenti, L. A., McNoe, T. A., Ward, T. A., Pierpont, M. E., Sullivan, M. J., . . . Eccles, M. R. (1996). Mutation of the gene in a family with optic nerve colobomas, renal anomolies and vesicoureteral reflux. Nat Genet, 13(1), 129. doi:10.1038/ng0596-129
453. Sapkota, Y., Steinthorsdottir, V., Morris, A. P., Fassbender, A., Rahmioglu, N., De Vivo, I., . . . Nyholt, D. R. (2017). Meta-analysis identifies five novel loci associated with endometriosis highlighting key genes involved in hormone metabolism. Nat Commun, 8, 15539. doi:10.1038/ncomms15539
454. Sarparanta, J., Jonson, P. H., Golzio, C., Sandell, S., Luque, H., Screen, M., . . . Udd, B. (2012). Mutations affecting the cytoplasmic functions of the co-chaperone DNAJB6 cause limb-girdle muscular dystrophy. Nat Genet, 44(4), 450-455, s451-452. doi:10.1038/ng.1103
455. Satoda, M., Zhao, F., Diaz, G. A., Burn, J., Goodship, J., Davidson, H. R., . . . Gelb, B. D. (2000). Mutations in TFAP2B cause Char syndrome, a familial form of patent ductus arteriosus. Nat Genet, 25(1), 42-46. doi:10.1038/75578
456. Schaeffer, E. M., Yap, G. S., Lewis, C. M., Czar, M. J., McVicar, D. W., Cheever, A. W., . . . Schwartzberg, P. L. (2001). Mutation of Tec family kinases alters T helper cell differentiation. Nat Immunol, 2(12), 1183-1188. doi:10.1038/ni734
457. Schafer, K., & Braun, T. (1999). Early specification of limb muscle precursor cells by the homeobox gene Lbx1h. Nat Genet, 23(2), 213-216. doi:10.1038/13843
458. Schinke, M., & Izumo, S. (1999). Getting to the heart of DiGeorge syndrome. Nat Med, 5(10), 1120-1121. doi:10.1038/13438
459. Schinke, M., & Izumo, S. (2001). Deconstructing DiGeorge syndrome. Nat Genet, 27(3), 238-240. doi:10.1038/85784
460. Schmidt, M., Haas, W., Crosas, B., Santamaria, P. G., Gygi, S. P., Walz, T., & Finley, D. (2005). The HEAT repeat protein Blm10 regulates the yeast proteasome by capping the core particle. Nat Struct Mol Biol, 12(4), 294-303. doi:10.1038/nsmb914
461. Schnorrer, F., Ahlford, A., Chen, D., Milani, L., & Syvanen, A. C. (2008). Positional cloning by fast-track SNP-mapping in Drosophila melanogaster. Nat Protoc, 3(11), 1751-1765. doi:10.1038/nprot.2008.175
462. Schonberger, J., Wang, L., Shin, J. T., Kim, S. D., Depreux, F. F., Zhu, H., . . . Seidman, C. E. (2005). Mutation in the transcriptional coactivator EYA4 causes dilated cardiomyopathy and sensorineural hearing loss. Nat Genet, 37(4), 418-422. doi:10.1038/ng1527
463. Schuster, H., Wienker, T. E., Bahring, S., Bilginturan, N., Toka, H. R., Neitzel, H., . . . Luft, F. C. (1996). Severe autosomal dominant hypertension and brachydactyly in a unique Turkish kindred maps to human chromosome 12. Nat Genet, 13(1), 98-100. doi:10.1038/ng0596-98
464. Sehnert, A. J., Huq, A., Weinstein, B. M., Walker, C., Fishman, M., & Stainier, D. Y. (2002). Cardiac troponin T is essential in sarcomere assembly and cardiac contractility. Nat Genet, 31(1), 106-110. doi:10.1038/ng875
465. Selva, E. M., Hong, K., Baeg, G. H., Beverley, S. M., Turco, S. J., Perrimon, N., & Hacker, U. (2001). Dual role of the fringe connection gene in both heparan sulphate and fringe-dependent signalling events. Nat Cell Biol, 3(9), 809-815. doi:10.1038/ncb0901-809
466. Selvan, N., Williamson, R., Mariappa, D., Campbell, D. G., Gourlay, R., Ferenbach, A. T., . . . van Aalten, D. M. F. (2017). A mutant O-GlcNAcase enriches Drosophila developmental regulators. Nat Chem Biol, 13(8), 882-887. doi:10.1038/nchembio.2404
467. Semina, E. V., Ferrell, R. E., Mintz-Hittner, H. A., Bitoun, P., Alward, W. L., Reiter, R. S., . . . Murray, J. C. (1998). A novel homeobox gene PITX3 is mutated in families with autosomal-dominant cataracts and ASMD. Nat Genet, 19(2), 167-170. doi:10.1038/527
468. Sewell, B. T., Best, R. B., Chen, S., Roseman, A. M., Farr, G. W., Horwich, A. L., & Saibil, H. R. (2004). A mutant chaperonin with rearranged inter-ring electrostatic contacts and temperature-sensitive dissociation. Nat Struct Mol Biol, 11(11), 1128-1133. doi:10.1038/nsmb844
469. Shafique, S., Bajwa, R., & Shafique, S. (2009). Strain improvement and genetic characterization of indigenous Aspergillus flavus for amylolytic potential. Nat Prod Commun, 4(7), 977-980.
470. Shaw, N. D., Brand, H., Kupchinsky, Z. A., Bengani, H., Plummer, L., Jones, T. I., . . . Talkowski, M. E. (2017). SMCHD1 mutations associated with a rare muscular dystrophy can also cause isolated arhinia and Bosma arhinia microphthalmia syndrome. Nat Genet, 49(2), 238-248. doi:10.1038/ng.3743
471. Shi, S., Larson, K., Guo, D., Lim, S. J., Dutta, P., Yan, S. J., & Li, W. X. (2008). Drosophila STAT is required for directly maintaining HP1 localization and heterochromatin stability. Nat Cell Biol, 10(4), 489-496. doi:10.1038/ncb1713
472. Shi, X., Garcia, G., 3rd, Van De Weghe, J. C., McGorty, R., Pazour, G. J., Doherty, D., . . . Reiter, J. F. (2017). Super-resolution microscopy reveals that disruption of ciliary transition-zone architecture causes Joubert syndrome. Nat Cell Biol, 19(10), 1178-1188. doi:10.1038/ncb3599
473. Shioda, N., Yabuki, Y., Yamaguchi, K., Onozato, M., Li, Y., Kurosawa, K., . . . Fukunaga, K. (2018). Targeting G-quadruplex DNA as cognitive function therapy for ATR-X syndrome. Nat Med, 24(6), 802-813. doi:10.1038/s41591-018-0018-6
474. Sibon, O. C., Kelkar, A., Lemstra, W., & Theurkauf, W. E. (2000). DNA-replication/DNA-damage-dependent centrosome inactivation in Drosophila embryos. Nat Cell Biol, 2(2), 90-95. doi:10.1038/35000041
475. Sidi, S., & Look, A. T. (2005). Small molecules thwart crash and burn. Nat Chem Biol, 1(7), 351-353.
476. Sidow, A., Bulotsky, M. S., Kerrebrock, A. W., Birren, B. W., Altshuler, D., Jaenisch, R., . . . Lander, E. S. (1999). A novel member of the F-box/WD40 gene family, encoding dactylin, is disrupted in the mouse dactylaplasia mutant. Nat Genet, 23(1), 104-107. doi:10.1038/12709
477. Silver, D. L., Watkins-Chow, D. E., Schreck, K. C., Pierfelice, T. J., Larson, D. M., Burnetti, A. J., . . . Pavan, W. J. (2010). The exon junction complex component Magoh controls brain size by regulating neural stem cell division. Nat Neurosci, 13(5), 551-558. doi:10.1038/nn.2527
478. Simons, C., Rash, L. D., Crawford, J., Ma, L., Cristofori-Armstrong, B., Miller, D., . . . Taft, R. J. (2015). Mutations in the voltage-gated potassium channel gene KCNH1 cause Temple-Baraitser syndrome and epilepsy. Nat Genet, 47(1), 73-77. doi:10.1038/ng.3153
479. Sireteanu, A., Braha, E., Popescu, R., Gramescu, M., Gorduza, E. V., & Rusu, C. (2013). Inverted duplication deletion of 8P: characterization by standard cytogenetic and SNP array analyses. Rev Med Chir Soc Med Nat Iasi, 117(3), 731-734.
480. Smutny, M., Akos, Z., Grigolon, S., Shamipour, S., Ruprecht, V., Capek, D., . . . Heisenberg, C. P. (2017). Friction forces position the neural anlage. Nat Cell Biol, 19(4), 306-317. doi:10.1038/ncb3492
481. Sousa, S. B., Jenkins, D., Chanudet, E., Tasseva, G., Ishida, M., Anderson, G., . . . Moore, G. E. (2014). Gain-of-function mutations in the phosphatidylserine synthase 1 (PTDSS1) gene cause Lenz-Majewski syndrome. Nat Genet, 46(1), 70-76. doi:10.1038/ng.2829
482. Southard-Smith, E. M., Kos, L., & Pavan, W. J. (1998). Sox10 mutation disrupts neural crest development in Dom Hirschsprung mouse model. Nat Genet, 18(1), 60-64. doi:10.1038/ng0198-60
483. Spits, C., Mateizel, I., Geens, M., Mertzanidou, A., Staessen, C., Vandeskelde, Y., . . . Sermon, K. (2008). Recurrent chromosomal abnormalities in human embryonic stem cells. Nat Biotechnol, 26(12), 1361-1363. doi:10.1038/nbt.1510
484. Stainier, D. Y. (2001). Zebrafish genetics and vertebrate heart formation. Nat Rev Genet, 2(1), 39-48. doi:10.1038/35047564
485. Stalmans, I., Lambrechts, D., De Smet, F., Jansen, S., Wang, J., Maity, S., . . . Carmeliet, P. (2003). VEGF: a modifier of the del22q11 (DiGeorge) syndrome? Nat Med, 9(2), 173-182. doi:10.1038/nm819
486. Steinthorsdottir, V., Thorleifsson, G., Aradottir, K., Feenstra, B., Sigurdsson, A., Stefansdottir, L., . . . Stefansson, K. (2016). Common variants upstream of KDR encoding VEGFR2 and in TTC39B associate with endometriosis. Nat Commun, 7, 12350. doi:10.1038/ncomms12350
487. Stern, H. M., Murphey, R. D., Shepard, J. L., Amatruda, J. F., Straub, C. T., Pfaff, K. L., . . . Zon, L. I. (2005). Small molecules that delay S phase suppress a zebrafish bmyb mutant. Nat Chem Biol, 1(7), 366-370.
488. Stevenson, V. A., Kramer, J., Kuhn, J., & Theurkauf, W. E. (2001). Centrosomes and the Scrambled protein coordinate microtubule-independent actin reorganization. Nat Cell Biol, 3(1), 68-75. doi:10.1038/35050579
489. Stoffers, D. A., Zinkin, N. T., Stanojevic, V., Clarke, W. L., & Habener, J. F. (1997). Pancreatic agenesis attributable to a single nucleotide deletion in the human IPF1 gene coding sequence. Nat Genet, 15(1), 106-110. doi:10.1038/ng0197-106
490. Strebhardt, K., & Ullrich, A. (2008). Paul Ehrlich's magic bullet concept: 100 years of progress. Nat Rev Cancer, 8(6), 473-480. doi:10.1038/nrc2394
491. Suganuma, T., Gutierrez, J. L., Li, B., Florens, L., Swanson, S. K., Washburn, M. P., . . . Workman, J. L. (2008). ATAC is a double histone acetyltransferase complex that stimulates nucleosome sliding. Nat Struct Mol Biol, 15(4), 364-372. doi:10.1038/nsmb.1397
492. Sugden, W. W., Meissner, R., Aegerter-Wilmsen, T., Tsaryk, R., Leonard, E. V., Bussmann, J., . . . Siekmann, A. F. (2017). Endoglin controls blood vessel diameter through endothelial cell shape changes in response to haemodynamic cues. Nat Cell Biol, 19(6), 653-665. doi:10.1038/ncb3528
493. Svensson, E. C., Huggins, G. S., Lin, H., Clendenin, C., Jiang, F., Tufts, R., . . . Leiden, J. M. (2000). A syndrome of tricuspid atresia in mice with a targeted mutation of the gene encoding Fog-2. Nat Genet, 25(3), 353-356. doi:10.1038/77146
494. Takamiya, K., Kostourou, V., Adams, S., Jadeja, S., Chalepakis, G., Scambler, P. J., . . . Adams, R. H. (2004). A direct functional link between the multi-PDZ domain protein GRIP1 and the Fraser syndrome protein Fras1. Nat Genet, 36(2), 172-177. doi:10.1038/ng1292
495. Takeuchi, A., Mishina, Y., Miyaishi, O., Kojima, E., Hasegawa, T., & Isobe, K. (2003). Heterozygosity with respect to Zfp148 causes complete loss of fetal germ cells during mouse embryogenesis. Nat Genet, 33(2), 172-176. doi:10.1038/ng1072
496. Takeuchi, J. K., Lou, X., Alexander, J. M., Sugizaki, H., Delgado-Olguin, P., Holloway, A. K., . . . Bruneau, B. G. (2011). Chromatin remodelling complex dosage modulates transcription factor function in heart development. Nat Commun, 2, 187. doi:10.1038/ncomms1187
497. Tamura, T., Thibert, C., Royer, C., Kanda, T., Abraham, E., Kamba, M., . . . Couble, P. (2000). Germline transformation of the silkworm Bombyx mori L. using a piggyBac transposon-derived vector. Nat Biotechnol, 18(1), 81-84. doi:10.1038/71978
498. Tan, Y. Z., Aiyer, S., Mietzsch, M., Hull, J. A., McKenna, R., Grieger, J., . . . Lyumkis, D. (2018). Sub-2 A Ewald curvature corrected structure of an AAV2 capsid variant. Nat Commun, 9(1), 3628. doi:10.1038/s41467-018-06076-6
499. Tanaka, E., Taniguchi, A., Urano, W., Nakajima, H., Matsuda, Y., Kitamura, Y., . . . Kamatani, N. (2002). Adverse effects of sulfasalazine in patients with rheumatoid arthritis are associated with diplotype configuration at the N-acetyltransferase 2 gene. J Rheumatol, 29(12), 2492-2499.
500. Tanentzapf, G., Devenport, D., Godt, D., & Brown, N. H. (2007). Integrin-dependent anchoring of a stem-cell niche. Nat Cell Biol, 9(12), 1413-1418. doi:10.1038/ncb1660
501. Tanner, S. M., Aminoff, M., Wright, F. A., Liyanarachchi, S., Kuronen, M., Saarinen, A., . . . de la Chapelle, A. (2003). Amnionless, essential for mouse gastrulation, is mutated in recessive hereditary megaloblastic anemia. Nat Genet, 33(3), 426-429. doi:10.1038/ng1098
502. Tarpey, P., Thomas, S., Sarvananthan, N., Mallya, U., Lisgo, S., Talbot, C. J., . . . Gottlob, I. (2006). Mutations in FRMD7, a newly identified member of the FERM family, cause X-linked idiopathic congenital nystagmus. Nat Genet, 38(11), 1242-1244. doi:10.1038/ng1893
503. Taverna, E., Haffner, C., Pepperkok, R., & Huttner, W. B. (2011). A new approach to manipulate the fate of single neural stem cells in tissue. Nat Neurosci, 15(2), 329-337. doi:10.1038/nn.3008
504. Tavormina, P. L., Shiang, R., Thompson, L. M., Zhu, Y. Z., Wilkin, D. J., Lachman, R. S., . . . Wasmuth, J. J. (1995). Thanatophoric dysplasia (types I and II) caused by distinct mutations in fibroblast growth factor receptor 3. Nat Genet, 9(3), 321-328. doi:10.1038/ng0395-321
505. Tempfer, C. B., Schneeberger, C., & Huber, J. C. (2004). Applications of polymorphisms and pharmacogenomics in obstetrics and gynecology. Pharmacogenomics, 5(1), 57-65. doi:10.1517/phgs.5.1.57.25687
506. Teng, T., Tsai, J. H., Puyang, X., Seiler, M., Peng, S., Prajapati, S., . . . Zhu, P. (2017). Splicing modulators act at the branch point adenosine binding pocket defined by the PHF5A-SF3b complex. Nat Commun, 8, 15522. doi:10.1038/ncomms15522
507. Thijssen, P. E., Ito, Y., Grillo, G., Wang, J., Velasco, G., Nitta, H., . . . Sasaki, H. (2015). Mutations in CDCA7 and HELLS cause immunodeficiency-centromeric instability-facial anomalies syndrome. Nat Commun, 6, 7870. doi:10.1038/ncomms8870
508. Thivierge, C., Makil, N., Flamand, M., Vasale, J. J., Mello, C. C., Wohlschlegel, J., . . . Duchaine, T. F. (2011). Tudor domain ERI-5 tethers an RNA-dependent RNA polymerase to DCR-1 to potentiate endo-RNAi. Nat Struct Mol Biol, 19(1), 90-97. doi:10.1038/nsmb.2186
509. Tholen, M., Hillebrand, L. E., Tholen, S., Sedelmeier, O., Arnold, S. J., & Reinheckel, T. (2014). Out-of-frame start codons prevent translation of truncated nucleo-cytosolic cathepsin L in vivo. Nat Commun, 5, 4931. doi:10.1038/ncomms5931
510. Thomas, J. T., Kilpatrick, M. W., Lin, K., Erlacher, L., Lembessis, P., Costa, T., . . . Luyten, F. P. (1997). Disruption of human limb morphogenesis by a dominant negative mutation in CDMP1. Nat Genet, 17(1), 58-64. doi:10.1038/ng0997-58
511. Thompson, A. A., & Nguyen, L. T. (2000). Amegakaryocytic thrombocytopenia and radio-ulnar synostosis are associated with HOXA11 mutation. Nat Genet, 26(4), 397-398. doi:10.1038/82511
512. Thoms, M., Mitterer, V., Kater, L., Falquet, L., Beckmann, R., Kressler, D., & Hurt, E. (2018). Suppressor mutations in Rpf2-Rrs1 or Rpl5 bypass the Cgr1 function for pre-ribosomal 5S RNP-rotation. Nat Commun, 9(1), 4094. doi:10.1038/s41467-018-06660-w
513. Tirnoveanu, G., Selaru, T., Georgescu, M. I., Frangulea, V., & Mitescu, G. (1971). [Klinefelter's syndrome associated with total situs inversus and renal malformation]. Rev Med Chir Soc Med Nat Iasi, 75(3), 747-750.
514. Tirnoveanu, G., Tirnoveanu, M., Selaru, T., & Mitescu, G. (1974). Contributions to the study of genetic and immune factors in rheumatic fever. Rev Med Chir Soc Med Nat Iasi, 78(1), 49-55.
515. Tissir, F., Bar, I., Jossin, Y., De Backer, O., & Goffinet, A. M. (2005). Protocadherin Celsr3 is crucial in axonal tract development. Nat Neurosci, 8(4), 451-457. doi:10.1038/nn1428
516. Todosi, A. M., Gavrilescu, M. M., Anitei, G. M., Filip, B., & Scripcariu, V. (2012). Colon cancer at the molecular level--usefulness of epithelial-mesenchymal transition analysis. Rev Med Chir Soc Med Nat Iasi, 116(4), 1106-1111.
517. Tomancak, P., Piano, F., Riechmann, V., Gunsalus, K. C., Kemphues, K. J., & Ephrussi, A. (2000). A Drosophila melanogaster homologue of Caenorhabditis elegans par-1 acts at an early step in embryonic-axis formation. Nat Cell Biol, 2(7), 458-460. doi:10.1038/35017101
518. Tonkin, E. T., Wang, T. J., Lisgo, S., Bamshad, M. J., & Strachan, T. (2004). NIPBL, encoding a homolog of fungal Scc2-type sister chromatid cohesion proteins and fly Nipped-B, is mutated in Cornelia de Lange syndrome. Nat Genet, 36(6), 636-641. doi:10.1038/ng1363
519. Torpet, L. A., Kragelund, C., Reibel, J., & Nauntofte, B. (2004). ORAL ADVERSE DRUG REACTIONS TO CARDIOVASCULAR DRUGS. Crit Rev Oral Biol Med, 15(1), 28-46.
520. Towbin, J. A., & McQuinn, T. C. (1995). Gridlock: a model for coarctation of the aorta? Nat Med, 1(11), 1141-1142.
521. Townsley, F. M., Cliffe, A., & Bienz, M. (2004). Pygopus and Legless target Armadillo/beta-catenin to the nucleus to enable its transcriptional co-activator function. Nat Cell Biol, 6(7), 626-633. doi:10.1038/ncb1141
522. Traver, D., Paw, B. H., Poss, K. D., Penberthy, W. T., Lin, S., & Zon, L. I. (2003). Transplantation and in vivo imaging of multilineage engraftment in zebrafish bloodless mutants. Nat Immunol, 4(12), 1238-1246. doi:10.1038/ni1007
523. Tsitrin, Y., Morton, C. J., el-Bez, C., Paumard, P., Velluz, M. C., Adrian, M., . . . van der Goot, F. G. (2002). Conversion of a transmembrane to a water-soluble protein complex by a single point mutation. Nat Struct Biol, 9(10), 729-733. doi:10.1038/nsb839
524. Tsurusaki, Y., Koshimizu, E., Ohashi, H., Phadke, S., Kou, I., Shiina, M., . . . Matsumoto, N. (2014). De novo SOX11 mutations cause Coffin-Siris syndrome. Nat Commun, 5, 4011. doi:10.1038/ncomms5011
525. Tsurusaki, Y., Okamoto, N., Ohashi, H., Kosho, T., Imai, Y., Hibi-Ko, Y., . . . Matsumoto, N. (2012). Mutations affecting components of the SWI/SNF complex cause Coffin-Siris syndrome. Nat Genet, 44(4), 376-378. doi:10.1038/ng.2219
526. Turcot, V., Lu, Y., Highland, H. M., Schurmann, C., Justice, A. E., Fine, R. S., . . . Loos, R. J. F. (2018). Protein-altering variants associated with body mass index implicate pathways that control energy intake and expenditure in obesity. Nat Genet, 50(1), 26-41. doi:10.1038/s41588-017-0011-x
527. Tuschl, K., Meyer, E., Valdivia, L. E., Zhao, N., Dadswell, C., Abdul-Sada, A., . . . Wilson, S. W. (2016). Mutations in SLC39A14 disrupt manganese homeostasis and cause childhood-onset parkinsonism-dystonia. Nat Commun, 7, 11601. doi:10.1038/ncomms11601
528. Uddin, M. M., Ohigashi, I., Motosugi, R., Nakayama, T., Sakata, M., Hamazaki, J., . . . Takahama, Y. (2017). Foxn1-beta5t transcriptional axis controls CD8(+) T-cell production in the thymus. Nat Commun, 8, 14419. doi:10.1038/ncomms14419
529. Ueno, T., Tomita, J., Tanimoto, H., Endo, K., Ito, K., Kume, S., & Kume, K. (2012). Identification of a dopamine pathway that regulates sleep and arousal in Drosophila. Nat Neurosci, 15(11), 1516-1523. doi:10.1038/nn.3238
530. Unger, S., Bohm, D., Kaiser, F. J., Kaulfuss, S., Borozdin, W., Buiting, K., . . . Kohlhase, J. (2008). Mutations in the cyclin family member FAM58A cause an X-linked dominant disorder characterized by syndactyly, telecanthus and anogenital and renal malformations. Nat Genet, 40(3), 287-289. doi:10.1038/ng.86
531. Uno, S., Zembutsu, H., Hirasawa, A., Takahashi, A., Kubo, M., Akahane, T., . . . Nakamura, Y. (2010). A genome-wide association study identifies genetic variants in the CDKN2BAS locus associated with endometriosis in Japanese. Nat Genet, 42(8), 707-710. doi:10.1038/ng.612
532. Valente, E. M., Logan, C. V., Mougou-Zerelli, S., Lee, J. H., Silhavy, J. L., Brancati, F., . . . Gleeson, J. G. (2010). Mutations in TMEM216 perturb ciliogenesis and cause Joubert, Meckel and related syndromes. Nat Genet, 42(7), 619-625. doi:10.1038/ng.594
533. Valente, E. M., Silhavy, J. L., Brancati, F., Barrano, G., Krishnaswami, S. R., Castori, M., . . . Gleeson, J. G. (2006). Mutations in CEP290, which encodes a centrosomal protein, cause pleiotropic forms of Joubert syndrome. Nat Genet, 38(6), 623-625. doi:10.1038/ng1805
534. van Bokhoven, H., Celli, J., Kayserili, H., van Beusekom, E., Balci, S., Brussel, W., . . . Brunner, H. G. (2000). Mutation of the gene encoding the ROR2 tyrosine kinase causes autosomal recessive Robinow syndrome. Nat Genet, 25(4), 423-426. doi:10.1038/78113
535. van Bokhoven, H., Celli, J., van Reeuwijk, J., Rinne, T., Glaudemans, B., van Beusekom, E., . . . Brunner, H. G. (2005). MYCN haploinsufficiency is associated with reduced brain size and intestinal atresias in Feingold syndrome. Nat Genet, 37(5), 465-467. doi:10.1038/ng1546
536. van de Ven, J. P., Nilsson, S. C., Tan, P. L., Buitendijk, G. H., Ristau, T., Mohlin, F. C., . . . den Hollander, A. I. (2013). A functional variant in the CFI gene confers a high risk of age-related macular degeneration. Nat Genet, 45(7), 813-817. doi:10.1038/ng.2640
537. van Heemert, C. (1973). Androgenesis in the onionfly Hylemya antiqua (Meigen) demonstrated with a chromosomal marker. Nat New Biol, 246(149), 21-22.
538. van Karnebeek, C. D., Bonafe, L., Wen, X. Y., Tarailo-Graovac, M., Balzano, S., Royer-Bertrand, B., . . . Superti-Furga, A. (2016). NANS-mediated synthesis of sialic acid is required for brain and skeletal development. Nat Genet, 48(7), 777-784. doi:10.1038/ng.3578
539. Vanneste, E., Voet, T., Le Caignec, C., Ampe, M., Konings, P., Melotte, C., . . . Vermeesch, J. R. (2009). Chromosome instability is common in human cleavage-stage embryos. Nat Med, 15(5), 577-583. doi:10.1038/nm.1924
540. Varon, R., Gooding, R., Steglich, C., Marns, L., Tang, H., Angelicheva, D., . . . Kalaydjieva, L. (2003). Partial deficiency of the C-terminal-domain phosphatase of RNA polymerase II is associated with congenital cataracts facial dysmorphism neuropathy syndrome. Nat Genet, 35(2), 185-189. doi:10.1038/ng1243
541. Veleri, S., Manjunath, S. H., Fariss, R. N., May-Simera, H., Brooks, M., Foskett, T. A., . . . Swaroop, A. (2014). Ciliopathy-associated gene Cc2d2a promotes assembly of subdistal appendages on the mother centriole during cilia biogenesis. Nat Commun, 5, 4207. doi:10.1038/ncomms5207
542. Venetucci, L., Denegri, M., Napolitano, C., & Priori, S. G. (2012). Inherited calcium channelopathies in the pathophysiology of arrhythmias. Nat Rev Cardiol, 9(10), 561-575. doi:10.1038/nrcardio.2012.93
543. Villares, R., Gutierrez, J., Futterer, A., Trachana, V., Gutierrez del Burgo, F., & Martinez, A. C. (2015). Dido mutations trigger perinatal death and generate brain abnormalities and behavioral alterations in surviving adult mice. Proc Natl Acad Sci U S A, 112(15), 4803-4808. doi:10.1073/pnas.1419300112
544. Vits, L., Van Camp, G., Coucke, P., Fransen, E., De Boulle, K., Reyniers, E., . . . et al. (1994). MASA syndrome is due to mutations in the neural cell adhesion gene L1CAM. Nat Genet, 7(3), 408-413. doi:10.1038/ng0794-408
545. Vivian, J. L., Chen, Y., Yee, D., Schneider, E., & Magnuson, T. (2002). An allelic series of mutations in Smad2 and Smad4 identified in a genotype-based screen of N-ethyl-N- nitrosourea-mutagenized mouse embryonic stem cells. Proc Natl Acad Sci U S A, 99(24), 15542-15547. doi:10.1073/pnas.242474199
546. von Schack, D., Casademunt, E., Schweigreiter, R., Meyer, M., Bibel, M., & Dechant, G. (2001). Complete ablation of the neurotrophin receptor p75NTR causes defects both in the nervous and the vascular system. Nat Neurosci, 4(10), 977-978. doi:10.1038/nn730
547. Wada, T., Joza, N., Cheng, H. Y., Sasaki, T., Kozieradzki, I., Bachmaier, K., . . . Penninger, J. M. (2004). MKK7 couples stress signalling to G2/M cell-cycle progression and cellular senescence. Nat Cell Biol, 6(3), 215-226. doi:10.1038/ncb1098
548. Wakefield, L., Long, H., Lack, N., & Sim, E. (2007). Ocular defects associated with a null mutation in the mouse arylamine N-acetyltransferase 2 gene. Mamm Genome, 18(4), 270-276. doi:10.1007/s00335-007-9010-z
549. Wall, M. E., Wani, M. C., Manikumar, G., Taylor, H., & McGivney, R. (1989). Plant antimutagens, 6. Intricatin and intricatinol, new antimutagenic homoisoflavonoids from Hoffmanosseggia intricata. J Nat Prod, 52(4), 774-778.
550. Wang, F., Burrage, A. M., Postel, S., Clark, R. E., Orlova, A., Sundberg, E. J., . . . Egelman, E. H. (2017). A structural model of flagellar filament switching across multiple bacterial species. Nat Commun, 8(1), 960. doi:10.1038/s41467-017-01075-5
551. Wang, M., Quinn, C. M., Perilla, J. R., Zhang, H., Shirra, R., Jr., Hou, G., . . . Polenova, T. (2017). Quenching protein dynamics interferes with HIV capsid maturation. Nat Commun, 8(1), 1779. doi:10.1038/s41467-017-01856-y
552. Wang, W., Jossin, Y., Chai, G., Lien, W. H., Tissir, F., & Goffinet, A. M. (2016). Feedback regulation of apical progenitor fate by immature neurons through Wnt7-Celsr3-Fzd3 signalling. Nat Commun, 7, 10936. doi:10.1038/ncomms10936
553. Wang, X., Li, S. H., Zhu, L., Nian, Q. G., Yuan, S., Gao, Q., . . . Rao, Z. (2017). Near-atomic structure of Japanese encephalitis virus reveals critical determinants of virulence and stability. Nat Commun, 8(1), 14. doi:10.1038/s41467-017-00024-6
554. Wang, Y. K., Bashashati, A., Anglesio, M. S., Cochrane, D. R., Grewal, D. S., Ha, G., . . . Shah, S. P. (2017). Genomic consequences of aberrant DNA repair mechanisms stratify ovarian cancer histotypes. Nat Genet, 49(6), 856-865. doi:10.1038/ng.3849
555. Watanabe, H., & Yamada, Y. (1999). Mice lacking link protein develop dwarfism and craniofacial abnormalities. Nat Genet, 21(2), 225-229. doi:10.1038/6016
556. Watanabe, S., Tan, D., Lakshminarasimhan, M., Washburn, M. P., Hong, E. J., Walz, T., & Peterson, C. L. (2015). Structural analyses of the chromatin remodelling enzymes INO80-C and SWR-C. Nat Commun, 6, 7108. doi:10.1038/ncomms8108
557. Weedon, M. N., Cebola, I., Patch, A. M., Flanagan, S. E., De Franco, E., Caswell, R., . . . Hattersley, A. T. (2014). Recessive mutations in a distal PTF1A enhancer cause isolated pancreatic agenesis. Nat Genet, 46(1), 61-64. doi:10.1038/ng.2826
558. Weinstein, B. M., Stemple, D. L., Driever, W., & Fishman, M. C. (1995). Gridlock, a localized heritable vascular patterning defect in the zebrafish. Nat Med, 1(11), 1143-1147.
559. Weninger, W. J., & Mohun, T. (2002). Phenotyping transgenic embryos: a rapid 3-D screening method based on episcopic fluorescence image capturing. Nat Genet, 30(1), 59-65. doi:10.1038/ng785
560. Wertheimer, E., Lu, S. P., Backeljauw, P. F., Davenport, M. L., & Taylor, S. I. (1993). Homozygous deletion of the human insulin receptor gene results in leprechaunism. Nat Genet, 5(1), 71-73. doi:10.1038/ng0993-71
561. Weyemi, U., Redon, C. E., Choudhuri, R., Aziz, T., Maeda, D., Boufraqech, M., . . . Bonner, W. M. (2016). The histone variant H2A.X is a regulator of the epithelial-mesenchymal transition. Nat Commun, 7, 10711. doi:10.1038/ncomms10711
562. Wheway, G., Schmidts, M., Mans, D. A., Szymanska, K., Nguyen, T. T., Racher, H., . . . Johnson, C. A. (2015). An siRNA-based functional genomics screen for the identification of regulators of ciliogenesis and ciliopathy genes. Nat Cell Biol, 17(8), 1074-1087. doi:10.1038/ncb3201
563. Wienholds, E., Koudijs, M. J., van Eeden, F. J., Cuppen, E., & Plasterk, R. H. (2003). The microRNA-producing enzyme Dicer1 is essential for zebrafish development. Nat Genet, 35(3), 217-218. doi:10.1038/ng1251
564. Wigle, J. T., Chowdhury, K., Gruss, P., & Oliver, G. (1999). Prox1 function is crucial for mouse lens-fibre elongation. Nat Genet, 21(3), 318-322. doi:10.1038/6844
565. Wild, R., Klems, A., Takamiya, M., Hayashi, Y., Strahle, U., Ando, K., . . . le Noble, F. (2017). Neuronal sFlt1 and Vegfaa determine venous sprouting and spinal cord vascularization. Nat Commun, 8, 13991. doi:10.1038/ncomms13991
566. Wilkie, A. O. (2017). Many faces of SMCHD1. Nat Genet, 49(2), 176-178. doi:10.1038/ng.3776
567. Wilkie, A. O., & Morriss-Kay, G. M. (2001). Genetics of craniofacial development and malformation. Nat Rev Genet, 2(6), 458-468. doi:10.1038/35076601
568. Wilkie, A. O., Tang, Z., Elanko, N., Walsh, S., Twigg, S. R., Hurst, J. A., . . . Maxson, R. E., Jr. (2000). Functional haploinsufficiency of the human homeobox gene MSX2 causes defects in skull ossification. Nat Genet, 24(4), 387-390. doi:10.1038/74224
569. Will, A. J., Cova, G., Osterwalder, M., Chan, W. L., Wittler, L., Brieske, N., . . . Mundlos, S. (2017). Composition and dosage of a multipartite enhancer cluster control developmental expression of Ihh (Indian hedgehog). Nat Genet, 49(10), 1539-1545. doi:10.1038/ng.3939
570. Windpassinger, C., Auer-Grumbach, M., Irobi, J., Patel, H., Petek, E., Horl, G., . . . Wagner, K. (2004). Heterozygous missense mutations in BSCL2 are associated with distal hereditary motor neuropathy and Silver syndrome. Nat Genet, 36(3), 271-276. doi:10.1038/ng1313
571. Wohrle, D., Hennig, I., Vogel, W., & Steinbach, P. (1993). Mitotic stability of fragile X mutations in differentiated cells indicates early post-conceptional trinucleotide repeat expansion. Nat Genet, 4(2), 140-142. doi:10.1038/ng0693-140
572. Wood, W., Jacinto, A., Grose, R., Woolner, S., Gale, J., Wilson, C., & Martin, P. (2002). Wound healing recapitulates morphogenesis in Drosophila embryos. Nat Cell Biol, 4(11), 907-912. doi:10.1038/ncb875
573. Wright, E., Hargrave, M. R., Christiansen, J., Cooper, L., Kun, J., Evans, T., . . . Koopman, P. (1995). The Sry-related gene Sox9 is expressed during chondrogenesis in mouse embryos. Nat Genet, 9(1), 15-20. doi:10.1038/ng0195-15
574. Wu, C. H., Guo, C. Y., Yang, J. G., Tsai, H. D., Chang, Y. J., Tsai, P. C., . . . Kuo, P. L. (2012). Polymorphisms of dioxin receptor complex components and detoxification-related genes jointly confer susceptibility to advanced-stage endometriosis in the taiwanese han population. Am J Reprod Immunol, 67(2), 160-168. doi:10.1111/j.1600-0897.2011.01077.x
575. Xu, C. F., Lewis, K., Cantone, K. L., Khan, P., Donnelly, C., White, N., . . . Purvis, I. J. (2002). Effectiveness of computational methods in haplotype prediction. Hum Genet, 110(2), 148-156. doi:10.1007/s00439-001-0656-4
576. Xu, M., Horrell, J., Snitow, M., Cui, J., Gochnauer, H., Syrett, C. M., . . . Millar, S. E. (2017). WNT10A mutation causes ectodermal dysplasia by impairing progenitor cell proliferation and KLF4-mediated differentiation. Nat Commun, 8, 15397. doi:10.1038/ncomms15397
577. Xu, W., Edmondson, D. G., Evrard, Y. A., Wakamiya, M., Behringer, R. R., & Roth, S. Y. (2000). Loss of Gcn5l2 leads to increased apoptosis and mesodermal defects during mouse development. Nat Genet, 26(2), 229-232. doi:10.1038/79973
578. Xu, X., Meiler, S. E., Zhong, T. P., Mohideen, M., Crossley, D. A., Burggren, W. W., & Fishman, M. C. (2002). Cardiomyopathy in zebrafish due to mutation in an alternatively spliced exon of titin. Nat Genet, 30(2), 205-209. doi:10.1038/ng816
579. Xu, X., Shi, Y., Zhang, H. M., Swindell, E. C., Marshall, A. G., Guo, M., . . . Yang, X. L. (2012). Unique domain appended to vertebrate tRNA synthetase is essential for vascular development. Nat Commun, 3, 681. doi:10.1038/ncomms1686
580. Yang, B., Zhou, W., Jiao, J., Nielsen, J. B., Mathis, M. R., Heydarpour, M., . . . Willer, C. J. (2017). Protein-altering and regulatory genetic variants near GATA4 implicated in bicuspid aortic valve. Nat Commun, 8, 15481. doi:10.1038/ncomms15481
581. Yang, F., Xiao, X., Cheng, W., Yang, W., Yu, P., Song, Z., . . . Zheng, J. (2015). Structural mechanism underlying capsaicin binding and activation of the TRPV1 ion channel. Nat Chem Biol, 11(7), 518-524. doi:10.1038/nchembio.1835
582. Yao, Y., Wu, Y., Yin, C., Ozawa, R., Aigaki, T., Wouda, R. R., . . . Hing, H. (2007). Antagonistic roles of Wnt5 and the Drl receptor in patterning the Drosophila antennal lobe. Nat Neurosci, 10(11), 1423-1432. doi:10.1038/nn1993
583. Ye, J., Chen, S., & Maniatis, T. (2011). Cardiac glycosides are potent inhibitors of interferon-beta gene expression. Nat Chem Biol, 7(1), 25-33. doi:10.1038/nchembio.476
584. Yoda, S., Yamaguchi, J., Mita, K., Yamamoto, K., Banno, Y., Ando, T., . . . Fujiwara, H. (2014). The transcription factor Apontic-like controls diverse colouration pattern in caterpillars. Nat Commun, 5, 4936. doi:10.1038/ncomms5936
585. Yokoi, T., Sawada, M., & Kamataki, T. (1995). Polymorphic drug metabolism: studies with recombinant Chinese hamster cells and analyses in human populations. Pharmacogenetics, 5 Spec No, S65-69.
586. Yoon, K., & Gaiano, N. (2005). Notch signaling in the mammalian central nervous system: insights from mouse mutants. Nat Neurosci, 8(6), 709-715. doi:10.1038/nn1475
587. Yoshida, M., Hata, K., Takashima, R., Ono, K., Nakamura, E., Takahata, Y., . . . Yoneda, T. (2015). The transcription factor Foxc1 is necessary for Ihh-Gli2-regulated endochondral ossification. Nat Commun, 6, 6653. doi:10.1038/ncomms7653
588. Yoshizawa, T., Handa, Y., Uematsu, Y., Takeda, S., Sekine, K., Yoshihara, Y., . . . Kato, S. (1997). Mice lacking the vitamin D receptor exhibit impaired bone formation, uterine hypoplasia and growth retardation after weaning. Nat Genet, 16(4), 391-396. doi:10.1038/ng0897-391
589. Yu, H., Ito, T., Wellmer, F., & Meyerowitz, E. M. (2004). Repression of AGAMOUS-LIKE 24 is a crucial step in promoting flower development. Nat Genet, 36(2), 157-161. doi:10.1038/ng1286
590. Yu, T. W., Mochida, G. H., Tischfield, D. J., Sgaier, S. K., Flores-Sarnat, L., Sergi, C. M., . . . Walsh, C. A. (2010). Mutations in WDR62, encoding a centrosome-associated protein, cause microcephaly with simplified gyri and abnormal cortical architecture. Nat Genet, 42(11), 1015-1020. doi:10.1038/ng.683
591. Yuasa-Kawada, J., Kinoshita-Kawada, M., Wu, G., Rao, Y., & Wu, J. Y. (2009). Midline crossing and Slit responsiveness of commissural axons require USP33. Nat Neurosci, 12(9), 1087-1089. doi:10.1038/nn.2382
592. Yuchi, Z., Yuen, S. M., Lau, K., Underhill, A. Q., Cornea, R. L., Fessenden, J. D., & Van Petegem, F. (2015). Crystal structures of ryanodine receptor SPRY1 and tandem-repeat domains reveal a critical FKBP12 binding determinant. Nat Commun, 6, 7947. doi:10.1038/ncomms8947
593. Zenker, M., Mayerle, J., Lerch, M. M., Tagariello, A., Zerres, K., Durie, P. R., . . . Reis, A. (2005). Deficiency of UBR1, a ubiquitin ligase of the N-end rule pathway, causes pancreatic dysfunction, malformations and mental retardation (Johanson-Blizzard syndrome). Nat Genet, 37(12), 1345-1350. doi:10.1038/ng1681
594. Zhang, F., Thomas, L. R., Oltz, E. M., & Aune, T. M. (2006). Control of thymocyte development and recombination-activating gene expression by the zinc finger protein Zfp608. Nat Immunol, 7(12), 1309-1316. doi:10.1038/ni1397
595. Zhang, L., Zhou, F., Drabsch, Y., Gao, R., Snaar-Jagalska, B. E., Mickanin, C., . . . ten Dijke, P. (2012). USP4 is regulated by AKT phosphorylation and directly deubiquitylates TGF-beta type I receptor. Nat Cell Biol, 14(7), 717-726. doi:10.1038/ncb2522
596. Zhang, Y. B., Hu, J., Zhang, J., Zhou, X., Li, X., Gu, C., . . . Zhang, Q. (2016). Genome-wide association study identifies multiple susceptibility loci for craniofacial microsomia. Nat Commun, 7, 10605. doi:10.1038/ncomms10605
597. Zhang, Z., Lee, J. C., Lin, L., Olivas, V., Au, V., LaFramboise, T., . . . Bivona, T. G. (2012). Activation of the AXL kinase causes resistance to EGFR-targeted therapy in lung cancer. Nat Genet, 44(8), 852-860. doi:10.1038/ng.2330
598. Zhao, Q., Behringer, R. R., & de Crombrugghe, B. (1996). Prenatal folic acid treatment suppresses acrania and meroanencephaly in mice mutant for the Cart1 homeobox gene. Nat Genet, 13(3), 275-283. doi:10.1038/ng0796-275
599. Zhao, S., Xia, J., Wu, X., Zhang, L., Wang, P., Wang, H., . . . Shu, X. (2018). Deficiency in class III PI3-kinase confers postnatal lethality with IBD-like features in zebrafish. Nat Commun, 9(1), 2639. doi:10.1038/s41467-018-05105-8
600. Zhao, X. Y., Lv, Z., Li, W., Zeng, F., & Zhou, Q. (2010). Production of mice using iPS cells and tetraploid complementation. Nat Protoc, 5(5), 963-971. doi:10.1038/nprot.2010.61
601. Zheng, W., Yang, X., Hu, R., Cai, R., Hofmann, L., Wang, Z., . . . Chen, X. Z. (2018). Hydrophobic pore gates regulate ion permeation in polycystic kidney disease 2 and 2L1 channels. Nat Commun, 9(1), 2302. doi:10.1038/s41467-018-04586-x
602. Zhou, B. P., Deng, J., Xia, W., Xu, J., Li, Y. M., Gunduz, M., & Hung, M. C. (2004). Dual regulation of Snail by GSK-3beta-mediated phosphorylation in control of epithelial-mesenchymal transition. Nat Cell Biol, 6(10), 931-940. doi:10.1038/ncb1173
603. Zhou, W., Otto, E. A., Cluckey, A., Airik, R., Hurd, T. W., Chaki, M., . . . Hildebrandt, F. (2012). FAN1 mutations cause karyomegalic interstitial nephritis, linking chronic kidney failure to defective DNA damage repair. Nat Genet, 44(8), 910-915. doi:10.1038/ng.2347
604. Zhu, X. N., Liu, X. D., Sun, S., Zhuang, H., Yang, J. Y., Henkemeyer, M., & Xu, N. J. (2016). Ephrin-B3 coordinates timed axon targeting and amygdala spinogenesis for innate fear behaviour. Nat Commun, 7, 11096. doi:10.1038/ncomms11096

Excluded for being review:

1. Guo, S. W. (2006). The association of endometriosis risk and genetic polymorphisms involving dioxin detoxification enzymes: a systematic review. Eur J Obstet Gynecol Reprod Biol, 124(2), 134-143. doi:10.1016/j.ejogrb.2005.10.002

Excluded for not reporting exact genotye gene:

1. Matsuzaka, Y., Kikuti, Y. Y., Goya, K., Suzuki, T., Cai, L. Y., Oka, A., . . . Kimura, M. (2012). Lack of an association human dioxin detoxification gene polymorphisms with endometriosis in Japanese women: results of a pilot study. Environ Health Prev Med, 17(6), 512-517. doi:10.1007/s12199-012-0281-y
2. Wang,Y. (2009) Correlation between NAT2 gene and inheritance of endometriosis. Chin Mod Doctor, 2009;47(10):146-47 doi: doi:10.3969/j.issn.1673-9701.2009.10.088[published Online First: Epub Date]|.

Excluded for single-arm study:

1. Dubinskaia ED, Gasparov AS, Fedorova TA, Lapteva NV. (2013) Role of the genetic factors, detoxication systems and oxidative stress in the pathogenesis of endometriosis and infertility (review). Vestn Ross Akad Med Nauk, 2013(8):14-9
