# Supplementary material for: NAT2 gene polymorphisms and endometriosis risk: A PRISMA-compliant meta-analysis
Source: PLoS One. 2019 Dec 27;14(12):e0227043. doi: 10.1371/journal.pone.0227043 (PMC6934289; doi:10.1371/journal.pone.0227043)
Supplement: S4 File — (DOC) [file pone.0227043.s004.doc]

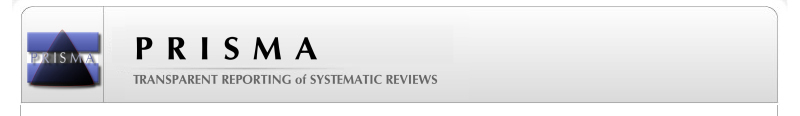
**PRISMA Flow Diagram**

**Screening**

**Included**

**Eligibility**

**Identification**

Records identified through database searching
(n = 617)

Additional records identified through other sources
(n =0)

Records after duplicates removed
(n =13)

Records screened
(n =13)

Records excluded
(n =2)

Full-text articles assessed for eligibility
(n =11)

Full-text articles excluded, with reasons
(n =2

Studies included in qualitative synthesis
(n =9)

Studies included in quantitative synthesis (meta-analysis)
(n =9)
